# Polyphosphate Dynamics in Cable Bacteria

**DOI:** 10.3389/fmicb.2022.883807

**Published:** 2022-05-19

**Authors:** Nicole M. J. Geerlings, Michiel V. M. Kienhuis, Silvia Hidalgo-Martinez, Renee Hageman, Diana Vasquez-Cardenas, Jack J. Middelburg, Filip J. R. Meysman, Lubos Polerecky

**Affiliations:** ^1^Department of Earth Sciences, Utrecht University, Utrecht, Netherlands; ^2^Excellence centre for Microbial Systems Technology, University of Antwerp, Wilrijk, Belgium; ^3^Department of Biotechnology, Delft University of Technology, Delft, Netherlands

**Keywords:** cable bacteria, polyphosphate, nanoSIMS, stable isotope probing, cell cycle

## Abstract

Cable bacteria are multicellular sulfide oxidizing bacteria that display a unique metabolism based on long-distance electron transport. Cells in deeper sediment layers perform the sulfide oxidizing half-reaction whereas cells in the surface layers of the sediment perform the oxygen-reducing half-reaction. These half-reactions are coupled *via* electron transport through a conductive fiber network that runs along the shared cell envelope. Remarkably, only the sulfide oxidizing half-reaction is coupled to biosynthesis and growth whereas the oxygen reducing half-reaction serves to rapidly remove electrons from the conductive fiber network and is not coupled to energy generation and growth. Cells residing in the oxic zone are believed to (temporarily) rely on storage compounds of which polyphosphate (poly-P) is prominently present in cable bacteria. Here we investigate the role of poly-P in the metabolism of cable bacteria within the different redox environments. To this end, we combined nanoscale secondary ion mass spectrometry with dual-stable isotope probing (^13^C-DIC and ^18^O-H_2_O) to visualize the relationship between growth in the cytoplasm (^13^C-enrichment) and poly-P activity (^18^O-enrichment). We found that poly-P was synthesized in almost all cells, as indicated by ^18^O enrichment of poly-P granules. Hence, poly-P must have an important function in the metabolism of cable bacteria. Within the oxic zone of the sediment, where little growth is observed, ^18^O enrichment in poly-P granules was significantly lower than in the suboxic zone. Thus, both growth and poly-P metabolism appear to be correlated to the redox environment. However, the poly-P metabolism is not coupled to growth in cable bacteria, as many filaments from the suboxic zone showed poly-P activity but did not grow. We hypothesize that within the oxic zone, poly-P is used to protect the cells against oxidative stress and/or as a resource to support motility, while within the suboxic zone, poly-P is involved in the metabolic regulation before cells enter a non-growing stage.

## Introduction

Cable bacteria are long, unbranched filamentous microorganisms consisting of thousands of cells that metabolically cooperate through electrical currents (Pfeffer et al., 2012). A given filament spatially couples sulfide oxidation (H_2_S + 4 H_2_O→SO_4_^2–^ + 10 H^+^ + 8 e^–^) in deeper sediment layers to oxygen reduction (O_2_ + 4 H^+^ + 4 e^–^→4 H_2_O) at the sediment-water interface via a process termed long-distance electron transport (Nielsen et al., 2010; Pfeffer et al., 2012). These two redox half-reactions are thus occurring in different cells of the same filament, with the necessary electrical coupling ensured by the transport of electrons over centimeter-scale distances through a conductive fiber network that runs internally (i.e., within the shared periplasmic space) along the entire filament (Meysman et al., 2019; Thiruvallur Eachambadi et al., 2020). This spatial separation of redox half-reactions gives cable bacteria a competitive advantage over other sulfide-oxidizing bacteria, because it allows them to harvest energy from aerobic sulfide oxidation even though free sulfide is spatially separated from molecular oxygen by centimeter-scale distances (Pfeffer et al., 2012; Risgaard-Petersen et al., 2012; Meysman, 2018).

Cable bacteria are facultative autotrophs that mainly assimilate inorganic CO_2_ via the Wood-Ljungdahl pathway, but can also assimilate propionate (Vasquez-Cardenas et al., 2015; Kjeldsen et al., 2019; Geerlings et al., 2020). They are found in a wide range of aquatic sediment environments including marine (Malkin et al., 2014; Burdorf et al., 2017), freshwater (Risgaard-Petersen et al., 2015), and aquifer (Müller et al., 2016) sediments. They have also been found in association with oxygenated zones around plant roots (Scholz et al., 2019) and worm tubes in marine sediments (Aller et al., 2019), or attached to the anode of a benthic microbial fuel cell placed in anaerobic conditions (Reimers et al., 2017).

A conspicuous aspect of the metabolism of cable bacteria is that the metabolic energy harvested through long-distance electron transport is not made equally available to all cells within a filament (Geerlings et al., 2020). Specifically, cable bacteria filaments display a remarkable division of “energy rewards,” in which only the sulfide oxidizing cells gain enough energy for biosynthesis and growth, whereas the oxygen reducing cells dispense electrons as quickly as possible without biosynthesis and growth (Kjeldsen et al., 2019; Geerlings et al., 2020). Therefore, the oxygen reducing cells appear to provide a kind of “community service” to the filament by ensuring that the electrical current can flow, but only facilitates the growth of the sulfide oxidizing cells (Geerlings et al., 2020).

To maintain their function, oxygen reducing cells have been hypothesized to temporarily rely on storage compounds, of which polyphosphate (poly-P) is the most ubiquitous within cable bacteria (Kjeldsen et al., 2019; Geerlings et al., 2019). For example, poly-P granules have been observed in both marine (Sulu-Gambari et al., 2016; Geerlings et al., 2019) and freshwater (Kjeldsen et al., 2019) cable bacteria. The size and density of the granules widely vary among filaments from the same redox environment and to a lesser extent also within individual filaments (Geerlings et al., 2019).

Poly-P is a ubiquitous inorganic biopolymer consisting of tens to hundreds of phosphate residues linearly linked together by the same high-energy phosphoanhydride bond that is also found in ATP (Rao et al., 2009). Poly-P granules are found in cells across all three domains of life (Rao et al., 2009) and were actually one of the first subcellular structures described in bacteria (Meyer, 1904). The enzymes involved in poly-P metabolism are highly conserved (Rao et al., 2009), and it is believed that poly-P has played a key role in the origin of life (Brown and Kornberg, 2004; Achbergerová and Nahálka, 2011). In microbial cells, poly-P appears to have distinctive biological functions depending on the abundance, chain length, biological source, and subcellular location of the granules. It is thought to act as an ATP substitute and energy storage molecule, although the metabolic turnover of ATP is considerably faster than that of poly-P (Kornberg, 1995; Ault-Riché et al., 1998). Poly-P granules can also serve as reservoir for orthophosphate (P_*i*_). Due to their anionic nature, poly-P molecules typically form complexes with cations, so they can also function as a chelator of metal ions and a buffer against alkali ions (Kornberg, 1995; Seufferheld et al., 2008; Rao et al., 2009). Finally, poly-P has been claimed to aid the channeling of DNA from the environment into the cell and appears to regulate the responses to stresses and adjustments for survival, especially in the stationary phase of culture growth and development (Kornberg, 1995; Rao and Kornberg, 1996; Ault-Riché et al., 1998; Rao et al., 1998). Recent research has demonstrated that poly-P chains can also function as a protein chaperone during stress conditions, where a poly-P chain counteracts irreversible protein aggregation by stabilizing proteins and maintaining them in a refolding-competent formation (Gray et al., 2014).

In cable bacteria, it was hypothesized that poly-P acts as a “survival energy package” for cells that glide into the oxic zone when performing “community service”, thus functioning as a substitute for ATP or as a protection against oxidative stress (Kjeldsen et al., 2019; Geerlings et al., 2020). Indeed, differences in the relative phosphorus content (i.e., cellular P/C) between cells residing in the suboxic and oxic zone have been observed and attributed to a build-up of poly-P in the suboxic zone and a breakdown of poly-P in the oxic zone (Geerlings et al., 2020). However, this hypothesis needs further testing, as other roles for poly-P are possible. For example, it has been argued that poly-P can be involved in Ca^2+^/H^+^ homeostasis to maintain optimum intracellular pH levels in the alkaline oxic zone (Geerlings et al., 2019), or act as an internal energy storage that drives the motility of cable bacteria (Bjerg et al., 2016). In this research we aim to assess the dynamics of poly-P in individual cells of cable bacteria and explore how this data can help us further elucidate the possible role(s) of poly-P in cable bacteria, both in the oxic and suboxic zone of the sediment.

Assessment of poly-P dynamics in cable bacteria is hampered by methodological challenges. Stable isotopes exist for elements such as carbon and nitrogen, which allows tracing of metabolic pathways involving these elements on a single-cell level using stable isotope probing (SIP) combined with nanoscale secondary ion mass spectrometry (nanoSIMS) (Musat et al., 2016). However, P can only be traced through radiolabelling that involves the addition of the short-lived ^33^P isotope and a subsequent quantification of the enrichment in the daughter isotope ^33^S (Schoffelen et al., 2018), since it only has one stable isotope.

Recently, an indirect method was applied to study poly-P metabolism in bacteria, which utilizes SIP with ^18^O-labeled water (H_2_^18^O) in combination with nanoSIMS (Langer et al., 2018). This method exploits the relatively rapid exchange of O-atoms between phosphate and water molecules catalyzed by enzymes. Still, labeled O-atoms from water molecules can also be incorporated into proteins and other molecules. Thus, the ^18^O-enrichment of biomass resulting from an incubation with H_2_^18^O provides an indicator of a *general* metabolic activity of a cell (Ye et al., 2009) and cannot be assigned to a specific metabolic pathway such as poly-P synthesis. However, when SIP with H_2_^18^O is combined with nanoSIMS, the general and poly-P-specific activity can be assessed separately through separate and spatially resolved measurement on poly-P granules and other cell material offered by nanoSIMS (Langer et al., 2018).

In this study, we combine dual-label SIP (^13^C and ^18^O) and nanoSIMS to investigate the poly-P metabolism in cable bacteria, including the spatial-temporal dynamics of poly-P synthesis and its connection to carbon metabolism. To this end, we amended sediment cores containing an active cable bacteria population with ^18^O-labeled water (targeting both poly-P synthesis and general metabolism) and ^13^C-labeled bicarbonate (targeting inorganic carbon assimilation and thus biomass growth). After 6 and 24 h of incubation, we retrieved individual cable bacterium filaments from three zones in the sediment (oxic, transition, and suboxic) and measured their ^18^O and ^13^C isotope labeling and relative phosphorus content with nanoSIMS.

## Materials and Methods

### Cable Bacteria Culturing

Enrichment cultures with cable bacteria were prepared from natural sediment collected on 27-09-2019 within a creek bed from the Rattekaai Salt Marsh (Netherlands; 51.4391°N, 4.1697°E). At this site, earlier studies have documented the presence of cable bacteria *in situ* (Malkin et al., 2014). After collection in the field, the sediment was brought to the laboratory at Utrecht University, where it was sieved (500 μm mesh size) to remove fauna and large debris, homogenized, and subsequently re-packed into polycarbonate cores (height: 12 cm, inner diameter: 5.2 cm). The sediment cores were submerged in artificial seawater (ASW; salinity of 32, the *in situ* value) and incubated in the dark for several weeks until an active cable bacteria population developed. The overlying seawater was continuously bubbled with air to maintain 100% air saturation, and the temperature (20°C) and salinity were kept constant throughout the incubation. A total of 19 cores were incubated, all prepared from the same batch of sediment.

### Microsensor Depth Profiling

Cable bacteria activity was monitored within the incubated cores using microsensor depth profiling (O_2_, H_2_S, and pH). This so-called geochemical fingerprint provides information about the developmental state and metabolic activity of the cable bacteria population (Nielsen et al., 2010; Risgaard-Petersen et al., 2012; Malkin et al., 2014). The microsensor depth profiles were also used to delineate the oxic, transition, and suboxic zones in the sediment at the time of core sectioning (see Section “Filament Retrieval from the Sediment”).

Microsensors (tip diameters; O_2_: 50 μm, H_2_S: 100 μm, pH: 200 μm) were purchased from Unisense A/S (Denmark), connected to a four-channel Microsensor Multimeter (Unisense), and mounted in a two-dimensional micro-profiling system that enabled stepwise movement of sensors. The SensorTrace PRO software (Unisense) was used to control the vertical movement of the microsensors and record sensor signals. A general-purpose reference electrode (REF201 Red Rod electrode; Radiometer Analytical, Denmark) was used during pH measurements. Calibration of the microsensors was performed as previously described (Malkin et al., 2014).

Cable bacteria population developed in all but one of the incubated sediment cores. Five cores with the largest ΔpH were selected for the SIP experiment. The quantity ΔpH, defined as the difference between the maximum and minimum pH in the oxic and suboxic zone, respectively, provides a good proxy for comparing cable bacteria activity among different populations (Burdorf et al., 2018).

### Stable Isotope Probing

For the SIP experiment, 10 mL of stock solution was prepared by mixing 2 mL of H_2_^18^O (Sigma-Aldrich; ^18^O atom fraction of 0.99) and 8 mL of artificial seawater (ASW) with a natural abundance of ^18^O (^18^O atom fraction of 0.002). Hence, the ^18^O atom fraction of water in the stock solution was 0.2. The ASW contained no Mg^2+^ and Ca^2+^ ions (to avoid precipitation of Mg^13^CO_3_ and Ca^13^CO_3_) and no bicarbonate ions (to avoid ^13^C label dilution). The stock solution was additionally labeled in ^13^C by adding ^13^C-bicarbonate (NaH^13^CO_3_, Sigma-Aldrich; ^13^C atom fraction of 0.99) to a final concentration of 62 mM. This concentration and labeling were chosen because they were successfully applied in previous SIP experiments (Vasquez-Cardenas et al., 2015; Geerlings et al., 2020, 2021).

Dual labeling (H_2_^18^O and H^13^CO_3_^–^) of the sediment cores was done by first inserting a sub-core (inner diameter 1.2 cm, length 5 cm) into each culturing core without disturbing the sediment, and then injecting 500 μL of the labeled stock solution into the sub-core in ten separate and parallel 50 μL injections. To ensure homogeneous spread of the label throughout the sediment, the syringe needle was first inserted to a depth of 5 cm, and then the 50 μL dose of liquid was released while slowly retracting the needle upward. The use of the sub-core ensured that the label was spread within a well-constrained volume. Subsequently, the cores were incubated for 6 h (two replicate cores) and 24 h (two replicate cores), with one additional core chosen as an unlabeled control. Temperature was kept constant at 20°C during all incubations. At the end of the SIP incubation period, the sub-core was carefully pulled out of the sediment core and sectioned based on the redox zonation (see next section). Cable bacteria were retrieved from each sediment section and imaged by scanning electron microscopy (SEM) and NanoSIMS.

During the SIP incubation, each sub-core was overlain with a thin layer of water (∼2 mm) with the same ^18^O and ^13^C labeling as the porewater. Additionally, the cores were placed in a sealed container filled with air and the bottom covered with a thin layer of ASW with the natural abundance of ^18^O and a similar ^13^C labeling as that of the porewater. This setup ensured similar ^13^C labeling of the porewater and the CO_2_ pool in the surrounding atmosphere inside the sealed contained and thus negligible ^13^C label loss from the porewater due to air-water gas exchange. Because the system was stagnant, the loss of ^18^O label from the porewater due exchange with the thin layer of ASW at the bottom of the sealed container was also negligible.

Based on the volume of the sub-core (5.65 mL) and the porosity of sediments from the Rattekaai salt marsh (0.75; L. Burdorf, 2017 thesis, p. 141), the porewater volume in the sub-core was estimated at 4.24 mL. Since 500 μL of the porewater was replaced by the stock solution, the estimated final ^18^O atom fraction of the porewater was 0.025. This is about two-fold greater than the ^18^O-labeling of water used in the SIP experiment by Langer et al. (2018) (S. Langer, personal communication). We assumed that all sub-cores in the replicate cores had the same ^18^O-labeling, as the sub-core dimensions and the volume of the injected stock solution were identical.

### Filament Retrieval From the Sediment

Cable bacterium filaments were retrieved from the sediment matrix under a stereo microscope using fine glass hooks custom-made from Pasteur pipettes. Filaments were retrieved separately from the oxic zone (0-2 mm depth), middle of the suboxic zone (5-10 mm depth), and the oxic-suboxic transition zone, the latter defined here as the zone up to 1 mm below the oxic zone (Supplementary Figure 1). The depth range of these zones were derived from microsensor measurements conducted just before core sectioning. Previous studies showed that in the transition zone, the density (Seitaj et al., 2015) and motility (Bjerg et al., 2016) of cable bacteria are highest. Retrieved filaments were washed several times (> 3) in Milli-Q water (Millipore, Netherlands) to eliminate precipitation of salts, transferred onto polycarbonate filters (pore size 0.2 μm; Isopore, Millipore, Netherlands) that were pre-coated with a 5-10 nm thin gold layer, and air-dried in a desiccator for about 24 h.

### Scanning Electron Microscopy

Filaments on the polycarbonate filters were imaged with a scanning electron microscope (JEOL Neoscope II JCM-6000, Japan) to identify filament segments suitable for NanoSIMS analysis. Imaging was done under a 0.1-0.3 mbar vacuum and a high accelerating voltage (15 kV) using a backscatter electron detector.

### NanoSIMS Analysis

NanoSIMS analysis was performed with the nanoSIMS 50L instrument (Cameca, France) operated at Utrecht University. Fields of view (FOV) selected through SEM imaging were pre-sputtered with Cs^+^ ions until secondary ion yields stabilized. Subsequently, the primary Cs^+^ ion beam (current: 0.5-10 pA, energy: 16 keV, beam size: 130 nm, dwell time: 1-2 ms per pixel) was scanned over the FOV (areas between 10 × 10 μm and 20 × 20 μm in size) while detecting secondary ions ^12^C^14^N^–^, ^13^C^14^N^–^, ^31^P^–^, ^16^O^–^, ^18^O^–^, and ^32^S^–^.

Initial measurements employed a relatively short pre-sputtering interval (10 min) and a low primary ion current (0.5-2 pA), which resulted in relatively low ^18^O^–^ and ^31^P^–^ ion yields during the subsequent analysis conducted with the same current. Thus, during the analysis, the primary ion current was increased to 10 pA to enable quantification of ^18^O labeling in poly-P granules with a desirable precision and within a reasonable time interval (few hours). This increase in the primary ion current resulted, however, in excessive count rates on the electron multiplier used for the detection of ^12^C^14^N^–^ (> 10^5^ cps). To prevent detector aging due to such high count rates, ^12^C^14^N^–^ ions were therefore not detected during these measurements. As a down-side, ^13^C labeling could not be determined for these initial measurements.

Later in the analysis, we noted that the ^18^O^–^ and ^31^P^–^ secondary ion yields were low during the initial measurements because the probed volume was too close to the cell surface, whereas the poly-P granules were present deeper within the cell biovolume. Therefore, we changed the measurement protocol by including much longer pre-sputtering intervals (20-30 min), which allowed us to probe the more inner parts of the cells during the subsequent analysis using low primary ion currents (0.5-2 pA). Specifically, it allowed us to detect all target secondary ions and thus simultaneously determine both ^13^C and ^18^O labeling of the biomass of cable bacteria, including poly-P granules.

NanoSIMS analysis of most samples focused on the variation of the mean isotopic and elemental composition among cells within filaments. In these analyses, the same FOV was imaged multiple times (100-300 frames) and the resulting ion count images were aligned and accumulated. For selected samples, we aimed to obtain additional insight into the 3D distribution of the isotopic and elemental composition within cells. These measurements were therefore conducted with a substantially larger number of frames (up to 7,000) until the sample material was completely sputtered by the primary ion beam.

**FIGURE 1:**
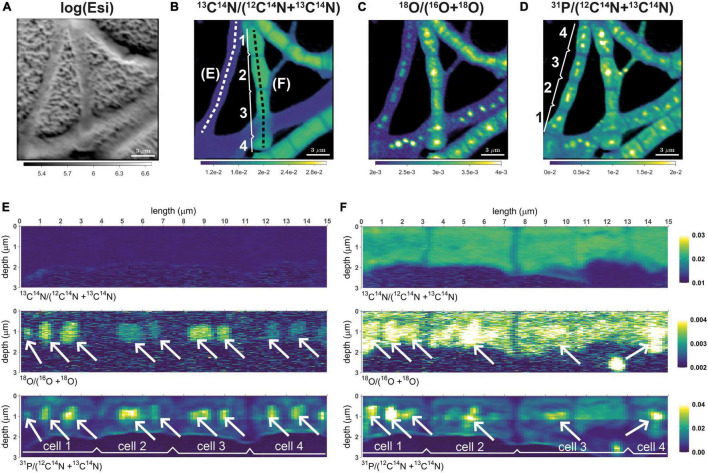
Distributions of ^13^C and ^18^O labeling in cable bacteria. Shown are representative data for filament segments retrieved from the suboxic zone after 6 h of incubation. **(A)** Intensity of secondary electrons (log-transformed). **(B)**^13^C atom fraction, calculated as ^13^C^14^N/(^12^C^14^N + ^13^C^14^N). **(C)**^18^O atom fraction, calculated as ^18^O/(^16^O + ^18^O). **(D)** Relative phosphorus content, approximated as ^31^P/(^12^C^14^N + ^13^C^14^N). **(E,F)** Depth profiles of the ^13^C atom fraction, ^18^O atom fraction, and the relative phosphorus content along profiles spanning across four cells from two filaments. Profiles correspond to filaments that showed no (panel **E**) and some of the highest (panel **F**) carbon assimilation during the incubation. The corresponding profile locations are shown by the white and black dotted lines in panel **(B)**. White arrows point toward selected polyphosphate granules present in the cells. Color scales for panels **(E,F)** are the same. Scale bar is 3 μm.

NanoSIMS data were processed using the Matlab-based software Look@NanoSIMS (Polerecky et al., 2012). After alignment and accumulation of the measured planes, regions of interest (ROIs) corresponding to the poly-P granules were drawn manually using the combined ^12^C^14^N^–^ (or ^13^C^14^N^–^), ^18^O^–^ and ^31^P^–^ ion count images. ROIs were not drawn for cells that appeared damaged. For each ROI, the ROI-specific ^18^O atom fraction was calculated as x(^18^O) = ^18^O^–^/(^16^O^–^ + ^18^O^–^) using the total counts of ^18^O^–^ and ^16^O^–^ accumulated over the ROI pixels. Similarly, the ROI-specific ^13^C atom fraction was calculated as x(^13^C) = ^13^C^14^N^–^/(^12^C^14^N^–^ + ^13^C^14^N^–^) from the total counts of ^12^C^14^N^–^ and ^13^C^14^N^–^ (only if the ^12^C^14^N^–^ ions were detected). Note that when detecting secondary ions from a given poly-P granule, the probed volume partly also included the cytoplasm of a cell in which the poly-P granule was embedded (e.g., “above” or “below” the granule; see Results, Figure 1). Thus, the ^13^C atom fraction determined in the ROI drawn around a poly-P granule represents the ^13^C atom fraction in the surrounding cytoplasm.

To quantify ^13^C and ^18^O labeling, we used excess atom fractions calculated according to x^E^(^13^C) = x(^13^C) – x_i_(^13^C) and x^E^(^18^O) = x(^18^O) – x_i_(^18^O), respectively, where x_i_(^13^C) and x_i_(^18^O) correspond to the atom fractions determined for control cells. Based on these quantities, we classified filaments as ‘inactive’ when x^E^(^13^C) < 0.0006 and x^E^(^18^O) < 0.00025 (the threshold levels correspond to 3 standard deviations of the respective excess atom fractions determined for control filaments), ‘minimally active’ when x^E^(^13^C) ≤ 0.001 (reflecting the lowest 25% of measured filament fragments and 2% of the maximum measured excess ^13^C atom fraction) and x^E^(^18^O) ≤ 0.0004 (which reflects the lowest 10% of measured filament fragments), and ‘active’ when x^E^(^13^C) > 0.001 or x^E^(^18^O) > 0.0004.

To gain insight into the 3D distribution of the isotopic and elemental composition and the position of the poly-P granules within cells, Look@NanoSIMS was additionally used to visualize the depth variation in the nanoSIMS data along a lateral or transversal profile. This analysis was done as previously described (Geerlings et al., 2021).

### Statistical Analysis

Overall, data was obtained for 1887 poly-P granules in 884 cells from 203 filament segments (Table 1). Out of these, 126 poly-P granules in 29 cells from 7 filament segments belong to the control samples. Both the ^18^O and ^13^C atom fractions are available for 704 cells (from 164 filament segments), while data available for the remaining 151 cells (from 32 filament segments) only includes the ^18^O fraction.

**TABLE 1:** Number of poly-P granules, cells and filament fragments measured in this study.

		6h incubation						24h incubation							
		core 1			core 2			core 3			core 4				
	redox zone	oxic	transit	subox	oxic	transit	subox	oxic	transit	subox	oxic	transit	subox	control	total
^18^O atom fraction	# poly-P	147	432	164	121	333	42	64	66	260	101	15	16	126	1887
	# cells	51	166	45	82	243	24	30	33	110	50	10	11	29	884
	# filaments	8	35	8	22	64	6	8	5	25	9	3	3	7	203
^13^C atom fraction	# cells	32	117	45	82	243	24	-	-	101	50	10	-	-	704
	# filaments	4	25	8	22	64	6	-	-	23	9	3	-	-	164
Porewater ^18^O atom fraction		0.0234			0.0164			0.0151			0.0160			0.002	

*Numbers are shown separately for the two replicate cores, three redox zones, two incubation periods, and two isotope labels used. Note that the ^13^C labeling data is available for a subset of filament fragments for which the ^18^O labeling data is available.*

Statistical analysis of this dataset focused on the following aspects: (i) the effect of the redox environment and labeling period on the ^13^C labeling of the cytoplasm and the ^18^O labeling of the poly-P granules, (ii) variation in the ^18^O labeling of the poly-P granules within a cell, within a filament, and among filaments, and (iii) the relationship between the average ^18^O labeling of the poly-P granules and the corresponding ^13^C labeling of the surrounding cytoplasm within each filament fragment.

The first two aspects were assessed by fitting the data with a linear mixed model (separately for the ^13^C and ^18^O data), which was done in R using the package *nlme* (Pinheiro et al., 2021). The analysis considered the hierarchical structure of the data (i.e., nesting of cells within filaments for the ^13^C data, and nesting of poly-P granules within cells, which are further nested within filaments, for the ^18^O data) and the unbalanced experimental design (Table 2). The ‘redox zone’ (oxic, transition, suboxic) and the ‘labeling period’ (6 h, 24 h) were defined as fixed effects, whereas ‘filament’ (when testing the excess ^13^C labeling of cells) and ‘cell’ and ‘filament’ (when testing the ^18^O labeling of poly-P granules) were chosen as random effects in the model. For model selection, a step-up approach was used, which starts with a reference model that contains all fixed components and their interactions. This so-called “beyond optimal model” was then used to find the best variance structure and random structure (Zuur et al., 2009). Using the output of the linear mixed model for the poly-P-specific ^18^O data, we calculated the percentage of variance in the data explained by differences among filaments, among cells within the same filament, and differences within the same cell. Similarly, we calculated the percentage of variance explained by differences among filaments and among cells within the same filament for the cell-specific ^13^C data. The third aspect was assessed by calculating the Kendall rank correlation coefficient (τ), which is a robust parameter used for testing correlations in non-normally distributed data (Dalgaard, 2013). Details of the statistical analysis are provided in the Supplementary Methods.

**TABLE 2:** Description of the different variables used in the linear mixed models.

Name	Description	Type of variable	Levels in model 1	Levels in model 2
frac_18O	^18^O atom fraction in the ROI defined as a poly-P granule	continuous response	1761	-
frac_13C	^13^C atom fraction of the cell cytoplasm	continuous response	-	704
Cell	unique label for each cell	categorical random explanatory	855	
Filament	unique label for each individual filament fragment	categorical random explanatory	196	164
Core	the core from which the measurement was extracted	categorical random explanatory	4	4
Zone	redox zonation	categorical fixed explanatory	3	3
labeling period	labeling period of the polyphosphate granule	categorical fixed explanatory	2	2

## Results

### Patterns in ^13^C and ^18^O Labeling of Cable Bacterium Filaments

Patterns shown by the ^13^C data in the present study are similar to those observed previously (Geerlings et al., 2020, 2021). ^13^C labeling was mainly restricted to filaments from the transition and suboxic zone, whereas filaments from the oxic zone displayed no, or only very low, ^13^C labeling (SI Appendix, Supplementary Figure 2). Furthermore, the ^13^C labeling was highly variable among filaments from the transition and suboxic zone, but highly similar when compared among cells within the same filament (Figure 1 and Supplementary Figure 2).

In contrast to the low intra-filament variability in ^13^C labeling, there was a clear variation in the ^18^O labeling of poly-P granules among cells from the same filament (Figure 1). However, adjacent cells displayed a similar pattern when compared with respect to the size, position, and number of granules per cell, which is congruent with earlier observations on poly-P granules in cable bacteria (Geerlings et al., 2019).

**FIGURE 2:**
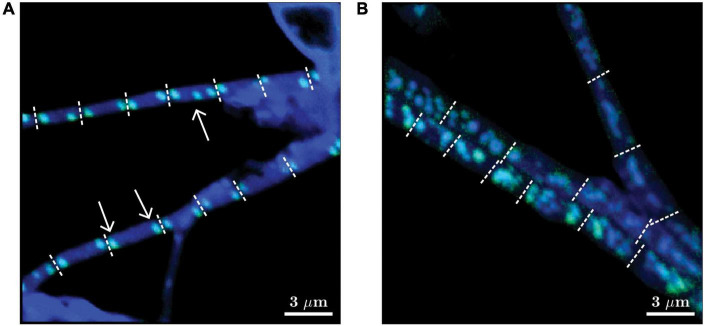
Representative images of two morphotypes of cable bacteria observed in this study. Images are shown as overlays of the ^18^O atom fraction (in green) and ^31^P ion counts (in blue). **(A)** “Thin” filaments (diameter 0.5-1 μm) mostly contained two poly-P granules at the poles of each cell, but cells containing three poly-P granules were occasionally also observed (see white arrows). **(B)** “Thicker” filaments (diameter > 1 μm) contained multiple poly-P granules per cell. Filaments shown in panels **(A,B)** were retrieved from the transition zone after 24 h of labeling and from the suboxic zone after 6 h of labeling, respectively. Dotted white lines depict cell junctions. Scaling for the green and blue colors was optimized independently for each image to enhance the visibility of the poly-P granules. Scale bar is 3 μm. The original images of the ^18^O atom fraction and ^31^P ion counts are shown in Supplementary Figure 3.

Depending on the diameter of the filament and the intracellular spatial organization of the poly-P granules, two different morphotypes of cable bacteria were distinguished: (i) “thin” filaments (diameter ∼0.5-1 μm), which mostly contained two similarly-sized (diameter ∼100-300 nm) poly-P granules per cell, one at each cell pole (Figure 2A), and (ii) “thicker” filaments (diameter above 1 μm), which contained variable spatial organizations of the poly-P granules within cells. Some cells in the thicker filaments contained many small poly-P granules, while others contained only a few larger ones (Figure 2B). It is unknown whether the different morphotypes represent different species of cable bacteria or they are different manifestations of the same species in a different stage of the life cycle.

**FIGURE 3:**
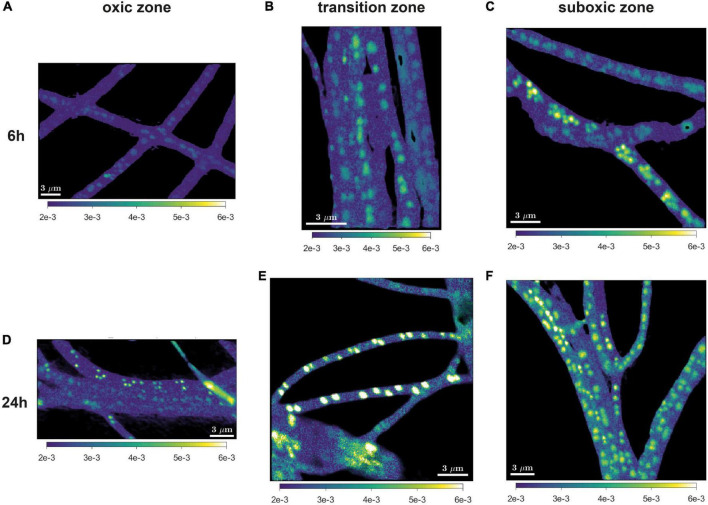
Representative images of the ^18^O atom fraction in cable bacteria. Filaments were retrieved after 6 h **(A–C)** and 24 h **(D–F)** of incubation and retrieved from the oxic **(A,D)**, transition **(B,E)**, and suboxic **(C,F)** zone. All scale bars are 3 μm. Color scale in all images is the same (0.002-0.006).

**FIGURE 4:**
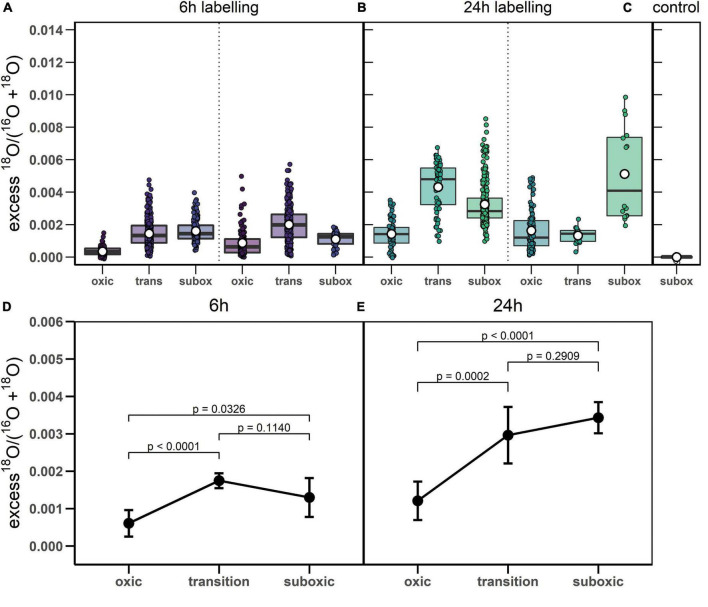
^18^O labeling of polyphosphate granules in cable bacteria. **(A-C)** Boxplots show excess ^18^O atom fractions in individual poly-P granules, separately for the 6 h and 24 h labeling periods and for the control cells. Dotted lines separate data obtained from replicate sediment cores. Shown are the corresponding mean (white open dot), median (black line) and upper and lower quantiles of the excess ^18^O atom fraction. Note that grouping of poly-P granules within cells and within filaments is not indicated in the graphs. **(D,E)** Results of the linear mixed model used for fitting the variance in the excess ^18^O atom fractions in poly-P granules. Results are shown separately for the 6 h and 24 h labeling period. Black circles and error-bars depict the best estimates and the lower and upper confidence limits (95%) of the mean value for each zone and labeling period (exact values provided in Supplementary Table 1). The p-values indicate the significance of differences between zones (summarized in Supplementary Table 2). Raw data is provided as Supplementary Dataset 1, the step-by-step build-up and outcomes of the linear mixed model are available in Supplementary Methods.

Both the ^13^C labeling of the cytoplasm and the ^18^O labeling of the poly-P granules were highly variable when compared among different filaments (Figures 1, 3, 4, Table 3 and Supplementary Figure 2). Based on the combined ^13^C and ^18^O data, we divided the filaments into four classes (see Methods for details). Class 1 includes filaments considered as metabolically inactive or minimally active during the labeling period. This class contained 4.3% (7/164) of filaments (1 inactive and 6 minimally active), all originating from sediment cores incubated for 6 h. Note that this number does not include filaments without detectable poly-P granules at the end of the nanoSIMS analysis. Class 2 includes filaments that showed ^13^C labeling of the cytoplasm but no, or only minimal, ^18^O labeling of the poly-P granules. This pattern was observed in 3.7% (6/164) of filament fragments, all from the 6 h incubation. Class 3 includes filaments that showed no, or only minimal, ^13^C labeling but contained poly-P granules with a significant ^18^O labeling (Figure 1E). This pattern, observed in 23% (38/164) of filament fragments, indicates that the filaments did not grow during the SIP incubation but were still active with respect to poly-P metabolism (see Discussion). The highest proportion of filaments (113/164, i.e., 69%) was assigned to class 4, which includes filaments with high labeling in both ^13^C and ^18^O. For these filaments, ^18^O labeling of the poly-P granules was significantly greater than that of the cytoplasm (Figure 1F). This labeling pattern indicates that these filaments were simultaneously active with respect to growth and poly-P metabolism. Overall, around 78% of filaments from the transition and suboxic zone were actively growing during the 6 h or 24 h incubation period, and around 92% of all measured filaments (from all redox zones) displayed poly-P activity (Table 3).

**TABLE 3:** Number of cable bacterium filament fragments with a specific ^13^C labeling of their cytoplasm and ^18^O labeling of their poly-P granules.

	6h			24h		
	Oxic	transition	suboxic	oxic	transition	suboxic
Minimally active	5	2	0	0	0	0
Only ^13^C labeled	4	1	1	0	0	0
Only ^18^O labeled	8	20	6	3	0	1
Both ^13^C & ^18^O labeled	9	66	7	6	3	22
Total	26	89	14	9	3	23
	129			35		

*Numbers are shown separately for each incubation period and redox zone. Details of the classification are explained in Methods. Images of filaments from each class are shown in Supplementary Figure 2.*

### Effect of Redox Zonation and Labeling Period

**FIGURE 5:**
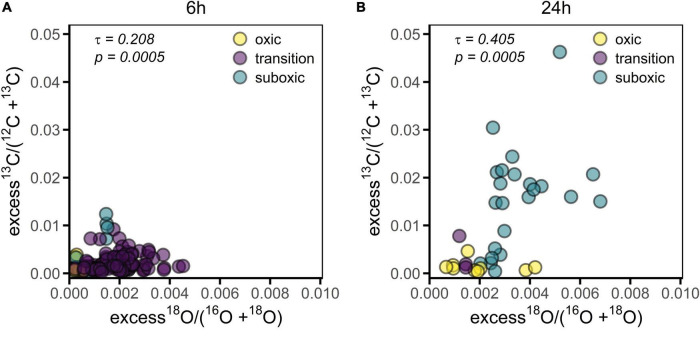
^13^C labeling of the cell cytoplasm versus ^18^O labeling of the poly-P granules in cable bacteria. Each data point represents a mean value for an individual filament segment. **(A)** Values obtained for filaments incubated for 6 h (*n* = 129). **(B)** Values obtained for filaments incubated for 24 h (*n* = 35). Colors differentiate among redox zones. Kendall’s correlation coefficient (τ) and the corresponding *p*-value are also shown.

^18^O labeling of poly-P granules was observed in filaments from all redox zones and varied greatly among filaments (Figures 3–5). Despite this high variability, statistical analysis revealed that the labeling values in filaments from the oxic zone were, on average, significantly lower than those from the transition and suboxic zones. In contrast, the transition and suboxic zones showed no significant differences (Figures 4D,E and Supplementary Table 2). This pattern was detected for both labeling periods (6 h and 24 h), although it was more pronounced for the longer period. Furthermore, a comparison made separately for each redox zone revealed a significantly higher ^18^O labeling of poly-P granules in filaments incubated for 24 h compared to 6 h (Figures 4D,E and Supplementary Table 1).

### Variation Within Cells, Within Filaments, and Among Filaments

Statistical analysis further revealed that most of the variability in the ^18^O labeling of poly-P granules (about 52-88%) was explained by differences among filaments, with the percentage being highest in the oxic zone (consistent for each labeling period) and higher for the shorter labeling period compared to the longer one (consistent for each redox zone) (Table 4). The percentage of variance explained by differences among cells within filaments was relatively minor (about 5-8%) and similar for both labeling periods and all redox zones. Finally, a sizeable fraction of the variance (about 4-44%) was unexplained, i.e., was due to unknown differences within cells, with the percentage being lowest in the oxic zone (consistently for each labeling period) and higher for the longer labeling period compared to the shorter one (consistently for each redox zone) (Table 4). For the ^13^C labeling of cell cytoplasm, most of the variability among cells (86-99%) was explained by differences among filaments and only minor differences (1-14%) were observed among cells within the same filament (Supplementary Table 3). Thus, similar to previously observed patterns (Geerlings et al., 2020, 2021), there was only a minimal variability in ^13^C labeling among cells within a given filament from a given redox zone. Additionally, variability among cells within filaments was lower for the ^13^C labeling of the cell cytoplasm compared to the ^18^O labeling of poly-P granules.

**TABLE 4:** Structure of the variance in the ^18^O labeling of poly-P granules and ^13^C labeling of the cytoplasm in the measured cable bacteria.

		Excess ^18^O/O			% of variance explained by differences			Excess ^13^C/C		% of variance explained by differences	
Labeling period	Redox zone	σ^2^	ICC_cell_	ICC_filament_	among filaments	among cells within filaments	within cells	σ^2^	ICC_filament_	among filaments	among cells within filaments
	oxic	0.00021	0.96	0.8836	88.4%	7.6%	4.0%	0.00018	0.992	99.2%	0.8%
6h	transition	0.00049	0.8133	0.7485	74.9%	6.5%	18.7%	0.00057	0.956	95.6%	4.4%
	suboxic	0.00043	0.8477	0.7802	78.0%	6.8%	15.2%	0.00065	0.965	96.5%	3.5%
	oxic	0.00047	0.8274	0.7615	76.2%	6.6%	17.3%	0.00039	0.96	96.0%	4.0%
24h	transition	0.00062	0.7248	0.6671	66.7%	5.8%	27.5%	0.00060	0.929	92.9%	7.1%
	suboxic	0.00090	0.5592	0.5146	51.5%	4.5%	44.1%	0.00069	0.864	86.4%	13.6%

*The values of σ^2^ and the intraclass correlation coefficient (ICC) were determined by fitting the data with linear mixed models (see Supplementary Methods for details). The ICC values indicate that the largest portions of the variance can be explained by differences among filaments (52-88% for ^18^O labeling and 86-99% for ^13^C labeling) and there is little variation among cells within a filament (5-8% for ^18^O labeling and 1-14% for ^13^C labeling). Since for most cells the ^18^O labeling was determined in more than one poly-P granule, there is also a variation within a cell, i.e., an unexplained variance (4-44%).*

### Correlation Between ^18^O and ^13^C Labeling of Cable Bacterium Filament Fragments

The filament-averaged ^18^O atom fractions of poly-P granules and the corresponding averaged ^13^C atom fractions of the cytoplasm were determined for the same filament fragment for each of the labeling periods. Correlation analysis showed that these quantities were significantly correlated for each of the labeling periods (*p* = 0.0005, Figure 5). However, this correlation was largely a collinearity effect caused by the dependence of both variables on the redox environment. In all cases, however, the predictive power of the correlation was poor, as indicated by the low value of the Kendall rank correlation coefficient (τ = 0.208 and 0.405 for the 6 h and 24 h labeling period, respectively).

## Discussion

Overall, detailed nanoSIMS imaging revealed that poly-P granules are a prominent feature of cable bacteria that takes up a large fraction of the cell biovolume (Figure 1). Poly-P granules were detected in nearly all cable bacteria filaments (Supplementary Figure 2), and nearly all detected poly-P granules were labeled by ^18^O (Figure 4 and Table 3). Significant label incorporation was observed after a time interval (6 h and 24 h) that is shorter or comparable to the doubling time of cable bacteria cells in laboratory conditions (∼20 h; Schauer et al., 2014). Additionally, the ^18^O labeling of poly-P granules was highly variable and significantly influenced by the duration of the labeling interval and redox environment. Based on these patterns, we discuss possible factors that control the poly-P metabolism in cable bacteria and suggest a possible role played by poly-P in their life cycle.

### Investigating Polyphosphate Metabolism Using ^18^O-Labeled Water

Before we interpret our data, we briefly review possible pathways through which ^18^O derived from H_2_^18^O can enter poly-P granules.

**FIGURE 6:**
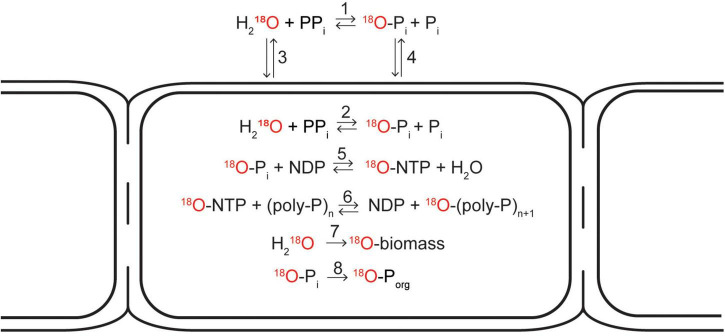
Schematic diagram depicting possible pathways how the ^18^O atom from an ^18^O-labeled water molecule can end up in the cellular inorganic polyphosphate pool, cell biomass, and the organic phosphate pool. ^18^O exchange between ^18^O-labeled water and (ortho)phosphate (P_i_) occurs when inorganic pyrophosphate (PP_i_) is hydrolyzed. This process is catalyzed by enzymes and can occur both outside (1) and inside the cell (2). When occuring inside the cell, ^18^O-labeled water is first transported into the cell via diffusion (3). When occuring outside the cell, the ^18^O-labeled phosphate is transported inside the cell either actively (via membrane-bound transport proteins) or passively via diffusion (4). As soon as the ^18^O-labeled phosphate is in the cytoplasm, it can be incorporated into a nucleoside triphosphate molecule (e.g., ATP) if energy is available (5), from which it can be added onto a polyphosphate chain (6). ^18^O atoms from ^18^O-labeled water can also be incorporated into cell biomass during cell growth (7). Additionally, ^18^O-labeled phosphate can be incorporated into cellular organic phosphate pools such as DNA, RNA, or phospholipids (8). Modified from Blake et al. (2005).

The first step involves ^18^O exchange between water and the inorganic (ortho)phosphate (P_i_) pool, which occurs when inorganic pyrophosphate (PP_i_) is hydrolyzed. This process can occur both inside and outside of a cell (Figure 6, reactions 1 and 2). The PP_i_ required in this step is produced in a metabolically active cell by many different processes, such as hydrolysis of ATP into AMP or breakdown of large biomolecules. At temperatures below 80°C, abiotic phosphoryl-transfer reactions such as PP_i_ hydrolysis are very slow but are significantly accelerated by enzymes (Lassila et al., 2011). Therefore, in natural systems, the ^18^O signature of the P_i_ pool (δ^18^O_*p*_) is dominated by enzyme-mediated ^18^O exchange with water (Blake et al., 2001, 2005). When this process occurs intracellularly, H_2_^18^O first enters the cell via diffusion and the ^18^O exchange is then catalyzed by the enzyme pyrophosphatase (PPase). In contrast, the ^18^O exchange outside of a cell occurs, e.g., due to the activity of microorganisms that use extracellular phosphatases (e.g., phosphomonoesterases) as a strategy to acquire P_i_ from organic compounds (Liang and Blake, 2006; Von Sperber et al., 2014), and the ^18^O-labeled P_i_ then enters the cell via diffusion. We assume that in both cases the intracellular ^18^O isotope equilibrium between H_2_O and P_i_ is established within a few hours (Blake et al., 2005; Chang and Blake, 2015).

Once the intracellular P_i_ pool is labeled with ^18^O, the label can enter the poly-P granule via poly-P synthesis. This process is catalyzed by two families of poly-P kinases, PPK1 and PPK2 (Rao et al., 2009), and involves incorporation of P_i_ into a nucleoside triphosphate (NTP, such as ATP or GTP) followed by a transfer of P_i_ from NTP to a poly-P chain (Figure 6, reactions 3 and 4). As these reactions consume NTP, poly-P synthesis hence requires the investment of metabolic energy. Cable bacteria carry the genes necessary to produce enzymes from both of these families (Kjeldsen et al., 2019).

Poly-P breakdown was suggested as another mechanism how ^18^O could enter the poly-P granule (Langer et al., 2018). Poly-P breakdown can proceed via hydrolysis, which is catalyzed by an exopolyphosphatase (PPX) and leads to the cleavage of the terminal P_i_ from the poly-P chain (Kornberg et al., 1999). However, evidence suggests that this pathway proceeds via a concerted mechanism with a single transition state where the nucleophilic water attacks the P-atom at the end of the poly-P chain. As a result, the O-atom from the water molecule eventually ends up in the P_i_ residue *leaving* the chain, and so there is no labeling of the poly-P (Lassila et al., 2011). Another possible pathway of poly-P breakdown involves phosphorylation of nucleoside monophosphates (AMP or GMP) or diphosphates (ADP and GDP) (Ishige et al., 2002; Nocek et al., 2008, 2018). This process is aided by PPK2-type enzymes, and most likely also proceeds via a concerted mechanisms with a single “loose” transition state (Nocek et al., 2018) where the O-atom from the water molecule ends up in the NDP or NTP molecule rather than in the terminal P_i_ group on the remaining poly-P chain. Thus, poly-P breakdown in the presence of H_2_^18^O is unlikely to increase significantly the ^18^O atom fraction of an unlabeled poly-P granule (a minor effect, expected to occur due to kinetic isotope fractionation, is neglected in this study).

Poly-P breakdown could, however, decrease the ^18^O atom fraction of a previously ^18^O-labeled poly-P granule, if the ^18^O-labeled P_i_ groups added to the granule during poly-P synthesis were removed preferentially during the subsequent phase of poly-P breakdown. This could occur, for instance, if the newly synthesized poly-P was incorporated into a specific location within a granule, such as the outer surface, and the breakdown involved poly-P from the same location. Our detailed 3D nanoSIMS analysis revealed, however, that there is no significant variability in ^18^O labeling within a poly-P granule (Figure 1), indicating that, on the scale of hours, the poly-P pool within a granule becomes well mixed. This mixing is likely the result of structural organization of a granule where the negatively charged poly-P chains are connected to one another via ion bridges involving divalent cations which causes the tertiary structure to be rearranged and the ^18^O label to become well-mixed. This hypothesis is supported by the recently documented association between Ca^2+^ and Mg^2+^ ions and poly-P granules in cable bacteria (Geerlings et al., 2019) and a modeling study on the conformation and organization of poly-P molecules and Ca^2+^ ions (Müller et al., 2019). Thus, due to the well-mixed nature of the poly-P granule, poly-P breakdown has no significant effect on the ^18^O atom fraction of an ^18^O-labeled poly-P granule.

Based on these arguments, we conclude that in the presence of H_2_^18^O and an ^18^O-labeled P_i_ pool, the ^18^O atom fraction of a poly-P granule can only increase (during poly-P synthesis) or stay constant (during poly-P breakdown). Thus, a significant enrichment of poly-P granules in ^18^O indicates that poly-P synthesis occurred *at some point* during the SIP incubation with H_2_^18^O, but it may be insufficient to provide insights into the actual rate of poly-P synthesis or the cycling between poly-P synthesis and breakdown.

### Polyphosphate Turnover in Cable Bacteria

Our poly-P-specific ^18^O data indicate that on the time scale of 24 h, cable bacteria cells turn over a significant amount of poly-P. This conclusion derives from the comparison of ^18^O data between the 6 h and 24 h labeling intervals. Specifically, if poly-P synthesis and incorporation into existing poly-P granules at a constant rate were the only processes during the incubation, the expected excess ^18^O atom fraction of poly-P granules after 24 h of incubation would be, on average, around 5 to 7-fold greater than after 6 h of incubation (Supplementary Model). This estimate considers that, for the incubation temperature of 22°C, it takes about 27 h for the ^18^O exchange between the P_i_ pool and H_2_^18^O to reach an equilibrium (Blake et al., 2005). However, the excess ^18^O atom fractions of poly-P granules only increased, on average, by a factor of 1.8-2.7 between the 6 h and 24 h incubation periods (Figure 4 and Supplementary Table 1). When combined with the ^18^O labeling asymmetry between poly-P synthesis and breakdown proposed above, this discrepancy suggests that poly-P breakdown occurred during a significant fraction of the 24 h labeling interval. Present data is, however, insufficient to constrain the rates of poly-P synthesis and breakdown or the number of turnover cycles. Research on shorter time scales (∼1 h) is required to gain more insight into the dynamics of poly-P turnover in cable bacteria. Since SIP using ^18^O-labeled water requires an exchange between the P_i_ pool and H_2_^18^O to produce ^18^O-labeled P_i_, research on shorter time scales may be hampered and it is therefore advised to directly add labeled ^18^O-labeled phosphate. This can be prepared either using a minimal abiotic system with commercially available PPase (Blake et al., 2005) or by the hydrolysis of PCl_5_ using H_2_^18^O (Versaw and Metzenberg, 1996).

### Polyphosphate Metabolism Is Governed by Redox Zonation

Poly-P metabolism in cable bacteria is governed by the redox environment, with higher levels of synthesis in the suboxic (and transition) zone and lower levels of synthesis, or higher levels of breakdown, in the oxic zone. This conclusion is supported by the statistical analysis of the poly-P-specific ^18^O labeling, which revealed, consistently for both labeling intervals (6 h and 24 h), a significantly lower mean value in the oxic zone compared to the transition and suboxic zones and no significant differences between the transition and suboxic zones (Figures 4C-D and Supplementary Table 2).

The dependence of poly-P metabolism on the redox environment, as observed in the present study for cable bacteria, seems to follow an opposite pattern to that observed for other poly-P accumulating microorganisms. For most of these organisms, phosphate is stored as poly-P under aerobic conditions, whereas poly-P is broken down under anaerobic conditions (He and Mcmahon, 2011; Saia et al., 2021). This pattern was observed, e.g., for the giant sulfur-oxidizing bacteria *Thiomargarita namibiensis* (Schulz and Schulz, 2005), for the large filamentous sulfide oxidizing bacteria *Beggiatoa spp*. (Brock and Schulz-Vogt, 2011), or when investigating the phosphorus cycling in wetland sediments from South Gippsland (Southern Australia) (Khoshmanesh et al., 1999, 2002).

This apparent inconsistency between cable bacteria and other poly-P accumulating organisms can be explained by considering the peculiar energy metabolism of cable bacteria facilitated by long-distance electron transport along the filament. Although poly-P appears to be synthesized predominantly by cells residing in the anoxic sediment, these cells can only gain energy if part of the filament is connected to oxygen (Geerlings et al., 2020). Thus, the energy metabolism of the poly-P synthesizing cable bacteria cells is *de facto* aerobic.

Another theoretical possibility could be anoxic poly-P synthesis. Based on genomic data, cable bacteria have a metabolic potential for sulfur disproportionation (Kjeldsen et al., 2019), which could be a mechanism for gaining energy required for poly-P synthesis when a filament is disconnected from oxygen. However, the function of cells in their native habitat often cannot be reliably predicted from genomic data (Hatzenpichler et al., 2020). More direct methods need to be employed to test whether poly-P synthesis in cable bacteria is coupled to anaerobic energy metabolism such as sulfur disproportionation.

Our measurements revealed significant ^18^O labeling of poly-P granules in filaments retrieved from the oxic zone (Figures 3, 4), indicating that these cells must have synthesized poly-P at some point during the SIP incubation. Because this process requires energy, preferentially in the form of ATP (Kornberg et al., 1999; Nocek et al., 2008; Rao et al., 2009; Wang et al., 2018), the cells from the oxic zone therefore must have been able to generate energy at some time during the incubation. However, these cells do not possess any known terminal oxidases (Kjeldsen et al., 2019) and thus cannot generate energy from oxygen reduction, as supported by the observation that cells in the oxic zone do not grow (Geerlings et al., 2020, 2021; this study, Figure 5).

A possible explanation for the observed ^18^O labeling of poly-P in cells from the oxic zone is that these cells generated energy via endogenous catabolism. This process is a stress response often observed in microorganisms entering a non-growing stage, where energy is generated from the breakdown of large biomolecules (e.g., DNA, RNA, ribosomes, phospholipids) (Bergkessel et al., 2016). For example, in stressed *E. coli* cells, this stringent response redirects cellular phosphorus toward synthesis of poly-P, which is essential for adaptation to various stresses (including oxidative stress) and survival during periods of no growth (Rao and Kornberg, 1996; Ault-Riché et al., 1998; Rao et al., 1998). For cable bacteria, endogenous catabolism could theoretically be directed toward poly-P synthesis. The ^18^O labeling of poly-P in the cells from the oxic zone could then be explained by assuming that the energy released by the endogenous catabolism is directed toward the synthesis of ATP, which is then used to “propagate” the ^18^O-labeled P_i_ toward poly-P (Figure 6; reactions 5 & 6). Yet why direct energy from endogenous catabolism to poly-P rather than using the energy directly for maintenance and stress release?

An alternative, and perhaps more likely, explanation for the observed ^18^O labeling pattern of poly-P is that the cells retrieved from the oxic zone spent part of the SIP incubation in the suboxic zone, where they synthesized poly-P and thus increased the ^18^O labeling of the poly-P granules, and then migrated to the oxic zone before the end of the incubation. This hypothesis is consistent with the gliding motility of cable bacteria (Bjerg et al., 2016), which is most prominent at the oxic-suboxic boundary, where cells move transiently in and out of the oxic zone such that cell abundance in the oxic zone remains relatively constant (Scilipoti et al., 2021; Yin et al., 2021). It is also consistent with their ability to quickly switch between sulfide oxidation and oxygen reduction depending on the surrounding redox conditions (Geerlings et al., 2020). This hypothesis suggests that the average ^18^O labeling of poly-P granules in cells from the oxic, transition, and suboxic zone reflects the average residence time of the cells in each zone.

### Variability in ^18^O Labeling of Polyphosphate Granules

For each combination of the labeling interval and redox zone, ^18^O atom fractions of poly-P granules showed considerable variability among granules (Figures 4A,B). Most of this variability is explained by differences among filaments in combination with differences among granules within the same cell rather than by differences among cells within the same filament. Here, we discuss possible reasons for these patterns.

#### Variability Among Filaments

For each redox zone and labeling period, variability among filaments explained the largest part of the overall variability in the ^18^O labeling of the poly-P granules (52-88%) and the ^13^C labeling of the cytoplasm (86-99%) (Table 4). The latter result is consistent with previous research, which showed large differences in growth rates among filaments (Geerlings et al., 2020) and synchronized growth and cell division over millimeter length scales (Geerlings et al., 2021).

#### Variability Among Cells of the Same Filament

Consistently for each redox zone, the within-filament variability explained only a small part (5-8%) of variability in the ^18^O labeling of poly-P granules (Table 4). Previously, small within-filament variability was observed for ^13^C labeling of cable bacteria incubated with ^13^C-CO_2_. Specifically for filaments retrieved from the suboxic zone, this pattern was attributed to a synchronized growth and division of cells within a filament (Geerlings et al., 2021). Although synchronicity of poly-P synthesis within a filament is a plausible explanation for the ^18^O labeling patterns observed in the present study, we have not followed individual filaments along stretches containing more than about 10 cells, and hence we cannot draw firm conclusions. On the one hand, we observed that if poly-P granules were ^18^O-labeled in one cell, they were also labeled, to a similar degree, in the neighboring cells (e.g., Figures 3, 4 and Supplementary Figure 2), suggesting that poly-P synthesis is not independent among cells within a filament. On the other hand, differences among cells within a filament were greater for the ^18^O labeling compared to the ^13^C labeling (Table 4), indicating that ^18^O labeling of poly-P granules is influenced by additional factors compared to those controlling ^13^C labeling of cells (see next section). The small within-filament and large between-filament variability observed in the ^18^O labeling of poly-P granules fits the “oxygen pacemaker hypothesis” that states that contact with oxygen serves as a “pacemaker” for long-distance electron transport and energy conservation (Geerlings et al., 2021). Access to oxygen thus determines when the sulfide-oxidizing cells within a filament have the capacity for both poly-P synthesis and growth. However, more research is required to assess the degree of synchronicity of poly-P metabolism among cells within a cable bacterium filament.

#### Variability Within Cells

A relatively large part of variability in the ^18^O labeling of poly-P granules (4-44%) was explained by differences within cells (Table 4). This pattern could be due (1) to methodological artifacts, (2) to differences in the rate at which poly-P is synthesized and incorporated into individual granules, (3) to differences in the periods and timing over which poly-P is synthesized between granules (at the same rate), and (4) to differences in the initial size of poly-P granules. We now discuss each of these factors in more detail.

The analytical procedure used to quantify the granule-specific ^18^O atom fraction could be a possible source of within-cell variability among poly-P granules. We collected ion counts for each field of view over hundreds of planes, which “dissected” the imaged cells across a depth interval that included poly-P granules as well as the cytoplasm above and/or below the granules. This approach provided quantitative information about the ^18^O labeling of the poly-P granule and the corresponding ^13^C labeling of the surrounding cytoplasm in one image stack. Because the ^18^O labeling of the cytoplasm is lower compared to the poly-P granule (Figures 1E,F), the poly-P-specific ^18^O atom fraction calculated from the ^18^O^–^ and ^16^O^–^ ion counts accumulated across all planes in the image stack is lower than it would be if the planes corresponding to the cytoplasm were excluded from the analysis. However, this effect is negligible because the ^16^O^–^ ion counts detected from the poly-P granules were more than ∼50-fold higher than those from the cytoplasm (∼5-fold higher count rates, and > 10-fold greater number of planes).

Differences in the intrinsic rate at which poly-P is synthesized will obviously generate differences in ^18^O labeling among granules. However, it is difficult to envision how the rate of poly-P synthesis can vary within a single cell, as one expects the concentration of both substrates (e.g., P_i_) and catalysts (poly-P kinases) to be homogeneous within the cytoplasm of a single cell. Therefore, a more important factor could be the timing of poly-P synthesis and breakdown in individual poly-P granules in combination with the poly-P content of granules at the beginning of the labeling period (Polerecky et al., 2021). As discussed above, substantial poly-P turnover likely occurred during our incubations, potentially involving multiple cycles of synthesis and breakdown (Supplementary Model). Labeling differences among individual granules can arise if poly-P turnover cycles are not synchronized among granules. This can happen, for example, due to relative delays between cycle onsets or differences in cycle durations. These differences can be amplified by the progressive ^18^O labeling of the P_i_ pool within the first hours of the incubation (poly-P granules formed early will hence show lower labeling levels).

Yet, even when the ^18^O incorporation rate is similar and the labeling period is synchronized and identical among granules, one can expect variation, as differences in the ^18^O labeling of poly-P granules can also arise due to a different granule size at the beginning of the labeling period. For example, if ^18^O-labeled poly-P is incorporated into two differently sized granules and the rate of incorporation is the same for both granules, the smaller granule will have a greater ^18^O atom fraction at the end of the incubation than the larger one (Polerecky et al., 2021). This is because the incorporated ^18^O is mixed across the whole granule, thus providing lower labeling levels for larger granules.

Observation of the different morphotypes (Figures 1, 2 and Supplementary Figure 2) reveals less variation between the two similarly-sized poly-P granules at the cell poles of the “thin” morphotype whereas within-cell variation is greater in cells of the “thicker” morphotype with multiple poly-P granules in the cell. The observed within-cell differences are thus most likely the result of a combination of initial starting sizes of the granules and relative delays between cycle onsets or cycle durations.

### Carbon and Polyphosphate Metabolisms Are Not Coupled

Although both ^13^C labeling of the cell cytoplasm and ^18^O labeling of poly-P granules are, on average, lower in the oxic zone compared to the transition and suboxic zones, the ^13^C labeling does not predict ^18^O labeling for individual cells (Figure 5). Thus, cell growth and poly-P synthesis are linked on the population level due to their common dependence on the redox environment. We cannot exclude that a connection between active growth and poly-P synthesis also exists on the filament and cell level (e.g., in the suboxic zone). However, we observed a sizeable fraction (27/129 or ∼21%) of filaments from the transition and suboxic zones that showed no, or very little, ^13^C labeling of the cytoplasm but clear ^18^O labeling of the poly-P granules (Figure 1E, Table 3 and Supplementary Figure 2), which shows that, on the filament and cell level, growth and poly-P synthesis in cable bacteria can operate independently of each other.

A similar large heterogeneity in growth among filaments from the suboxic zone was observed previously in SIP experiments using DIC labeled with ^13^C (Geerlings et al., 2020, 2021) or ^14^C (Kjeldsen et al., 2019), which also found no significant growth in about 30-50% of filaments from the suboxic zone after 24 h of incubation. In previous studies, filaments displaying no significant growth were considered as inactive, and it was hypothesized that these filaments had no contact with oxygen during the incubation interval and thus could not generate ATP.

**FIGURE 7:**
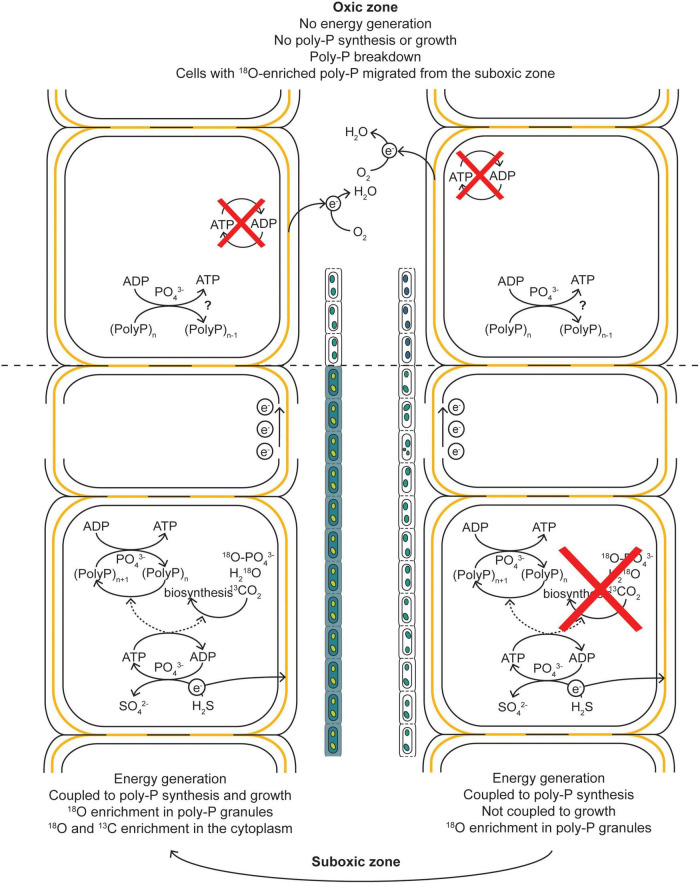
Schematic diagram of our hypothesis explaining the observed ^13^C and ^18^O labeling patterns in cable bacteria. When a filament has access to O_2_, the cells performing the sulfide-oxidizing half-reaction can generate energy in the form of ATP. During a period of growth arrest, this energy is not directed toward biosynthesis and growth but toward the build-up of poly-P granules, resulting in an ^18^O enrichment of the poly-P granules but no ^13^C and ^18^O enrichment of the cytoplasm. During a period of growth, the energy is directed toward both growth and poly-P synthesis, resulting in a ^13^C and ^18^O enrichment of the cytoplasm and ^18^O enrichment of the poly-P granules. The electrons generated via the sulfide-oxidizing half-reactions are transported toward the cells in the oxic zone via the electron-conducting fibers in the shared periplasm of a filament. Within the oxic zone, the electrons are used to reduce O_2_, but this process does not lead to energy generation and thus cannot lead to an ^18^O enrichment in the poly-P granules. That such enrichment is observed in cells from the oxic zone is explained by migration of the cells from the suboxic into the oxic zone during the incubation. Before this migration, the cells entered a non-growing stage of the cell cycle and “prepared” for a survival in the oxic zone. Part of this preparation included the build-up of poly-P granules. Within the oxic zone, the poly-P can be broken down.

Our present results refine this interpretation, because most of the filaments that showed no activity in terms of growth were still actively synthesizing poly-P. Because poly-P synthesis requires energy, most likely in the form of ATP, these filaments must have had the capacity for energy generation, and were therefore most likely connected to oxygen, at least during part of the labeling interval. However, instead of directing this energy toward growth and reproduction, these filaments remained in a state of growth arrest and directed the energy, or at least part of it, toward poly-P synthesis (Figure 7). This way, cable bacteria appear to have two phases in their life cycle when connected to oxygen (explaining the two clusters in Figure 5B): period of growths, in which the suboxic cells divert the energy gained from sulfide oxidation toward biomass synthesis and poly-P production, and periods of growth arrest, where the suboxic cells divert the energy gained from sulfide oxidation exclusively toward poly-P production. In both cases, only the cells that perform sulfide oxidation are capable of ATP formation (Figure 7). The synchronized growth and division observed in previous research indicates that cells regularly lose their connection to oxygen as part of their life cycle (Geerlings et al., 2021). When disconnected from oxygen, both poly-P synthesis and growth cannot happen via long-distance electron transport. Thus, the variability in the ^18^O labeling of the poly-P granules between filaments can be explained by access to oxygen and differences in the cell-cycle stage (growth vs. non-growth). The cells that perform oxygen reduction do not show energy conservation, and as a result show vanishing rates of biomass synthesis (^13^C labeling) and poly -P formation (^18^O labeling) (Figure 7).

### Possible Roles of Polyphosphates in Cable Bacteria

For most poly-P accumulating organisms, poly-P is typically thought of as a “back-up” energy reservoir, but in many species it can also be essential for survival of the non-growing stage of the cell cycle or in response to oxidative stress (Rao and Kornberg, 1996; Ault-Riché et al., 1998; Kornberg et al., 1999; Rao et al., 2009; Gray et al., 2014; Gray and Jakob, 2015). We hypothesize that poly-P also plays these roles in cable bacteria, where the specific role depends on both the redox environment and cell cycle stage.

Cells in the oxic zone need to perform oxygen reduction to facilitate energy supply for the rest of the filament in the suboxic zone with no known method of energy conservation (Geerlings et al., 2020). Being in an oxic environment, these cells are likely in a constant state of oxidative stress, which is supported by the high concentrations of antioxidant proteins (e.g., catalases, superoxide reductase, rubrerythrin, and GroEL/ES chaperonins) detected by proteomic analysis of cable bacteria (Kjeldsen et al., 2019). Thus, a viable hypothesis is that cable bacteria cells use poly-P as a “back-up” energy reservoir and/or as a method of protection against oxidative stress when they (temporarily) reside in the oxic zone. Poly-P can prevent oxidative stress by acting as an ATP-independent primordial chaperone with minimal substrate specificity to stabilize a wide variety of proteins (Gray et al., 2014; Gray and Jakob, 2015). Thus, the use of poly-P as a chaperone would be ideal for cells in the oxic zone as they cannot build extra antioxidant proteins due to their inability to generate energy. Furthermore, no phosphate groups are removed from poly-P when it functions as a chaperone, so once the poly-P chain is separated from the accompanying protein, it can still be used as an energy reserve to support other tasks (e.g., to aid motility).

Microbial cells spend most of the time in a prolonged non-growing stage, with very little to no metabolic activity and growth occurring due to a limited availability of energy or nutrients, but are prepared to undergo rapid division cycles once these resources become available (Bergkessel et al., 2016). Conversely, depletion of a resource leads to a transition from a growing to a non-growing stage. The cell cycle is not arrested randomly during this transition, since this could halt key processes, especially DNA replication, at stages where severe and irreparable damage could occur (Kolter, 1993; Bergkessel et al., 2016). Thus, although the non-growing stage of the bacterial life cycle is poorly understood, even for model organisms, it is required and the transition between the growing and non-growing stage is regulated to ensure cell viability (Bergkessel et al., 2016). Previous research showed an involvement of poly-P during the non-growing stage of bacteria. For the model bacterium *Escherichia coli*, for example, mutants lacking *ppk* and thus the ability to synthesize poly-P failed to survive the state of growth arrest and lacked resistance to several stressors (e.g., heat, H_2_O_2_, or osmotic stress) (Rao and Kornberg, 1996; Shiba et al., 1997; Ault-Riché et al., 1998; Grillo-Puertas et al., 2016). For *Pseudomonas aeruginosa*, poly-P accumulation was also maximized during the non-growing stage (Kim et al., 1998).

Based on this evidence, we hypothesize that poly-P plays an important, perhaps regulatory, role during the non-growing stage of the cable bacteria cell cycle. This hypothesis is supported by the significant ^18^O labeling of poly-P granules in filaments with no, or very little, labeling in ^13^C, which was observed for filaments retrieved from all redox zones (Figure 5 and Table 4). This observation indicates that, in the suboxic zone, poly-P synthesis is one of the metabolic activities performed by cable bacteria during the non-growing stage of their cell cycle. For the filament fragments retrieved from the oxic zone, this observation suggests that cell growth is arrested earlier than poly-P synthesis. Recent research on *E. coli* showed that the ability of a cell to survive prolonged periods of starvation was dependent on the conditions prior to starvation where cells experiencing slower growth prior to starvation were shown to have consistently lower death rates (Biselli et al., 2020). In general, when a cell adjusts it physiology toward growth, it becomes less adapted for survival and vice versa. Synthesizing proteins that protect cells and increase their survival chances comes at the expense of synthesizing proteins needed for growth. The result is a trade-off between a fitness benefit in starvation and a fitness cost during growth (Scott et al., 2010). Thus, it appears that before cells of a filament residing in the suboxic zone enter the oxic zone and therefore a non-growing stage, they are already “prepared” to deal with oxidative stress and shortage of energy by entering into a stage of growth arrest which might enhance the chances of survival and increase the time a cell can survive in the oxic zone and thus provide energy for the cells in the suboxic zone below.

## Conclusion and Outlook

The ubiquitous presence of ^18^O-labeled poly-P, observed after 6 h and 24 h of incubation with ^18^O-labeled water in almost all cable bacterium filaments collected from all sediment redox zones, highlights the importance of this inorganic molecule for the metabolism of cable bacteria. ^18^O labeling was highly variable among poly-P granules, with most of the variability explained by differences among filaments and a lesser part by differences among granules of the same cell. Additionally, the average ^18^O labeling of poly-P was significantly lower in filaments from the oxic zone compared to those from the transition and suboxic zones, confirming that energy conservation is principally coupled to sulfide oxidation and not oxygen reduction. The variability patterns observed in our combined ^18^O and ^13^C data can be explained by assuming that, within an individual cable bacterium filament, poly-P is synthesized by cells residing in the suboxic zone (using energy gained from the sulfide oxidation half-reaction) and broken down by cells in the oxic zone. These roles dynamically change as part of the filament migrates in and out of the oxic zone, and our data indicate that a significant fraction of poly-P stored by cable bacteria is turned over in this way on the timescale of 24 h. Our data also indicate that cable bacteria migrate in and out of the oxic zone in a regulated fashion where cell growth is halted before entering the oxic zone. We hypothesize that, in the oxic zone, poly-P is used as a chaperone to aid protection against oxidative stress or as an energy reserve that can be utilized for other tasks (e.g., to aid motility), while in the suboxic zone, poly-P plays an important, perhaps regulatory role, during the non-growing stage of the cable bacteria cell cycle.

Our results here provide a set of hypotheses about poly-P cycling in cable bacteria. Additional research is required to test these hypotheses and quantify the rates, regulation and environmental impact of poly-P synthesis and breakdown in cable bacteria. Ideally, such future studies will combine molecular methods (e.g., genomic and proteomic analyses) with biogeochemical analyses including chemical imaging. Our results here show that dual-label stable isotope probing using H_2_^18^O and ^13^C-DIC forms a viable approach to shed light on C and P metabolism in cable bacteria, but also microorganisms in general. Yet, future experiments need to be conducted on shorter time scales (∼1 h) to better resolve the dynamics of poly-P synthesis and turnover within cable bacteria.

## Data Availability Statement

The original contributions presented in the study are included in the article/Supplementary Material, further inquiries can be directed to the corresponding author/s.

## Author Contributions

NG, DV-C, FM, JM, and LP conceived the study. NG, DV-C, and SH-M set up the enrichment culture and prepared all the samples for nanoSIMS analysis. MK and LP performed the nanoSIMS analysis. NG, MK, RH, and LP analyzed the nanoSIMS data. NG wrote the manuscript with contributions from all co-authors. All authors contributed to the article and approved the submitted version.

## Conflict of Interest

The authors declare that the research was conducted in the absence of any commercial or financial relationships that could be construed as a potential conflict of interest.

## Publisher’s Note

All claims expressed in this article are solely those of the authors and do not necessarily represent those of their affiliated organizations, or those of the publisher, the editors and the reviewers. Any product that may be evaluated in this article, or claim that may be made by its manufacturer, is not guaranteed or endorsed by the publisher.
