# Supplementary material for: Polyphosphate Dynamics in Cable Bacteria
Source: Front Microbiol. 2022 May 19;13:883807. doi: 10.3389/fmicb.2022.883807 (PMC9159916; doi:10.3389/fmicb.2022.883807)
Supplement: Supplementary file 1 [file Image_2.PDF]

Supplementary Material – Figures and tables

The role of polyphosphates in the metabolism of cable bacteria

Nicole M. J. Geerlings[1*], Michiel V.M. Kienhuis[1], Silvia Hidalgo-Martinez[2,3], Renee Hageman[1], Diana Vasquez-Cardenas[2], Jack J. Middelburg[1], Filip J. R. Meysman[2,3], Lubos Polerecky[1*]

[1]Department of Earth Sciences, Utrecht University, Princetonplein 8a, 3584 CB Utrecht, The Netherlands
[2]Excellence centre for Microbial Systems Technology, University of Antwerp, Universiteitsplein 1, B-2610 Wilrijk, Belgium
[3] Department of Biotechnology, Delft University of Technology, Van der Maasweg 9, 2629 HZ Delft, The Netherlands

Supplementary Figures

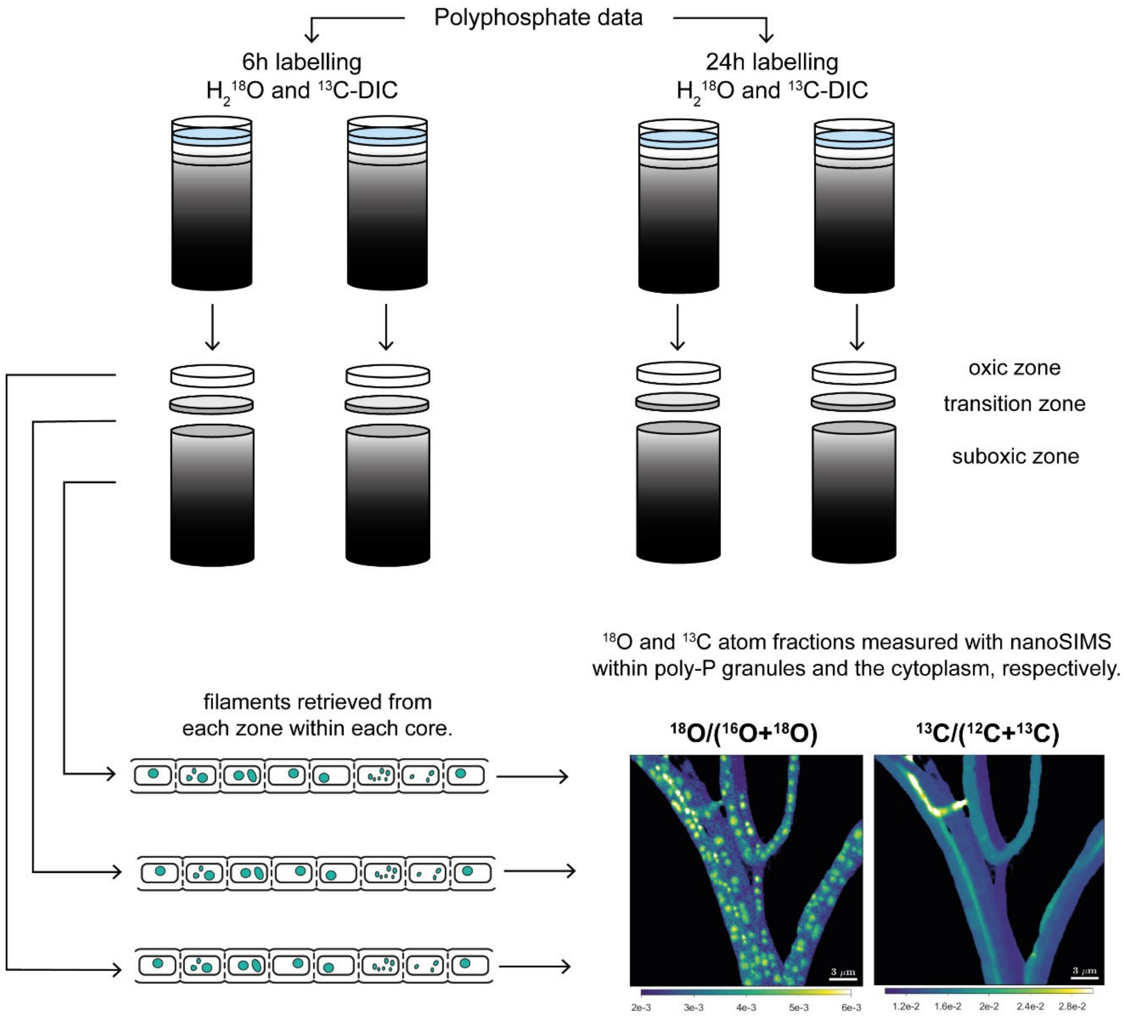

Figure S1: Flow chart depicting the experimental and analytical set-up. Four sediment cores with enrichment cultures of cable bacteria were incubated with ^{13}C-labeled bicarbonate and ^{18}O-labeled water. Two cores were incubated for 6 h and two cores for 24 h. At the end of incubation, each core was sliced into three zones based on the redox environment (oxic, transition and suboxic) and from each zone cable bacteria filaments were hand-picked and prepared for nanoSIMS analysis. After the nanoSIMS analysis, regions of interest (ROIs) were drawn around individual polyphosphate granules and the corresponding ROI-specific ^{13}O and ^{18}O atom fractions were quantified using Look@NanoSIMS.

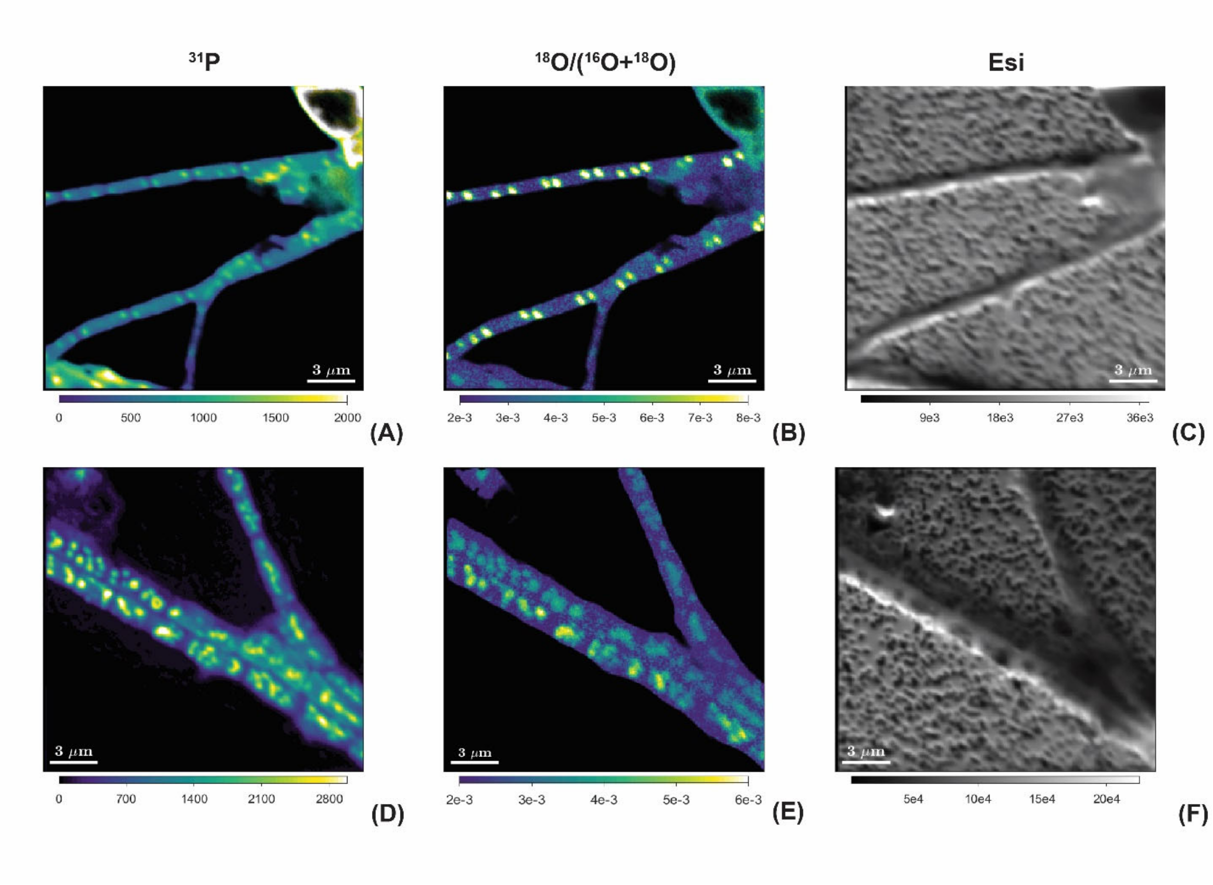

Figure S3: Representative NanoSIMS images of **(A-C)** the "thin" and **(D-F)** "thicker" morphotype of cable bacteria measured in this study. Shown are **(A, D)** ^{31}P ion counts (accumulated over all measured planes), **(B, E)** ^{18}O atom fractions, and **(C, F)** secondary electrons. All scale bars are 3 µm.

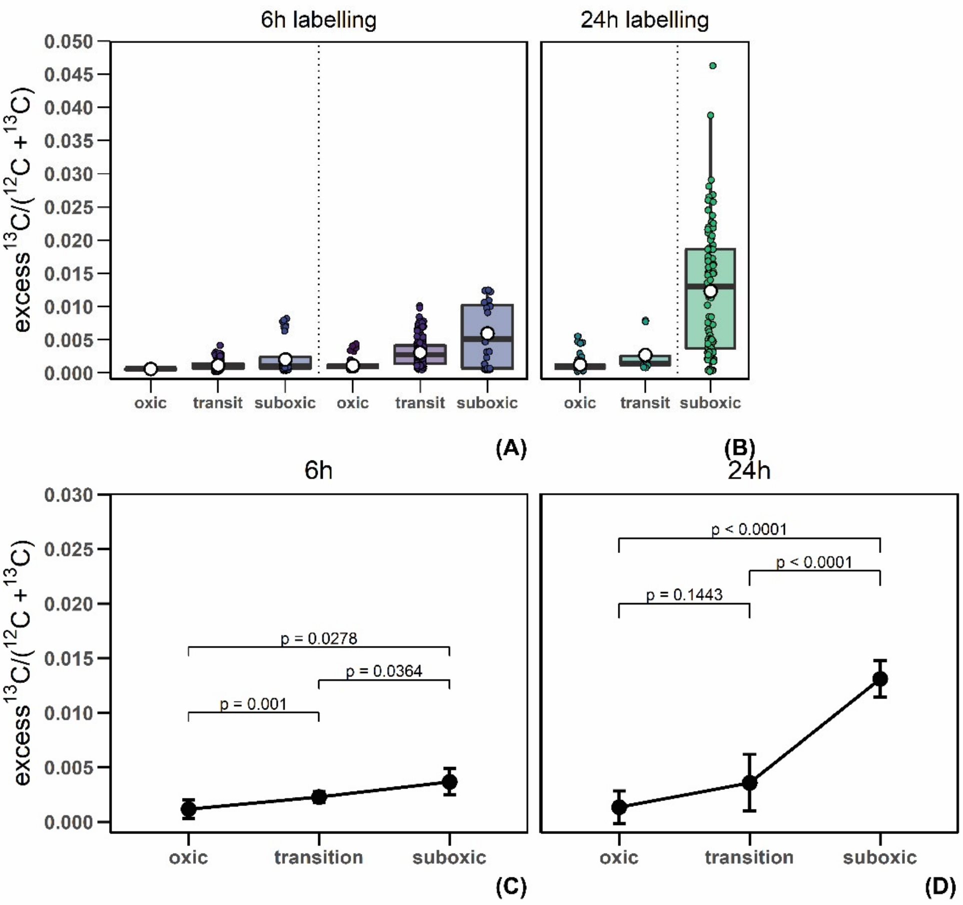

Figure S4: ^{13}C labelling of the cytoplasm of cable bacteria. **(A-B)** Boxplots show excess ^{13}C atom fractions in individual cells, separately for the 6 h and 24 h labelling periods. Dotted lines separate data obtained from replicate sediment cores. Shown are the corresponding mean (white open dot), median (black line) and upper and lower quantiles of the excess ^{13}C atom fraction. Note that grouping of cells within filaments is not indicated in the graphs. **(C-D)** Results of the linear mixed model used for fitting the variance in the excess ^{13}C atom fractions in cable bacteria cells. Results are shown separately for the 6 h and 24 h labelling period. Black circles and error-bars depict the best estimates and the lower and upper confidence limits (95%) of the mean value for each zone and labelling period (exact values provided in **Supplementary Table S3**). The p-values indicate the significance of differences between zones (summarized in **Supplementary Table S4**). Raw data is provided as **Dataset S2**, the step-by-step build-up and outcomes of the linear mixed model are available in **Supplementary Methods**.

Supplementary Tables

Table S1: Outcome of the linear mixed model used for fitting the variance in the ^{18}O labelling of poly-P granules in cable bacteria. Shown are the fitted values, standard errors, lower and upper limits for each combination of the redox zone and labelling period. These values were used to produce the graphs in **Figure 4D-E**.

zone	labelling	fit	se	lower	upper
oxic	6h	0.000607	0.00018	0.000253	0.000961
transition	6h	0.00175	0.00010	0.00155	0.00195
suboxic	6h	0.00130	0.00036	0.00078	0.00182
oxic	24h	0.00158	0.00024	0.00110	0.00205
transition	24h	0.00320	0.00036	0.00245	0.00390
suboxic	24h	0.00363	0.00020	0.00324	0.00401

Table S2: p-values determined by the linear mixed model used for testing whether differences in the ^{18}O labelling of poly-P granules are statistically significant when compared between specific combinations of redox zones and labelling periods.

		6h			24h	
		**oxic**	**transition**	**suboxic**	**oxic**	**transition**
**6h**	**transition**	< 0.0001	-			
	**suboxic**	0.0326	0.1140	-		
**24 h**	**oxic**	0.0016	0.3151	0.0027	-	
	**transition**	0.3151	0.0001	0.0772	0.0002	-
	**suboxic**	0.0027	0.0772	< 0.0001	< 0.0001	0.2909

Table S3: Outcome of the linear mixed model used for fitting the variance in the ^{13}C labelling of the cytoplasm in cable bacteria. Shown are the fitted values, standard errors, lower and upper limits for each combination of the redox zone and labelling period. These values were used to produce the graphs in **Figure S4 C-D.**

zone	labelling	fit	se	lower	upper
oxic	6h	0.001157	0.000444	0.000285	0.002029
transition	6h	0.002281	0.000243	0.001805	0.002757
suboxic	6h	0.003667	0.000610	0.002469	0.004865
oxic	24h	0.001346	0.000757	-0.000139	0.002832
transition	24h	0.003582	0.001322	0.000985	0.006178
suboxic	24h	0.013107	0.0008586	0.011421	0.014793

Table S4: p-values determined by the linear mixed model used for testing whether differences in the ^{13}C labelling of cable bacteria cells are statistically significant when compared between specific combinations of redox zones and labelling periods.

		6h			24h	
		**oxic**	**transition**	**suboxic**	**oxic**	**transition**
**6h**	**transition**	0.001	-			
	**suboxic**	0.0278	0.0364	-		
**24 h**	**oxic**	0.8296	0.4898	< 0.0001	-	
	**transition**	0.4898	0.3348	< 0.0001	0.1443	-
	**suboxic**	< 0.0001	< 0.0001	< 0.0001	< 0.0001	< 0.0001
