# Supplementary material for: Polyphosphate Dynamics in Cable Bacteria
Source: Front Microbiol. 2022 May 19;13:883807. doi: 10.3389/fmicb.2022.883807 (PMC9159916; doi:10.3389/fmicb.2022.883807)
Supplement: Supplementary file 2 [file Image_1.PDF]

Figure S2: The ^{18}O atom fraction (defined as $^{18}O/(^{16}O+^{18}O)$), the ^{13}C atom fraction (defined as $^{13}C^{14}N/(^{12}C^{14}N+^{13}C^{14}N)$) and the relative phosphorus content (defined as $^{31}P/(^{12}C^{14}N+^{13}C^{14}N)$) of all measured Fields of view (FOV). For FOV where the $^{12}C^{14}N^{-}$ ion count was not measured (see Methods), the ^{13}C atom fraction could not be calculated and the relative phosphorus content was defined as ^{31}P/plane/pixel. Scale bars are all 3 µm. Colour bars are between 0.002-0.006 for the ^{18}O atom fraction, between 0.010-0.030 for the ^{13}C atom fraction and between 0.02-0.04 for the relative phosphorus content when defined as $^{31}P/(^{12}C^{14}N+^{13}C^{14}N)$. The colour bars for the relative phosphorus content when defined as ^{31}P/plane/pixel are variable.

6 h incubation

oxic zone

core 1

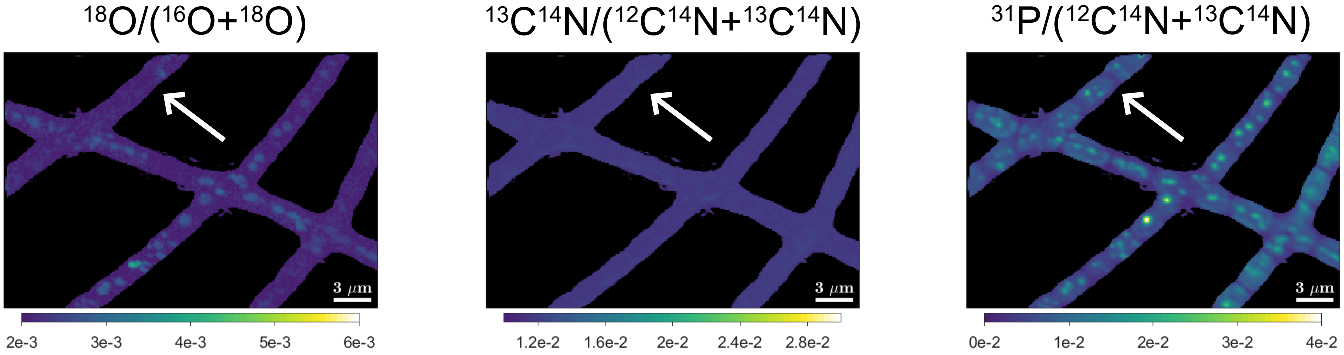

The white arrow points toward a filament fragment that posesses poly-P granules but shows minimal activity in the ^{18}O atom fraction of the polyphosphate granules and no growth (i.e. no ^{13}C assimilation) during the incubation period. This filaments fragment was thus considered inactive (the polyphosphate excess ^{18}O atom fraction was below 0.0004 and the excess ^{13}C atom fraction was equal or below 0.001.

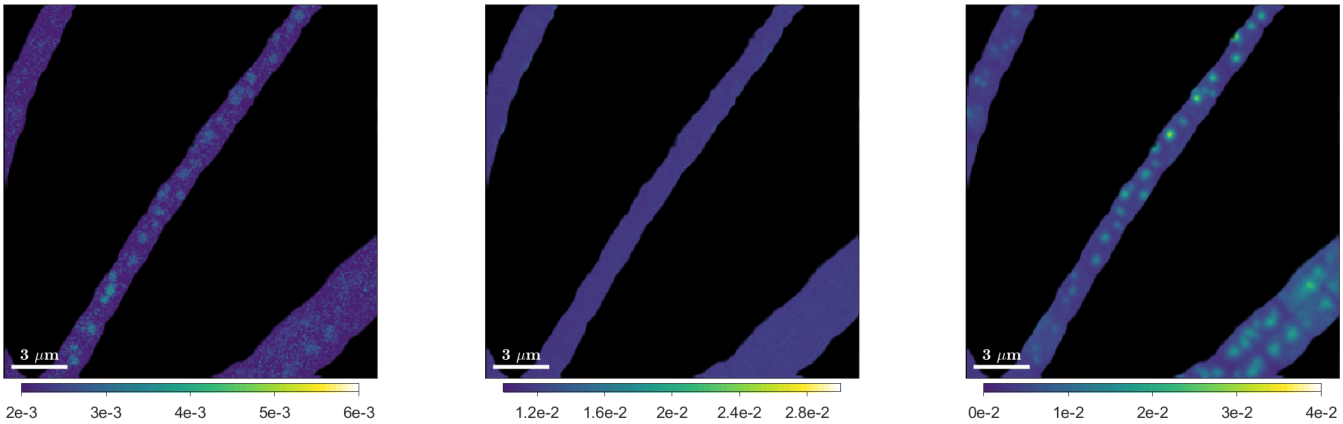

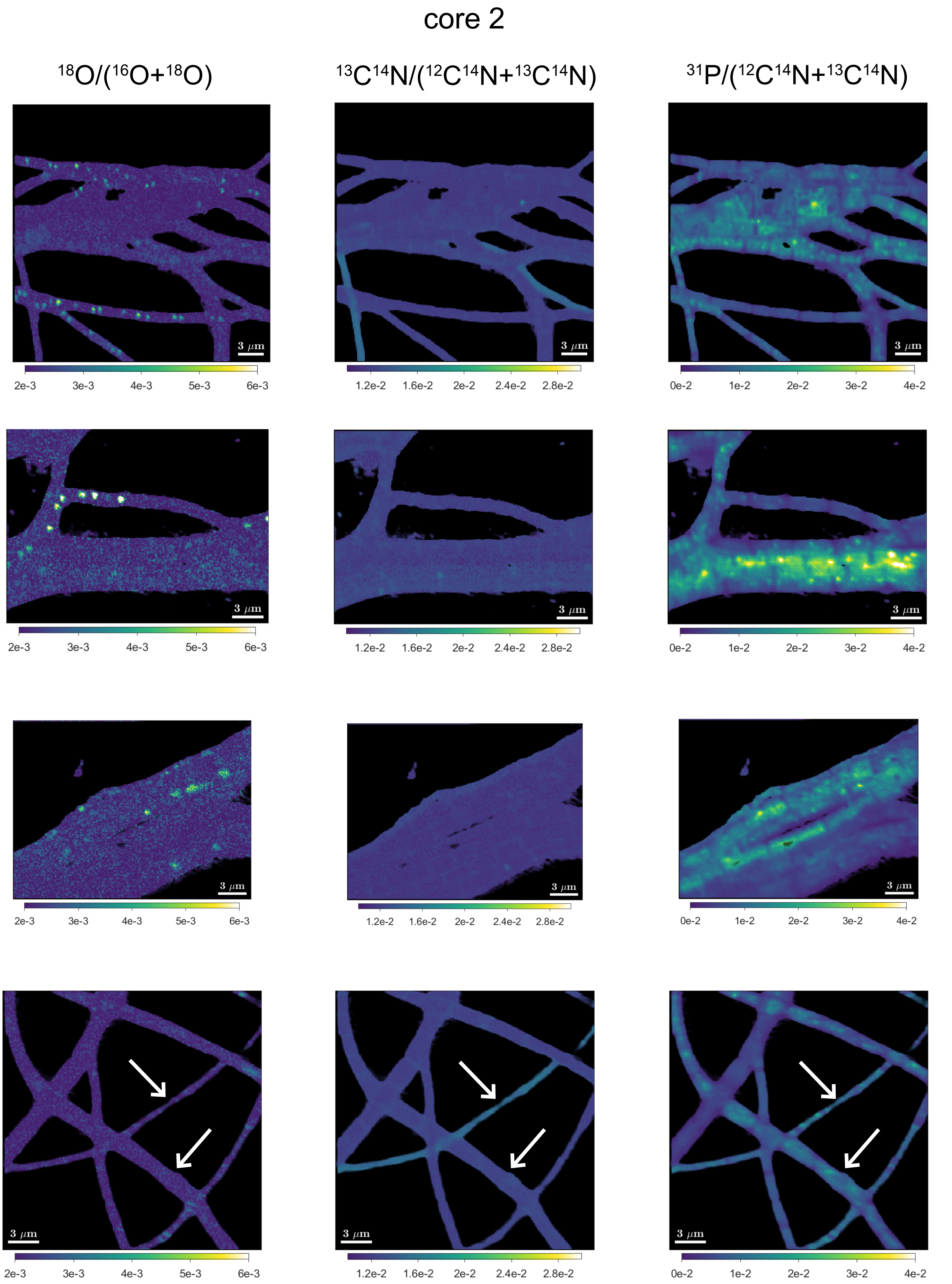

The white arrows points toward filament fragments that posess poly-P granules but show no or minimal activity with respect to the ^{18}O atom fraction of the polyphosphate granules. Both filament fragments do show ^{13}C assimilation during the incubation period (i.e. excess ^{13}C atom fraction > 0.001).

transition zone
core 1

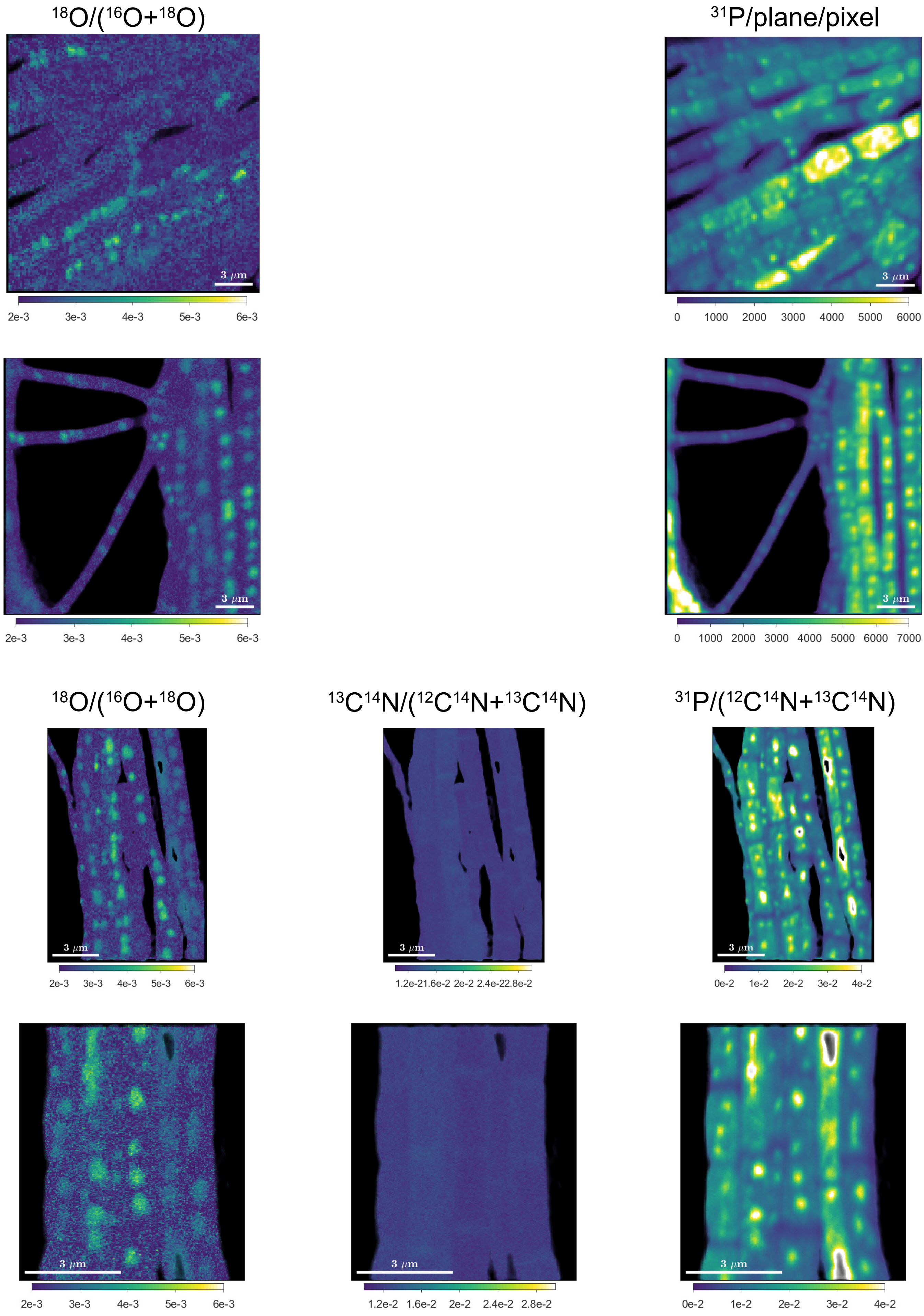

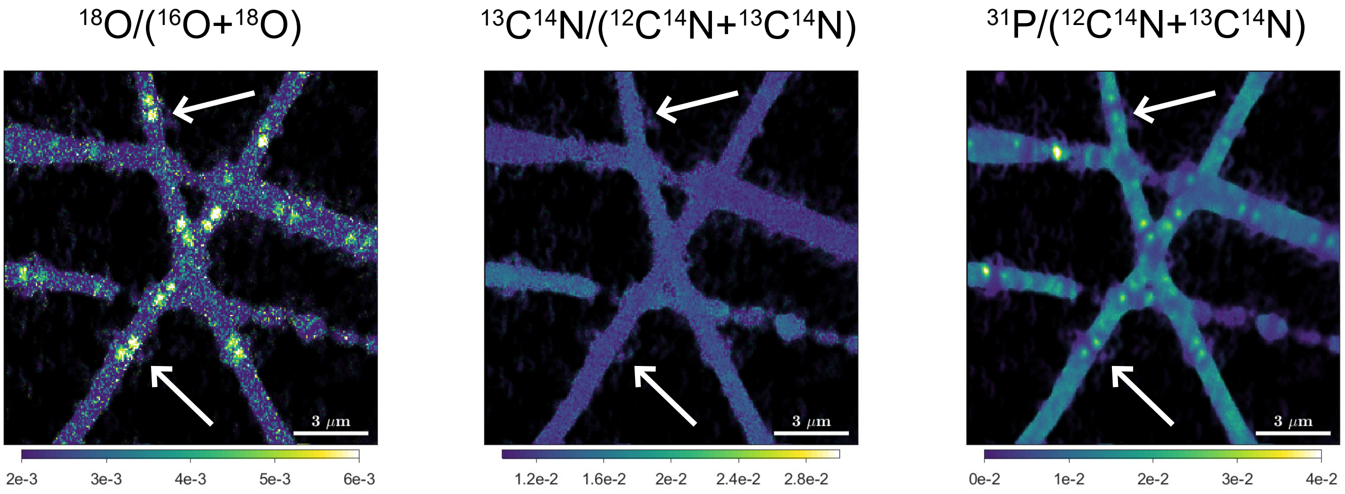

The white arrows points toward two filament fragment that posess poly-P granules that have been active during the labelling period which is reflected in the increased ^{18}O atom fraction. However, both filaments were inactive with respect to their carbon metabolism (no or minimal ^{13}C atom fraction).

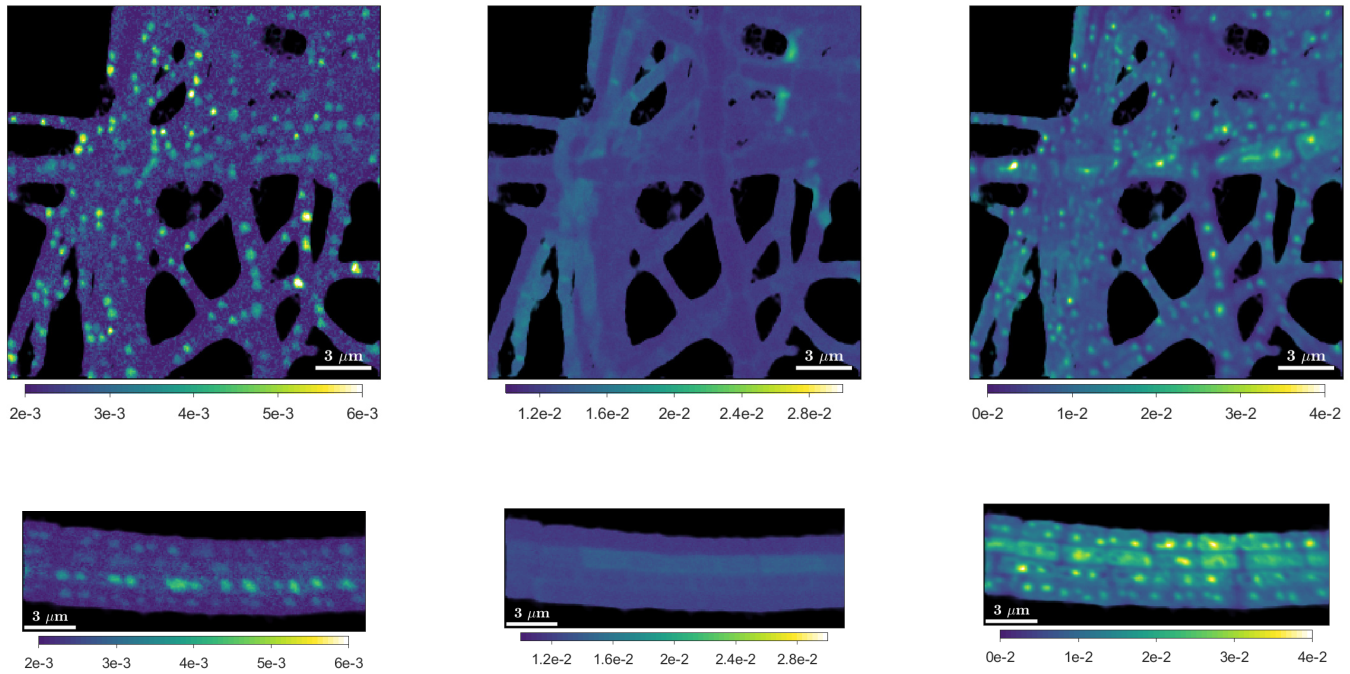

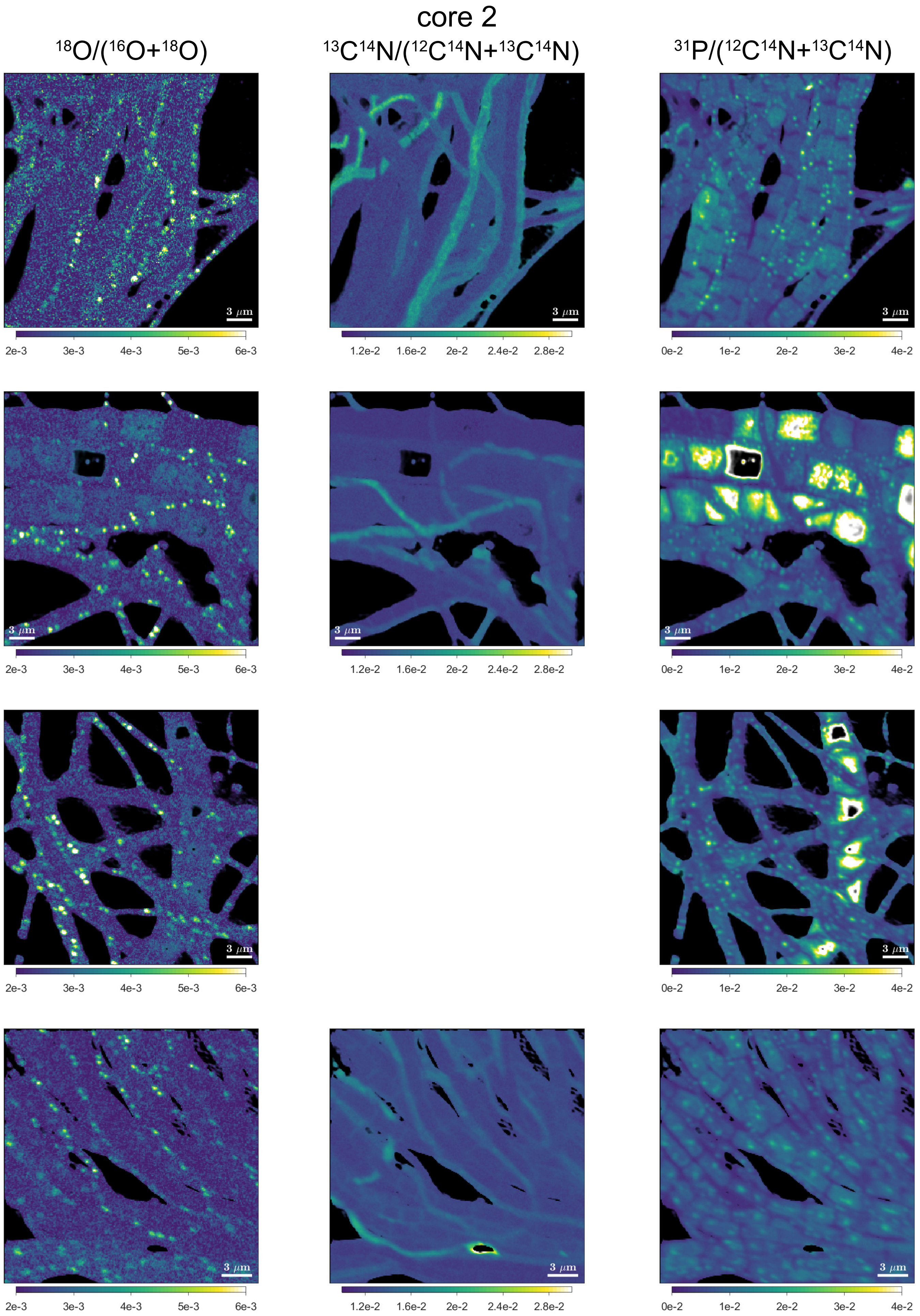

core 2
18O/(16O+18O)
13C14N/(12C14N+13C14N)
31P/(12C14N+13C14N)
3 μm
2e-3
3e-3
4e-3
5e-3
6e-3
1.2e-2
1.6e-2
2e-2
2.4e-2
2.8e-2
0e-2
1e-2
2e-2
3e-2
4e-2

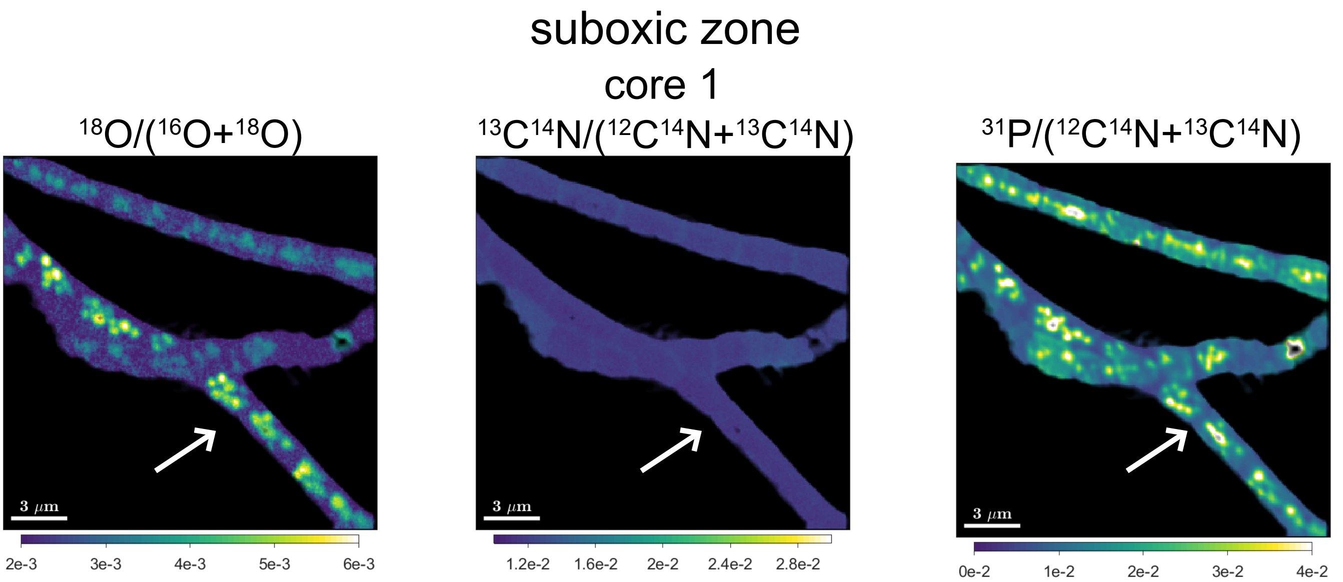

The white arrows points toward a filament fragment that posesses poly-P granules that have been active during the labelling period which is reflected in the increased ^{18}O atom fraction. However, both filaments were inactive with respect to their carbon metabolism.

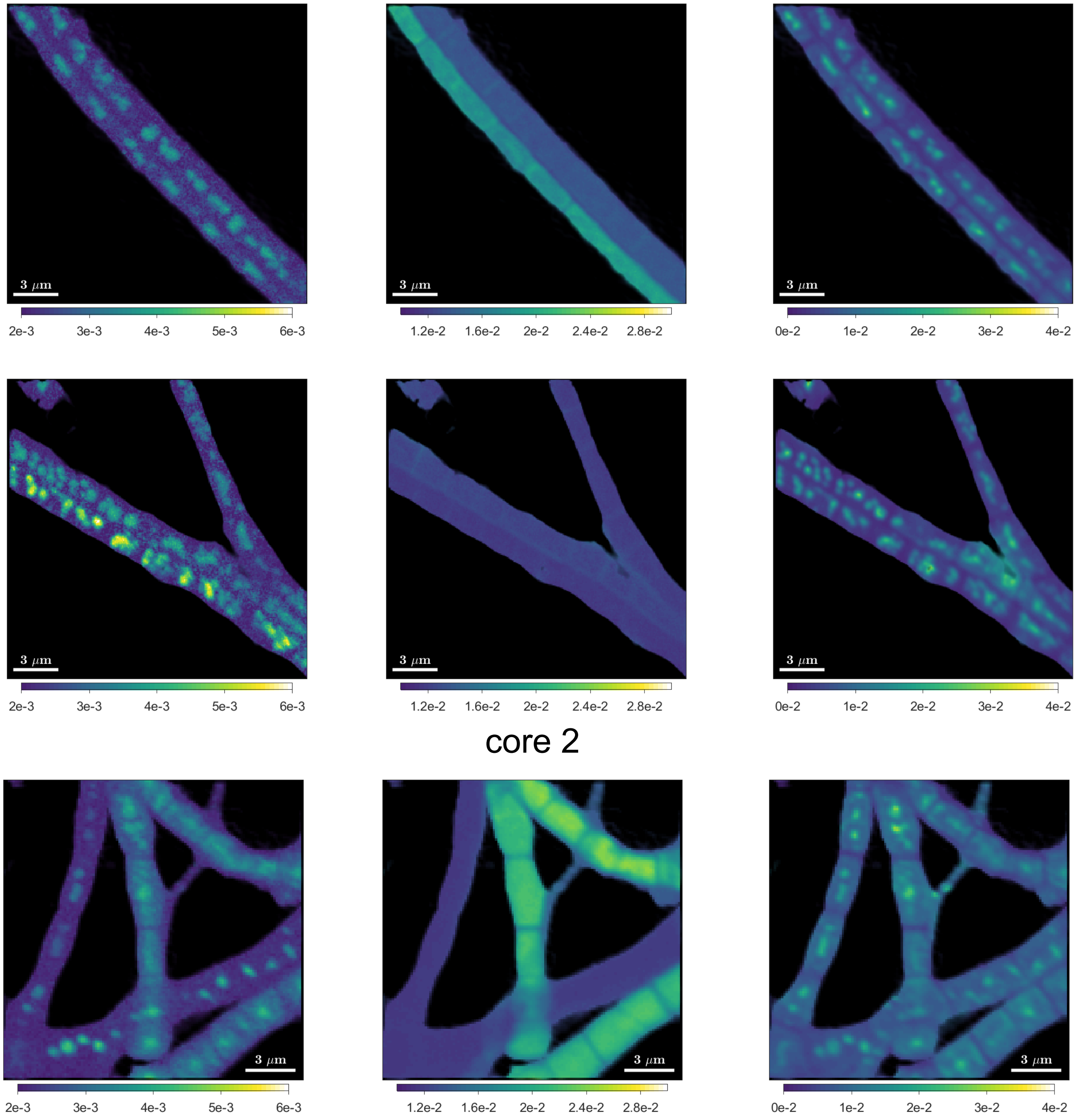

24 h incubation

oxic zone

core 1

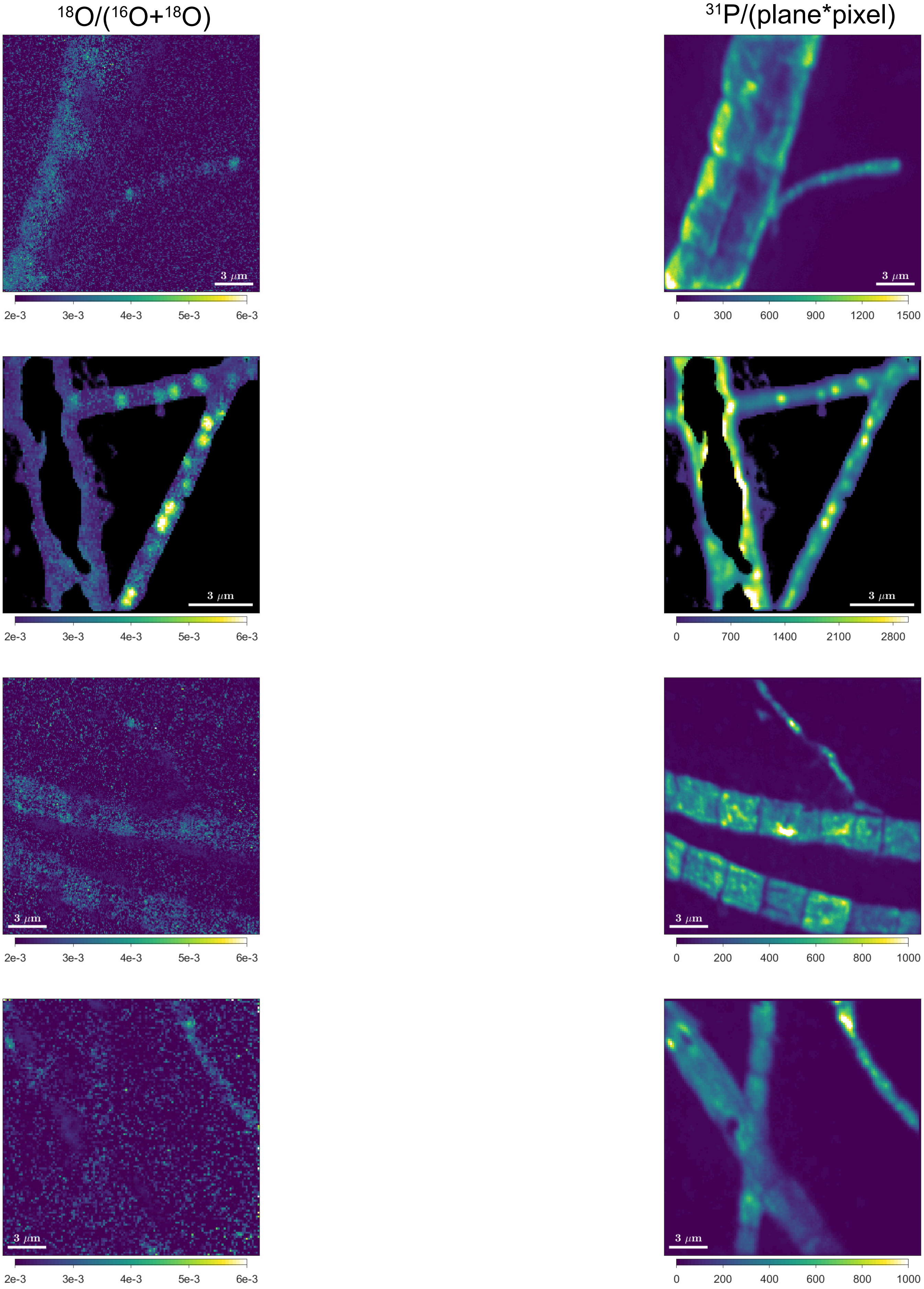

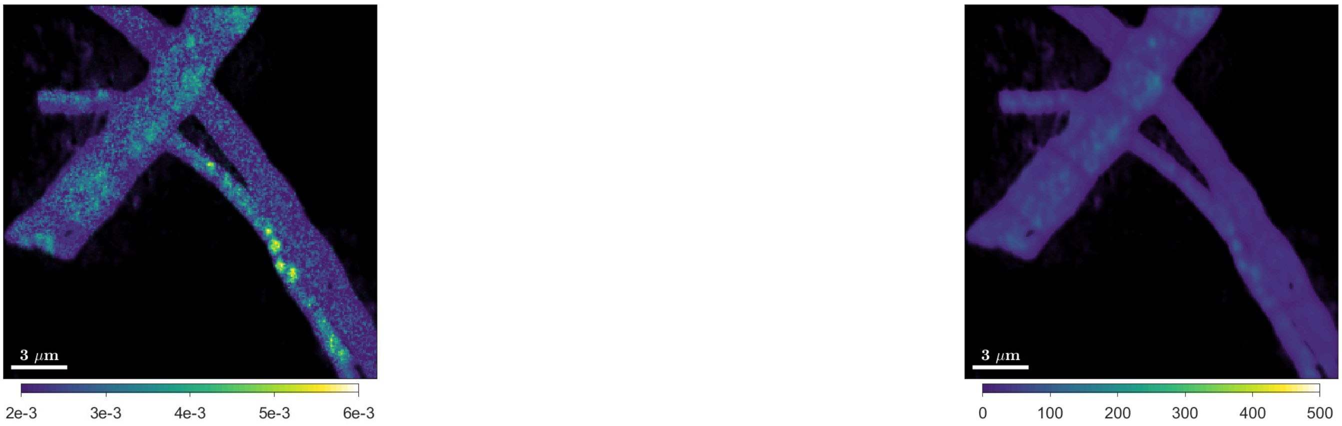
3 μm
2e-3 3e-3 4e-3 5e-3 6e-3
3 μm
0 100 200 300 400 500

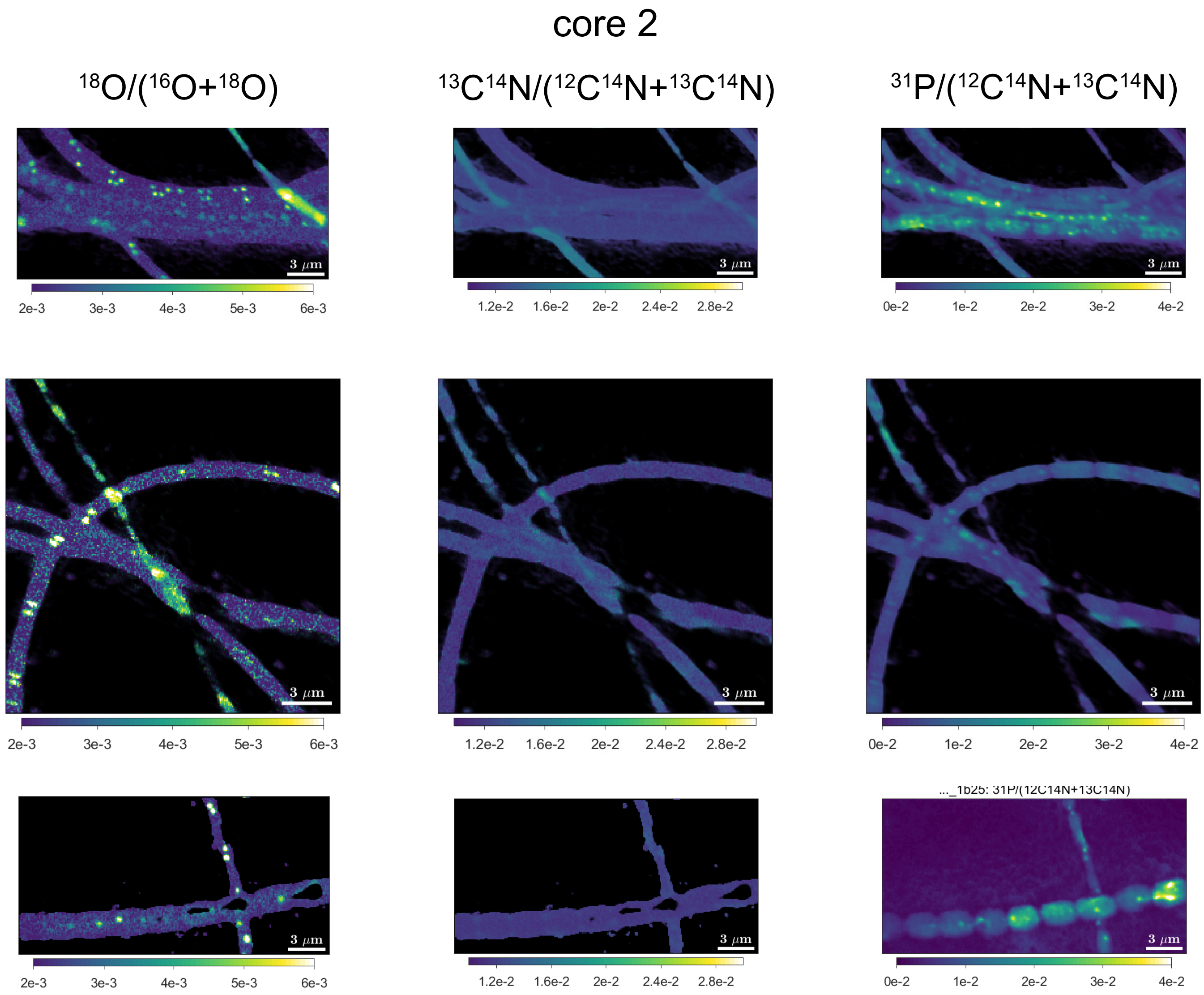
core 2
18O/(16O+18O)
13C14N/(12C14N+13C14N)
31P/(12C14N+13C14N)
3 μm
2e-3 3e-3 4e-3 5e-3 6e-3
1.2e-2 1.6e-2 2e-2 2.4e-2 2.8e-2
0e-2 1e-2 2e-2 3e-2 4e-2
..._1b25: 31P/(12C14N+13C14N)

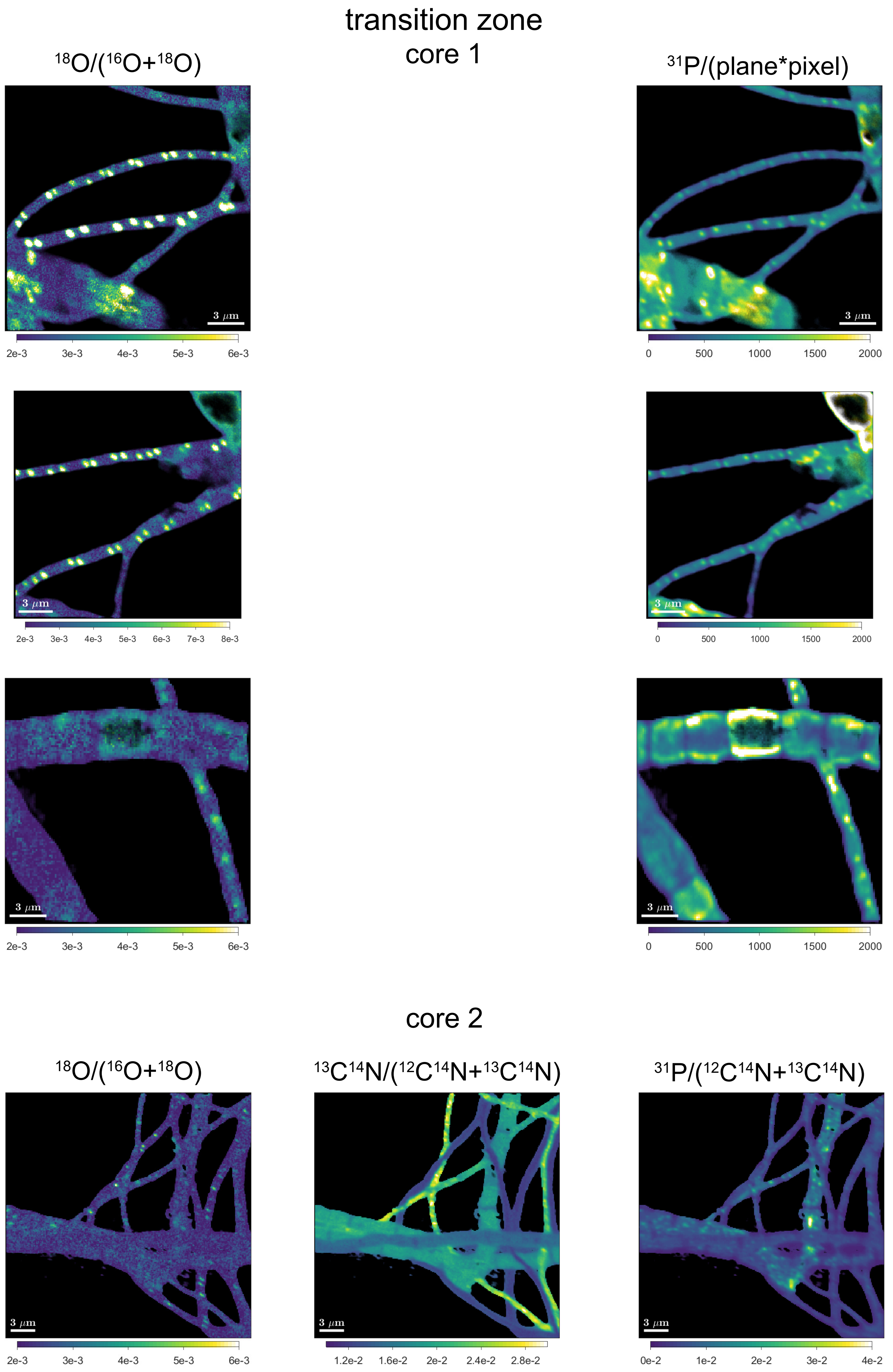

transition zone
core 1
18O/(16O+18O)
31P/(plane*pixel)
3 μm
2e-3 3e-3 4e-3 5e-3 6e-3
0 500 1000 1500 2000
2e-3 3e-3 4e-3 5e-3 6e-3 7e-3 8e-3
0 500 1000 1500 2000
2e-3 3e-3 4e-3 5e-3 6e-3
0 500 1000 1500 2000
core 2
18O/(16O+18O)
13C14N/(12C14N+13C14N)
31P/(12C14N+13C14N)
2e-3 3e-3 4e-3 5e-3 6e-3
1.2e-2 1.6e-2 2e-2 2.4e-2 2.8e-2
0e-2 1e-2 2e-2 3e-2 4e-2

suboxic zone
core 1

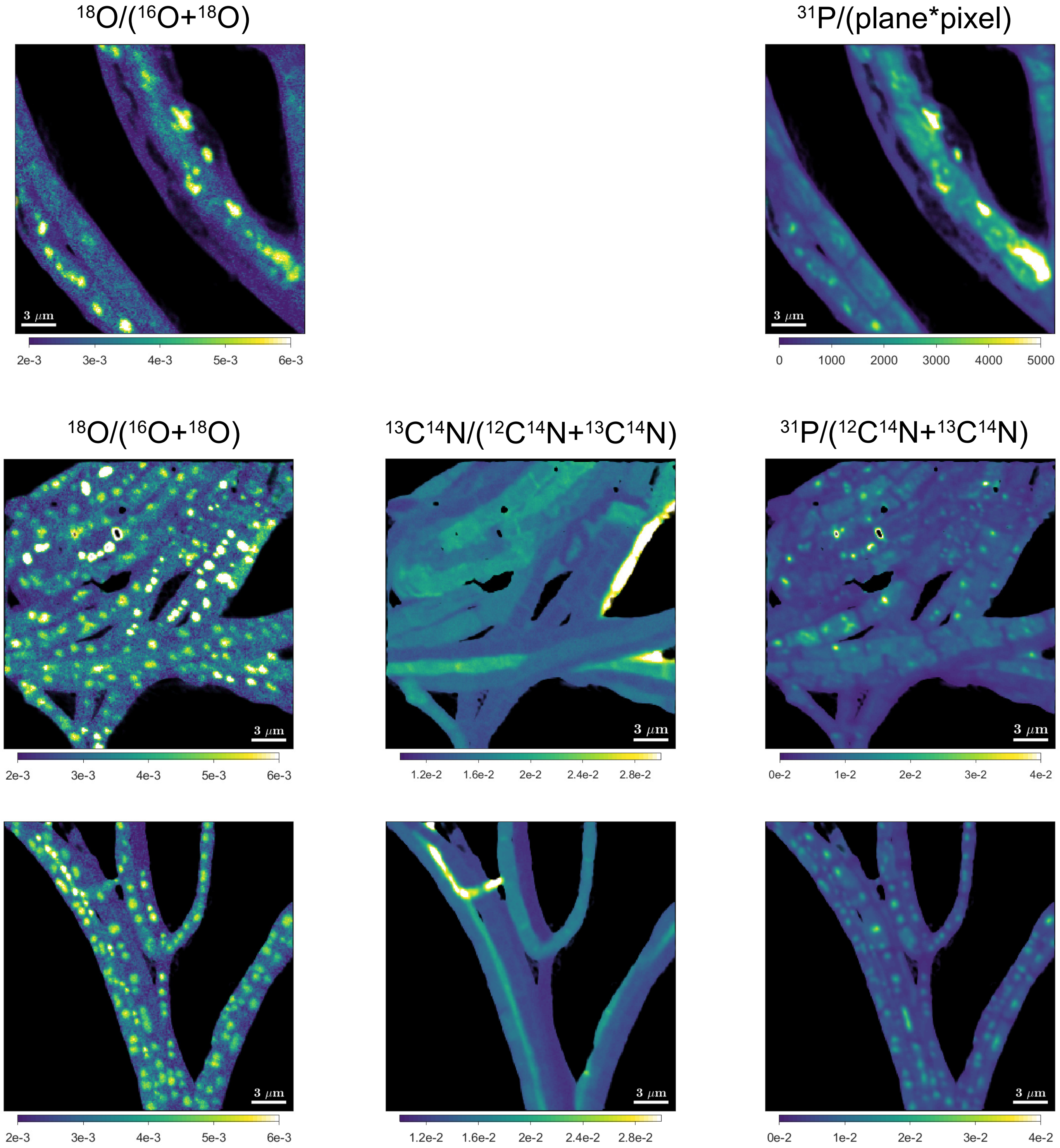

core 2

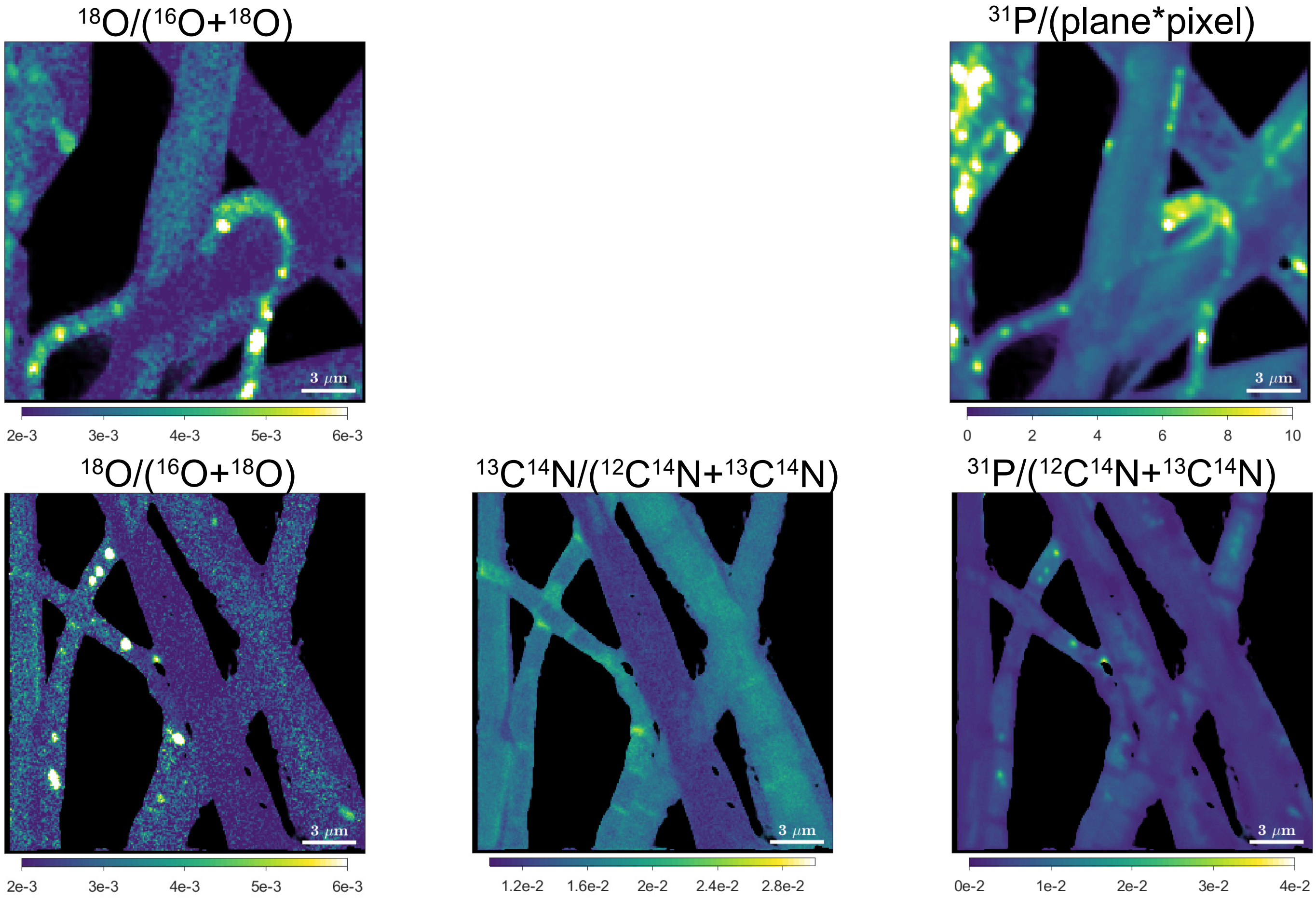

$^{18}O/(^{16}O+^{18}O)$
^{31}P/(plane*pixel)
3 μm
2e-3 3e-3 4e-3 5e-3 6e-3
0 2 4 6 8 10
$^{18}O/(^{16}O+^{18}O)$
$^{13}C^{14}N/(^{12}C^{14}N+^{13}C^{14}N)$
$^{31}P/(^{12}C^{14}N+^{13}C^{14}N)$
1.2e-2 1.6e-2 2e-2 2.4e-2 2.8e-2
0e-2 1e-2 2e-2 3e-2 4e-2
