# Supplementary material for: Polyphosphate Dynamics in Cable Bacteria
Source: Front Microbiol. 2022 May 19;13:883807. doi: 10.3389/fmicb.2022.883807 (PMC9159916; doi:10.3389/fmicb.2022.883807)
Supplement: Supplementary file 5 [file Presentation_1.PDF]

Modeling of 18O labeling of poly-P inclusions in cable bacteria

Here, we develop a simple model for predicting ^{18}O labeling of poly-P inclusions in cable bacteria. The model simulates conditions during the stable isotope probing experiment conducted in this study. The aim of the model is to identify realistic scenarios of poly-P synthesis and breakdown that could explain the observed data.

The model is based on mass balances and uses oxygen as the model currency. Thus, the amount of poly-P is approximated by the amount of oxygen atoms it contains. We neglect the effects of transport, i.e., we assume that the modeled oxygen reservoirs are well-mixed on the time-scale of the model.

In accordance with the observations of Blake et al. (2005), we assume that the increase over time in the ^{18}O atom fraction of the porewater phosphate (P_i) pool is described by the equation

$$x_{Pi} = x_{ini} + (x_S - x_{ini}) \cdot (1 - e^{-k \cdot t}), \qquad (1)$$

where $x_{ini} = 0.002$ is the initial value (corresponding to the natural abundance of ^{18}O), $x_S = 0.025$ is the ^{18}O atom fraction of the porewater (estimated from the amount and labeling of the ^{18}O-labeled stock solution injected into each sediment core; see Methods), and $k = 0.125\ h^{-1}$ is the rate constant describing the ^{18}O exchange between the P_i pool and ^{18}O-water (estimated from data in Blake et al., 2005).

The model is set up using the general framework developed by Polerecky et al. (2022). Specifically, we assume that oxygen incorporation into an individual poly-P granule occurring during poly-P synthesis, and oxygen removal from a poly-P granule occurring during poly-P breakdown, are described by zero-order kinetics. Additionally, we neglect the kinetic isotope fractionation effects for both processes. With respect to poly-P synthesis, we assume that the incorporation of ^{18}O into the poly-P granule is proportional to the ^{18}O atom fraction of the P_i pool (x_{Pi}), where the latter value describes the probability that the O atom in the P_i pool is ^{18}O. Similarly, with respect to poly-P breakdown, we assume that the removal of ^{18}O from the poly-P granule is proportional to the instantaneous ^{18}O atom fraction of the poly-P granule, where the latter value describes the probability that the O atom in a well-mixed poly-P granule is ^{18}O. Thus, the *addition* of total O (i.e., $^{18}O + ^{16}O$) and ^{18}O into an individual poly-P granule during poly-P synthesis is described by differential equations 2, whereas the *removal* of total O and ^{18}O from an individual poly-P granule during poly-P breakdown is described by the differential equations 3.

$$\begin{aligned} \frac{dO}{dt} &= r_{synth}, \\ \frac{d^{18}O}{dt} &= r_{synth} \cdot x_{Pi}, \end{aligned} \qquad (2)$$

$$\begin{aligned} \frac{dO}{dt} &= -r_{break}, \\ \frac{d^{18}O}{dt} &= -r_{break} \cdot x. \end{aligned} \qquad (3)$$

In equation 3, x denotes the instantaneous ^{18}O atom fraction of the poly-P granule, which is defined as

$$x = \frac{^{18}O}{O}. \qquad (4)$$

Model implementation in R

We solve the above differential equations using R (R Core Team, 2020) and the R-package `deSolve` (Soetaert et al. 2010).

Scenario 1

First, we assume that poly-P synthesis and incorporation into a poly-P granule occurred at a constant rate during the *entire* 24 h incubation. We choose the rate of synthesis, r_{synth}, such that the excess ^{18}O atom fraction of the poly-P granule predicted after 6 h is equal to the average value measured at this time point. Then we use the model to predict the excess ^{18}O atom fraction after 24 h.

```
# Initial conditions and other model parameters:
x.ini    <- 0.002     # initial 18O atom fraction
xS       <- 0.025     # final 18O atom fraction of the Pi
tmax     <- 24        # incubation period
k        <- 3/tmax*1  # rate of equilibration of the Pi pool with 18O-water
Otot.ini <- 1         # O-content of poly-P inclusion (arbitrary)

# experimental data:
define_expdata <- function(zone=1) {
  # excess 18O atom fractions (data from Supplementary Table S2)
  incub.time <- c(6,24)                              # time points (h)
  if (zone==1) xE <- c(0.002676, 0.003646) - x.ini # oxic zone
  if (zone==2) xE <- c(0.003816, 0.005267) - x.ini # transition zone
  if (zone==3) xE <- c(0.003366, 0.005698) - x.ini # suboxic zone
  return(data.frame(time=incub.time, xE_polyP=xE))
}

# Scenario 1: only synthesis, no breakdown, synthesis rate is constant

# define model parameters
define_pars.1 <- function(zone=1){
  r   <- Otot.ini/2.4/tmax
  fac <- c(1, 3.4, 2.4) # factors for calculating the rate in a redox zone
                        # 1: oxic; 2: transition; 3: suboxic
  pars <- c(r_synth = r*fac[zone])
  return(pars)
}

# define model function
O18model.1 <-function(t, state, pars) {
  with (as.list(c(state, pars)),{

  x   <- O18/Otot                         # 18O atom fraction, poly-P granule
  xPi <- x.ini + (xS-x.ini)*(1-exp(-k*t)) # 18O atom fraction, Pi pool

  # RATES OF CHANGE IN THE TOTAL O AND 18O CONTENTS IN THE POLY-P GRANULE
  dO.dt   <- r_synth
  dO18.dt <- r_synth*xPi

  list(c(dO.dt, dO18.dt),
      xE_Pi    = xPi      - x.ini, # excess 18O fraction of the Pi pool (source)
```

```
      xE_polyP = O18/Otot - x.ini  # excess 18O fraction, poly-P
      )
  })
}

# solve the model
require(deSolve)
outtimes <- seq(from = 0, to = tmax, length.out = 500)
yini     <- c(Otot = Otot.ini, O18 = x.ini*Otot.ini)

# transition zone
pars2    <- define_pars.1(zone=2)
out2     <- ode(y = yini, parms = pars2, func = O18model.1, times = outtimes)
DATA2    <- define_expdata(zone=2)
# suboxic zone
pars3    <- define_pars.1(zone=3)
out3     <- ode(y = yini, parms = pars3, func = O18model.1, times = outtimes)
DATA3    <- define_expdata(zone=3)
```

```
# plot model results
plot(out2,
     which=c("xE_Pi", "xE_polyP"),
     xlab="time (h)", col=1:4, lty=1, lwd=2, mfrow=c(1,3),
     obs = DATA2, obspar = list(pch=16, col=1, cex=2),
     ylim = list(c(0,xS), c(0,0.012)))
legend("topleft", legend = c("model", "exp. data"),
       title="transition zone", col=1, lwd=2, lty=c(1,NA),
       pch=c(NA,16), bty="n")
plot(out3,
     which=c("xE_polyP"),
     xlab="time (h)", col=1:4, lty=1, lwd=2, mfrow=NULL,
     obs = DATA3, obspar = list(pch=16, col=1, cex=2),
     ylim = c(0,0.012))
legend("topleft", legend = c("model", "exp. data"),
       title="suboxic zone", col=1, lwd=2, lty=c(1,NA),
       pch=c(NA,16), bty="n")
```

Figure 1 shows that excess ^{18}O atom fractions in the poly-P granule after 24 h of incubation, as predicted by the model scenario 1, are much greater than the corresponding average values determined experimentally. Thus, it is unlikely that poly-P synthesis into individual granules occurred at a constant rate during the entire 24 h incubation period.

Scenario 2

As a more realistic scenario, we assume that, during the 24 h incubation, there was first a phase of poly-P synthesis followed by a phase of no poly-P activity (no synthesis nor breakdown, i.e., $r_{synth} = r_{break} = 0$). Similar to scenario 1, we choose the rate of synthesis, r_{synth}, such that the excess ^{18}O atom fraction of the poly-P granule predicted after 6 h is equal to the average value measured at this time point. However, in contrast to scenario 1, we now adjust the duration of the synthesis and inactivity phases such that the excess ^{18}O atom fraction predicted by the model after 24 h matches the experimentally determined value. Note that we only show *one* possible choice of model parameters for which this match between the experimental and model values can be achieved.

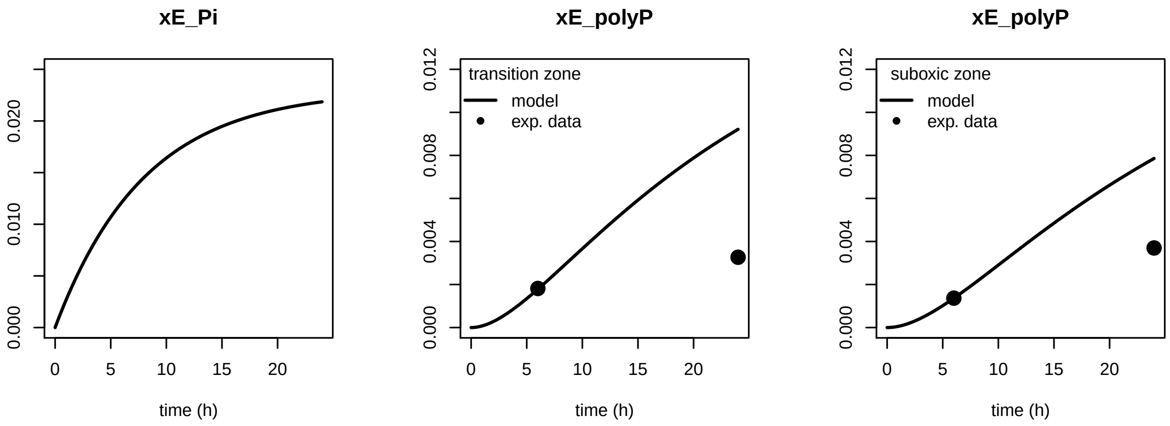

Figure 1: Results of the model scenario 1.

```
# Scenario 2: synthesis, no activity

define_pars.2  <- function(zone=1){
  r            <- Otot.ini/2.4/tmax/1
  fac          <- c(1.05, 3.48, 2.4)
  soff_factors <- c(7, 9, 12)
  pars <- c(r_synth = r*fac[zone],
            s.off   = soff_factors[zone]
            )
  return(pars)
}

# define model function
O18model.2 <-function(t, state, pars) {
  with (as.list(c(state, pars)),{

  x   <- O18/Otot                         # 18O atom fraction, poly-P pool
  xPi <- x.ini + (xS-x.ini)*(1-exp(-k*t)) # 18O atom fraction, Pi pool

  # THIS IS THE KEY DIFFERENCE BETWEEN MODEL SCENARIOS 1 & 2
  dO.dt   <- ifelse(t<s.off, r_synth,     0)
  dO18.dt <- ifelse(t<s.off, r_synth*xPi, 0)

  list(c(dO.dt, dO18.dt),
      xE_Pi    = xPi - x.ini,     # excess 18O, Pi pool (source)
      xE_polyP = O18/Otot - x.ini # excess 18O, poly-P
      )
  })
}

# solve the model

# transition zone
pars2 <- define_pars.2(zone=2)
out2  <- ode(y = yini, parms = pars2, func = O18model.2, times = outtimes)
```

```r
# suboxic zone
pars3 <- define_pars.2(zone=3)
out3  <- ode(y = yini, parms = pars3, func = O18model.2, times = outtimes)
```

```r
# plot model results
plot(out2,
     which=c("Otot", "xE_polyP"),
       xlab="time (h)", lty=1, lwd=2, mfrow=c(1,4),
       obs = DATA2, obspar = list(pch=16, col=1, cex=2),
       ylim = list(c(0,2), c(0,0.012)))
legend("topleft", legend = c("model", "exp. data"),
       title="transition zone", col=1, lwd=2, lty=c(1,NA),
       pch=c(NA,16), bty="n")
plot(out3,
     which=c("Otot", "xE_polyP"),
       xlab="time (h)", lty=1, lwd=2, mfrow=NULL,
       obs = DATA3, obspar = list(pch=16, col=1, cex=2),
       ylim = list(c(0,2), c(0,0.012)))
legend("topleft", legend = c("model", "exp. data"),
       title="suboxic zone", col=1, lwd=2, lty=c(1,NA),
       pch=c(NA,16), bty="n")
```

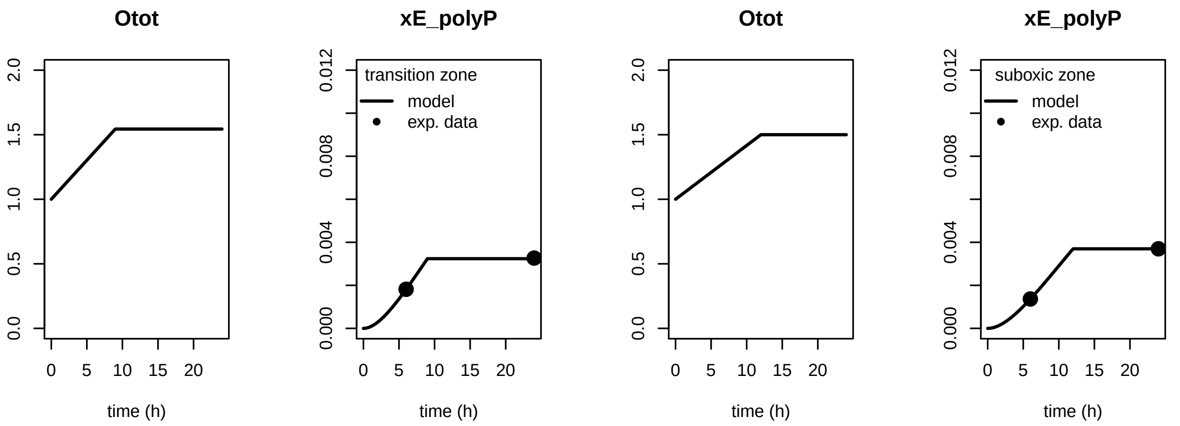

Figure 2: Results of the model scenario 2.

Scenario 3

As another more realistic scenario, we assume that, during the 24 h incubation, there was first a phase of poly-P synthesis, followed by a phase of poly-P breakdown, followed by another phase of poly-P synthesis. Additionally, we assume that the rate of poly-P synthesis during the first and second phase were equal, and the rate of poly-P breakdown was the same as the rate of poly-P synthesis ($r_{break} = -r_{synth}$). Similar to scenario 2, we adjust the duration of the synthesis and breakdown phases such that the excess ^{18}O atom fraction predicted by the model after 6 h and 24 h matches the experimentally determined values. Note that we only show *one* possible choice of model parameters for which this match between the experimental and model values can be achieved.

```r
# Scenario 3: synthesis, breakdown, synthesis

define_pars.3 <- function(zone=1){
  r            <- Otot.ini/2.4/tmax/1
  fac          <- c(1.05, 3.48, 2.4)
  soff_factors <- c(6, 6, 7)
  son_factors  <- c(21.6, 23.65, 22.3)
  pars <- c(r_synth = r*fac[zone],
            s.off   = soff_factors[zone],
            s.on    = son_factors[zone]
            )
  return(pars)
}

# define model function
O18model.3 <-function(t, state, pars) {
  with (as.list(c(state, pars)),{

  x   <- O18/Otot                          # 18O atom fraction, poly-P pool
  xPi <- x.ini + (xS-x.ini)*(1-exp(-k*t)) # 18O atom fraction, Pi pool

  # THIS IS THE KEY DIFFERENCE BETWEEN MODEL SCENARIOS 2 & 3
  dO.dt   <- ifelse(t<s.off | t>s.on, r_synth,       -r_synth)
  dO18.dt <- ifelse(t<s.off | t>s.on, r_synth*xPi, -x*r_synth)

  list(c(dO.dt, dO18.dt),
      xE_Pi    = xPi      - x.ini, # excess 18O, Pi pool (source)
      xE_polyP = O18/Otot - x.ini  # excess 18O, poly-P
      )
  })
}

# solve the model

# transition zone
pars2 <- define_pars.3(zone=2)
out2  <- ode(y = yini, parms = pars2, func = O18model.3, times = outtimes)
# suboxic zone
pars3 <- define_pars.3(zone=3)
out3  <- ode(y = yini, parms = pars3, func = O18model.3, times = outtimes)
```

```r
# plot model results
plot(out2,  which=c("Otot", "xE_polyP"),
       xlab="time (h)", lty=1, lwd=2, mfrow=c(1,4),
       obs = DATA2, obspar = list(pch=16, col=1, cex=2),
       ylim = list(c(0,2), c(0,0.012)))
legend("topleft", legend = c("model", "exp. data"),
       title="transition zone", col=1, lwd=2, lty=c(1,NA),
       pch=c(NA,16), bty="n")
plot(out3,  which=c("Otot", "xE_polyP"),
       xlab="time (h)", lty=1, lwd=2, mfrow=NULL,
       obs = DATA3, obspar = list(pch=16, col=1, cex=2),
       ylim = list(c(0,2), c(0,0.012)))
```

```
legend("topleft", legend = c("model", "exp. data"),
       title="suboxic zone", col=1, lwd=2, lty=c(1,NA),
       pch=c(NA,16), bty="n")
```

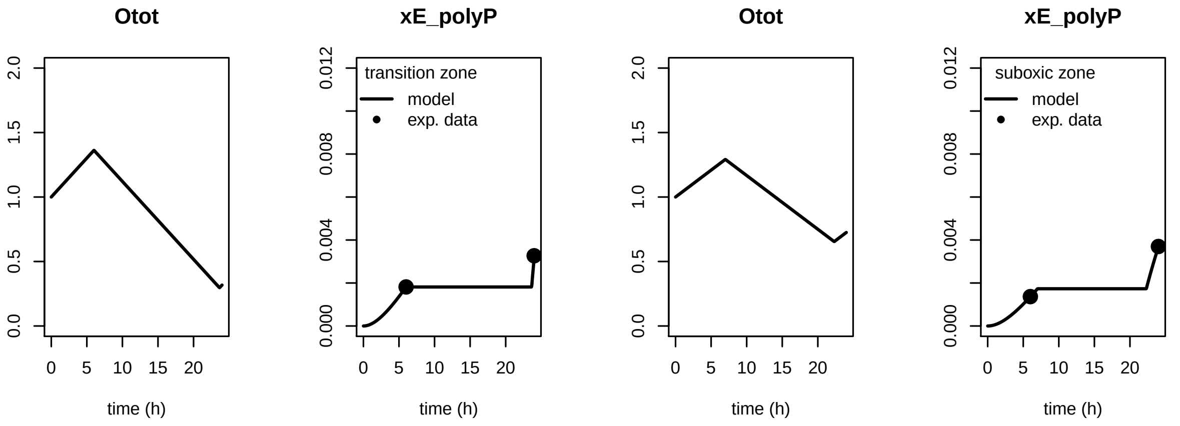

Figure 3: Results of the model scenario 3.

Conclusion

As illustrated by Figure 1, it is unlikely that poly-P was synthesized and incorporated into individual poly-P granules at a constant rate during the *entire* 24 h incubation period. More realistic scenarios involve at least one phase of inactivity or poly-P breakdown during the 24 h incubation period.

References

Blake, R. E., O'Neil, J. R., and Surkov, A. V. (2005). Biogeochemical cycling of phosphorus: Insights from oxygen isotope effects of phosphoenzymes. Am. J. Sci. 305, 596–620. doi:10.2475/ajs.305.6-8.596.

Polerecky, L., Eichner, M., Masuda, T., Zavřel, T., Rabouille, S., Campbell D. A., Halsey, K. (2022). Calculation and Interpretation of Substrate Assimilation Rates in Microbial Cells Based on Isotopic Composition Data Obtained by nanoSIMS. Frontiers in Microbiology, DOI 10.3389/fmicb.2021.621634

R Core Team (2020). R: A language and environment for statistical computing. R Foundation for Statistical Computing, Vienna, Austria. URL https://www.R-project.org/.

Soetaert, K., Petzoldt, T., Woodrow Setzer, R. (2010). Solving Differential Equations in R: Package `deSolve`. Journal of Statistical Software, 33(9), 1–25. URL http://www.jstatsoft.org/v33/i09/ DOI 10.18637/jss.v033.i09
