# Supplementary material for: Polyphosphate Dynamics in Cable Bacteria
Source: Front Microbiol. 2022 May 19;13:883807. doi: 10.3389/fmicb.2022.883807 (PMC9159916; doi:10.3389/fmicb.2022.883807)
Supplement: Supplementary file 6 [file Presentation_2.PDF]

The role of polyphosphates in the metabolism of multicellular cable bacteria

Supplementary Information

Contents

1 Linear mixed modelling **1**

2 Model 1 - Excess ^{18}O atom fraction as a function of redox zonation and labelling **1**

2.1 Data exploration 1

2.2 The linear mixed model 6

2.3 The final model 13

3 Model 2 - Excess ^{13}C atom fraction as a function of redox zonation and labelling **15**

3.1 Data exploration 15

3.2 The beyond optimal model 17

3.3 The final model 24

References **25**

1 Linear mixed modelling

Two linear mixed models were built to assess the variation between the redox zones and the labelling period. The first models focuses on the excess ^{18}O atom fraction within poly-P granules between the different redox zones and the labelling periods (model 1) whereas the second model focuses on the variation in the excess ^{13}C atom fraction within cells between the redox zones and the labelling periods (model 2).

To assess the significance of the labelling period and the redox zonation, both on the excess ^{18}O atom fraction within a polyphosphate granule and the excess ^{13}C atom fraction within the cytoplasm, a linear mixed model was built using the R package nlme (Pinheiro, Bates, and R-core 2022). Here we outline the step-by-step selection process for both models, the resulting figures and the code whereas the biological significance of this data will be discussed in the main text. For both models, we start with the data exploration followed by the actual analysis of the data using a step-up approach (Zuur et al. 2009).

2 Model 1 - Excess ^{18}O atom fraction as a function of redox zonation and labelling

Before data exploration and analysis, each polyphosphate granules measured was assigned to a specific cell (855 levels) within a specific filament (196 levels), as well as a specific core (4 levels), zone (3 levels) and

labelling period (2 levels). Because all the possible explanatory variables are nominal they were defined as factors.

2.1 Data exploration

2.1.1 Check for outliers

The first step is to check for outliers. To this end, a cleveland dotplot was produced. Such a plot is also useful to get an indication of the spread of the data. Because our data is nested (polyphosphate granules within cells within filaments within cores), and there are 855 unique cells from 196 unique filaments measured with a variable number of measurements per cell and/or filament, a cleveland dotplot is easier to interpret than a boxplot. Two cleveland dotplots were made where each row of dots is a filament (figure 1A) and where each group of dots reflect all the measurements within a core (figure 1B).

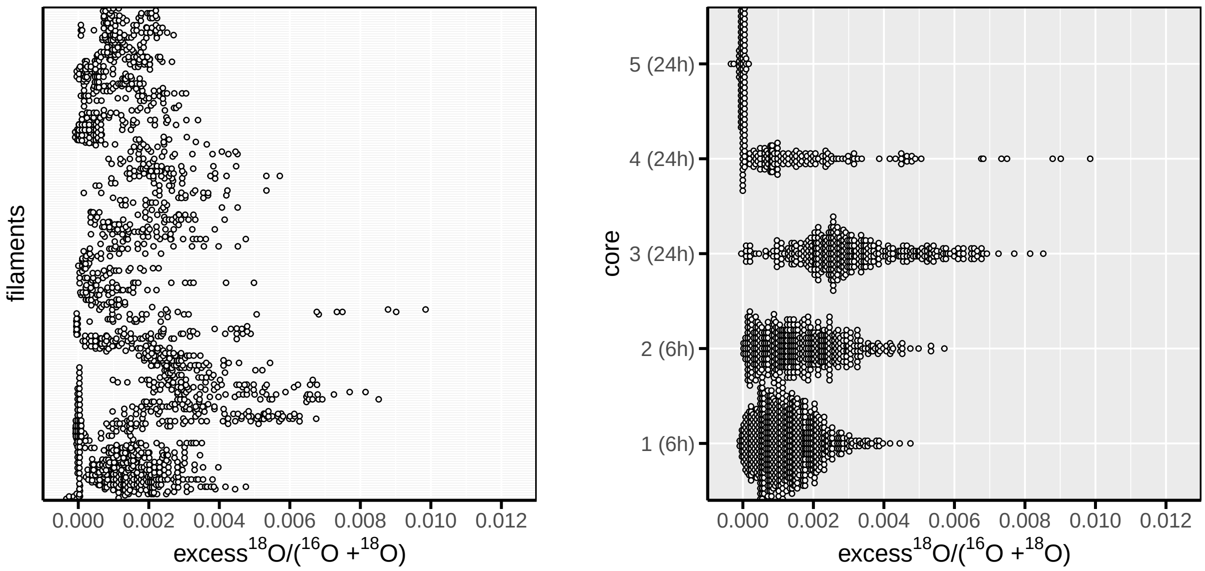

Figure 1: Cleveland dot plots grouped (A) per filament and (B) per core

No outliers were observed, so none of the data points was removed before the statistical analysis. The dotplots show a different spread between the different cores which appears to correspond with the labelling period; the 24h incubations (core 3 & core 4) seem to show a larger spread and hint towards heteroscedacity (unequal variances).

2.1.2 Homogeneity of variance

The next step in the data exploration is to assess whether or not the variance is homogeneous. This is best assessed with the use of boxplots. Boxplots were made were the excess ^{18}O atom fraction was grouped per redox zone within each core. Since two of the cores are biological replicates, the cores were grouped according to their labelling period (figure 2).

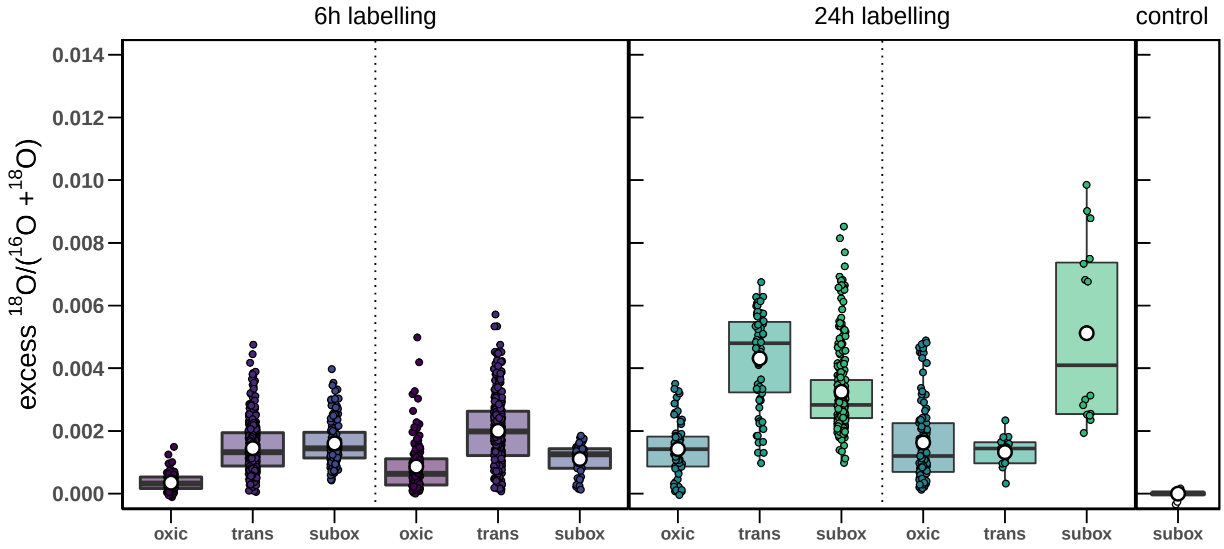

Figure 2: Boxplots of the excess ^{18}O atom fraction in (A) the 6h labelling period and (B) the 24h labelling period. Each data point represents the excess value calculated within a polyphosphate granule. White circles and horizontal lines show the mean and median ^{18}O atom fractions, respectively. Note that there is no grouping into cells and/or filaments, this is a depiction of the raw data.

The boxplots (Fig. 2) indicate that the variation is different between the three redox zones and the labelling periods. Boxplots of the fitted values versus the residuals (Fig. 3) values confirm this observation.

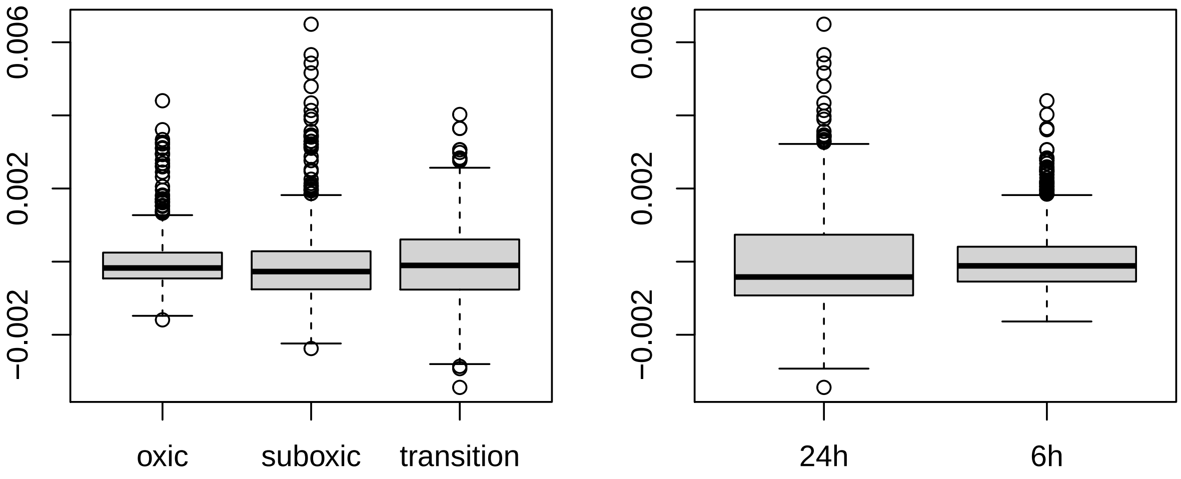

Figure 3: Boxplots of the fitted values versus the residuals grouped per (A) redox zone and (B) labelling period

In conclusion, variances are unequal which appears on the level of redox zonation (oxic, suboxic and transition) and labelling period (6h and 24h).

2.1.3 Normal Distribution

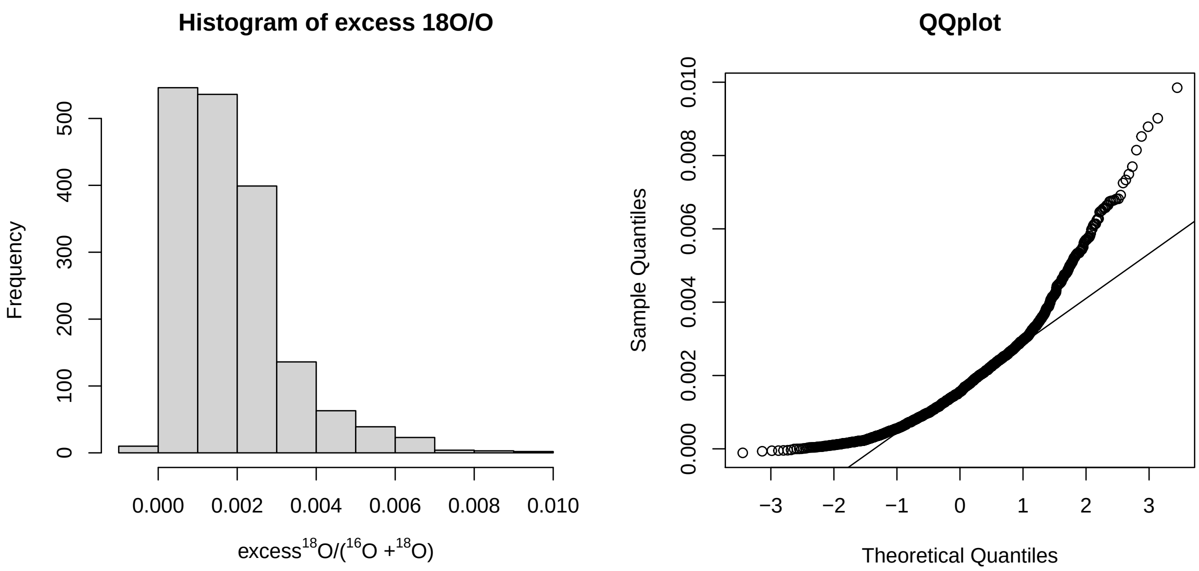

Figure 4: Histogram of (A) the excess ^{18}O atom fraction and (B) the corresponding qqplot. Both figures show that the data display a non-normal distribution

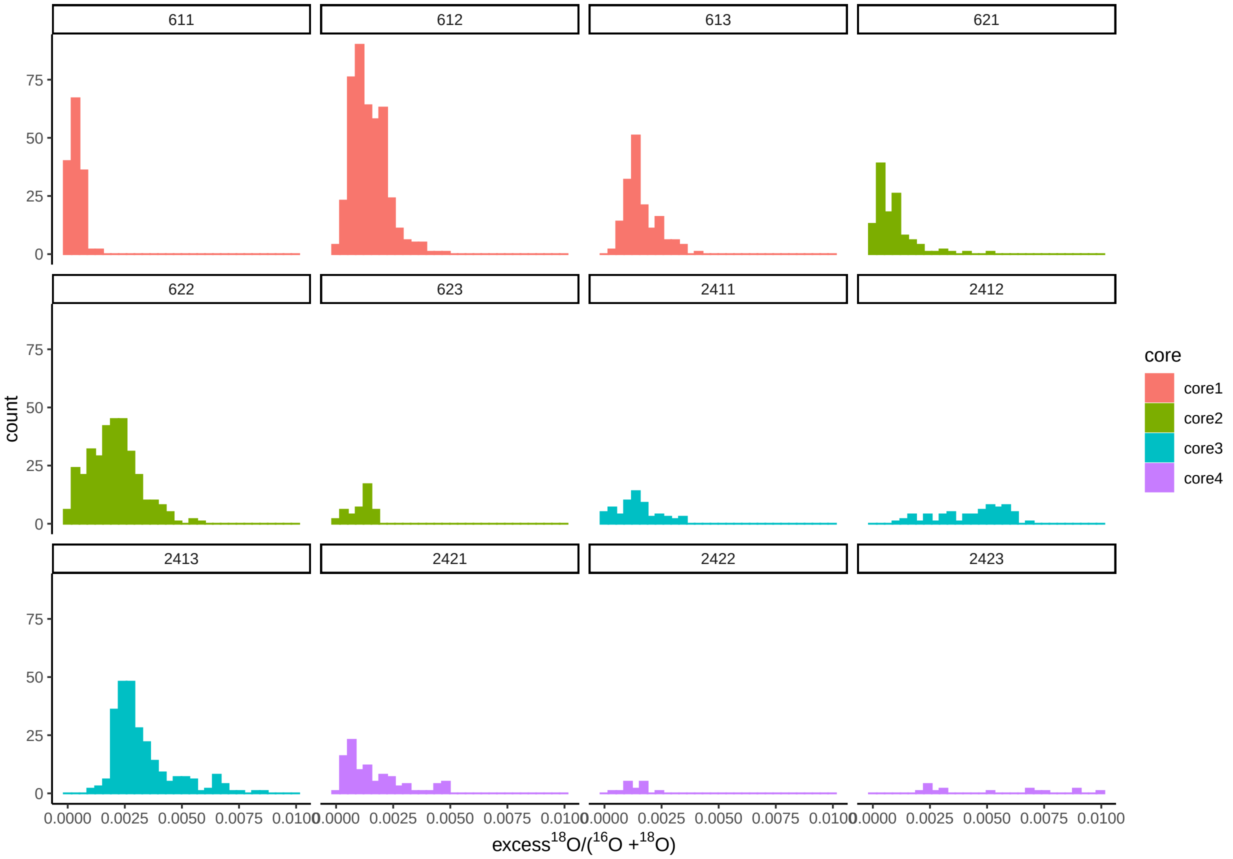

Figure 5: Histograms of the distributions split out per redox zones of each of the cores. The data displays a non-normal distribution

The data shows a non-normal distribution but because we are building a linear mixed model, this is not a problem (Zuur et al. 2009).

2.2 The linear mixed model

A linear mixed model includes both fixed and random components. To decide on the final model, including which components to include the step-up method as described by Zuur et al (2009) was used. First, a so-called beyond optimal model is built which includes as many fixed components and interactions as possible. By adapting this model the optimal random structure and the optimal variance structure will be determined. Once the optimal random structure and variance structure is determined, the importance of each of the fixed components and its interactions is determined. Models are compared using the Akaike Information criterium (AIC). The AIC measures the model fit and model complexity and quantifies the relative proximity to reality amongst different models. The lower the AIC, the better the fit of the model (Zuur et al. (2009)). The model selection for model 1, which is used to investigate the effect of the redox zonation and the labelling period on the polyphosphate metabolism, starts with the "beyond optimal model" that only includes included fixed effects, in this case redox zonation, labelling period and their interaction.

2.2.1 The beyond optimal model

A generalized least squares model is used as a basis for model comparison. Both the redox zonation and the labelling period as well as the interaction between the two are included as fixed components.

```
A0 <- gls(excess_18O ~ 1 + zone*labelling, data = df4, method = "REML")
```

Table 1: Outcome of model A0

	degrees of freedom	F-value	p-value
(Intercept)	1	5202	0
zone	2	260	0
labelling	1	652	0
zone:labelling	2	28	0

2.2.2 The ideal random structure

First the random structure is introduced. With the introduction of a random structure, the model becomes a linear mixed model. The random structure allows for variation between filaments and variation between cells of the same filament. Thus, it includes between-filament variation and between-cell variation within each filament.

Because each measurement on a polyphosphate granule comes from a specific cell that belongs to a specific filament fragment that was retrieved from a specific core, two random structures were defined:

1. polyphosphate granules are grouped into cells (855 levels) which are grouped into filaments (196 levels)
2. polyphosphate granules are grouped into cells, which are grouped into filaments, which are then grouped into cores.

The mixed models with the random structure are compared to the model without the random structure to assess whether between-filament and between-cell variation within a filament needs to be taken into account. This is done by comparing the Akaike Information Criteria (AIC) of the models.

```
B1 <- lme(excess_18O ~ 1 + zone*labelling, data = df4, method = "REML",
          random = ~ 1 | label/cell)

B2 <- lme(excess_18O ~ 1 + zone*labelling, data = df4, method = "REML",
          random = ~ 1 | core/label/cell)
```

Table 2: Akaike Information Criteria values and degrees of freedom from models A0, B1, and B2

	degrees of freedom	AIC
A0	7	-18974
B1	9	-20511
B2	10	-20511

The AIC is lowest value for model B2 (-20511) and model B1 (-20511) when compared to model A0 (-18974). To assess if three-level nesting into cells and filaments (B1) or four-level nesting into cells, filaments and cores (B2) significantly improves the model, both model B1 and B2 were compared to the model without random components.

Table 3: comparison between model A0 en B1

Model	df	AIC	BIC	logLik	Test	L.Ratio	p-value
1	7	-18974	-18935	9494			
2	9	-20511	-20462	10265	1 vs 2	1541.54552815069	0

Table 4: comparison between model A0 en B2

Model	df	AIC	BIC	logLik	Test	L.Ratio	p-value
1	7	-18974	-18935	9494			
2	10	-20511	-20457	10266	1 vs 2	1543.65990001973	0

Nesting into cells and filaments (B1) significantly improves the model (L.ratio=1542, df=2, $p<0.0001$) as does four-level nesting into cells, filaments and cores (L.ratio=1544, df=3, $p<0.0001$). To assess if the four-level nesting of filament into cores is necessary, another comparison was done between the model with nesting into cells and filament and the models with nesting into cells, filaments and cores.

Table 5: comparison between model B1 en B2

Model	df	AIC	BIC	logLik	Test	L.Ratio	p-value
1	9	-20511	-20462	10265			
2	10	-20511	-20457	10266	1 vs 2	2.114	0.146

The model where cells are nested in filaments who in turn are nested in cores is not significantly better than the model where the random term constitutes the nesting into cells which are nested into filaments, even when corrected for testing on the boundary (L=2.11, df=1, p=0.073). Therefore, nesting into cores is excluded in the random part of the model.

Table 6: outcome from model B1

	numDF	denDF	F-value	p-value
(Intercept)	1	906	636	0
zone	2	190	37	0
labelling	1	190	65	0
zone:labelling	2	190	5	0

2.2.3 The ideal variance structure

The boxplots (figure 2) indicated that the variance was unequal between the different zones and between the different labelling periods. Therefore, three competing models were built where the variance was allowed to vary as a function of these fixed variables:

1. Variance is variable between the three redox zones
2. Variance is variable between the two labelling periods
3. Variance is variable between each combination of redox zonation and labelling periods, which would allowed for six different variances.

Again, the model comparison is done using the Akaike information coefficient (AIC).

```
vs1 <- varIdent(form= ~ 1|zone)
vs2 <- varIdent(form = ~1|labelling)
vs3 <- varIdent(form = ~1|zone*labelling)

C1 <- lme(
  excess_18O ~ 1 + zone*labelling, data = df4, weights = vs1, method = "REML",
  random = ~ 1 | label/cell)

C2 <- lme(
  excess_18O ~ 1 + zone*labelling, data = df4, weights = vs2, method = "REML",
  random = ~ 1 | label/cell)

C3 <- lme(
  excess_18O ~ 1 + zone*labelling, data = df4, weights = vs3, method = "REML",
  random = ~ 1 | label/cell)
```

Table 7: comparison between model B1, C1, C2, and C3

	degrees of freedom	AIC
B1	9	-20511
C1	11	-20678
C2	10	-20663
C3	14	-20839

The AIC is lowest for the model that uses one variance term per combination of redox zone and labelling period (-20839). To check if the model is significantly improved, the model without a variance structure is compared with the multiple variance model with the lowest AIC (C3). The log likelihood value and the p-value are used to assess if the model is significantly improved.

Table 8: comparison between model B1 and C3

Model	df	AIC	BIC	logLik	Test	L.Ratio	p-value
1	9	-20511	-20462	10265			
2	14	-20839	-20762	10433	1 vs 2	337.6	0

The mixed model without a variance structure (B1) is significantly improved when a multiple variance structure is introduced (L.ratio = 338, df = 5, $p < 0.0001$). The p-value needs to be corrected because we are "testing on the boundary," but even then the difference between the two models is still significant.

2.2.4 The ideal fixed effect structure

Now that the best variance structure and the best random structure is found, we continue with model C3 to find the ideal fixed effects structure, i.e. find the optimal model in terms of the explanatory variables redox zonation and labelling period. Because we would like to compare likelihood ratio tests, we choose the "ML" method (Zuur et al. (2009)).

```
D0 <- lme(
  excess_180 ~ 1 + zone*labelling, data = df4, weights = vs3, method = "ML",
  random = ~ 1 | label/cell)
```

Table 9: outcome of model D0

	numDF	denDF	F-value	p-value
(Intercept)	1	906	662	0
zone	2	190	38	0
labelling	1	190	68	0
zone:labelling	2	190	5	0

To assess which fixed structure is best, first the least significant term is removed. Then each term is subsequently dropped and all models are compared to the model with all fixed effects. The interaction term between zone and labelling (zone:labelling) has the least significance so this term is removed first. Subsequently, zone and label are removed as fixed components of the model.

```
D1 <- lme(
  excess_180 ~ 1 + zone + labelling, data = df4, weights = vs3, method = "ML",
  random = ~ 1 | label/cell)
D2 <- lme(
  excess_180 ~ 1 + zone, data = df4, weights = vs3, method = "ML",
  random = ~ 1 | label/cell)
D3 <- lme(
  excess_180 ~ 1 + labelling, data = df4, weights = vs3, method = "ML",
  random = ~ 1 | label/cell)
```

Table 10: comparison between model D0, D1, D2, and D3

	degrees of freedom	AIC
D0	14	-20930
D1	12	-20924
D2	11	-20870
D3	10	-20872

The model where both the redox zonation, the labelling period and and interaction term between the components is included has the lowest AIC (-20930). To assess if each term is significant, the model with all fixed terms included (D0) is compared to the model without the interaction term (D1), the model without the labelling period (D2), and the model without redox zonation (D3).

Table 11: comparison between model D0 and D1

Model	df	AIC	BIC	logLik	Test	L.Ratio	p-value
1	14	-20930	-20853	10479			
2	12	-20924	-20858	10474	1 vs 2	9.5	0.009

Table 12: comparison between model D0 and D2

Model	df	AIC	BIC	logLik	Test	L.Ratio	p-value
1	14	-20930	-20853	10479			
2	11	-20870	-20810	10446	1 vs 2	65.6	0

Table 13: comparison between model D0 and D3

Model	df	AIC	BIC	logLik	Test	L.Ratio	p-value
1	14	-20930	-20853	10479			
2	10	-20872	-20818	10446	1 vs 2	65.3	0

Testing showed that each of the terms is significant and thus no terms can be removed from the model. Therefore the final model is refitted with REML to check if the variances are equal and thus if the model is valid.

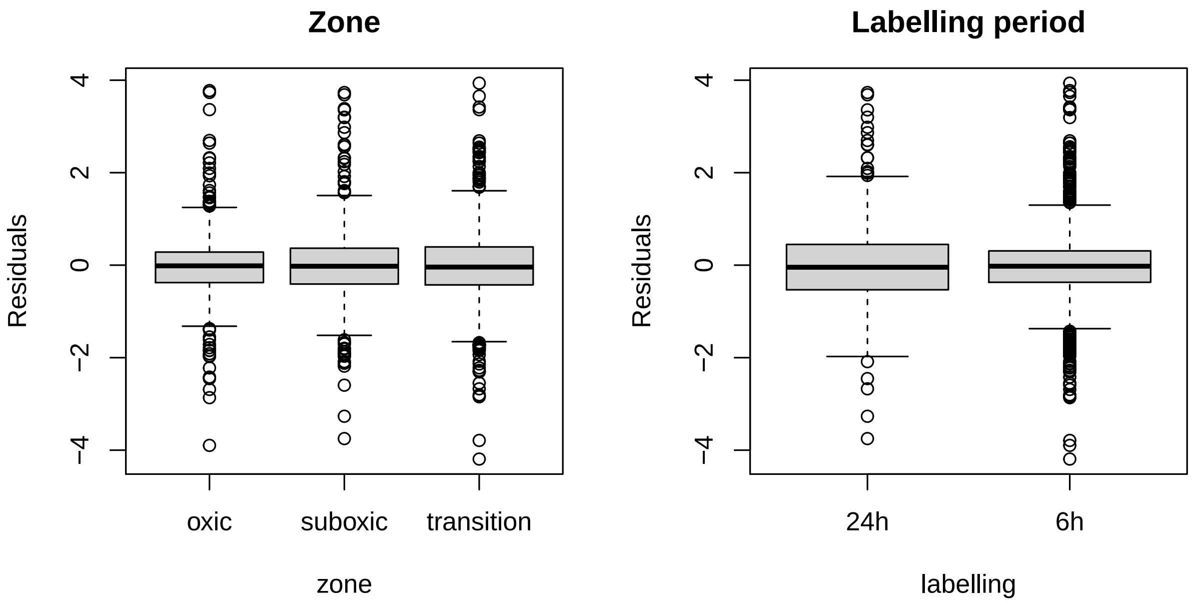

Figure 6: Residuals of the final model as a function of redox zonation and labelling period

Inspection of the residuals shows that the variances are now equal and the model is thus valid. Therefore, the best linear mixed model includes the fixed effect “zone” and “labelling period” as well as their interaction, has a random structure where poly-P granules are grouped into cells which are grouped into filaments and the variance is allowed to vary between each combination of zone and labelling period (i.e. a random variance structure).

This model is used to investigate the effect of the redox zonation and the labelling period on the excess ^{18}O atom fraction of a polyphosphate as well as the variation within and between filaments.

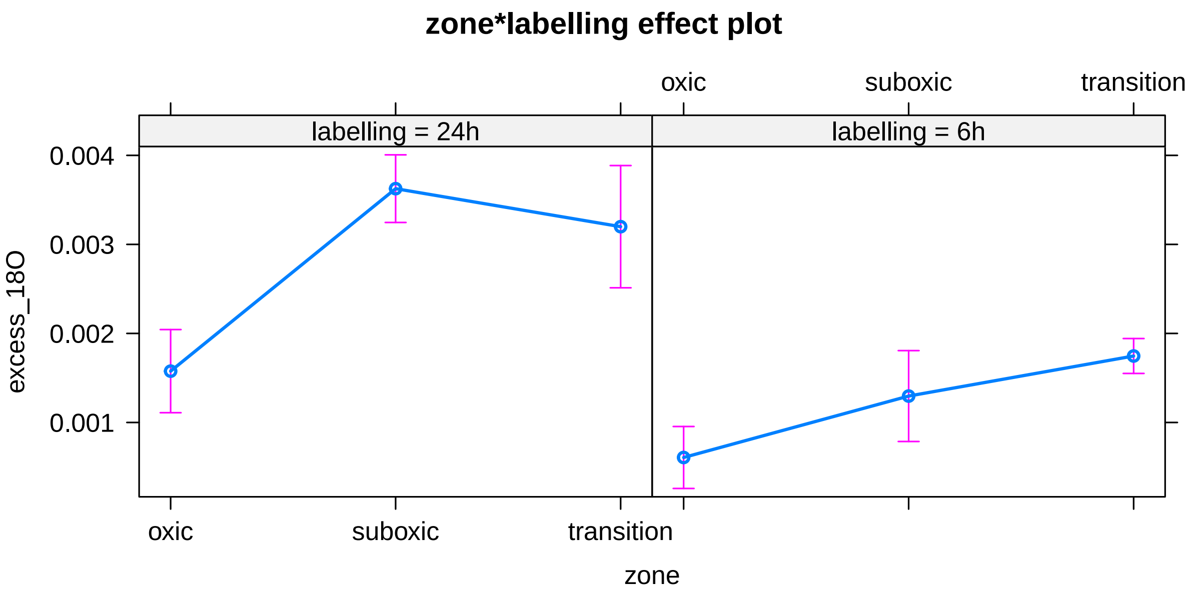

Figure 7: Effect plot showing the modelled values of each combination of redox zonation and labelling period

Table 14: outcome of model D0 with the oxic zone 6h labelling as a reference

effect	group	term	estimate	std.error	df	statistic	p.value
fixed	fixed	(Intercept)	0.00061	0.00018	906	3.36085	0.00081
fixed	fixed	zonesuboxic	0.00069	0.00032	190	2.15331	0.03255
fixed	fixed	zonetransition	0.00114	0.00021	190	5.50336	0.00000
fixed	fixed	labelling24h	0.00097	0.00030	190	3.21078	0.00155
fixed	fixed	zonesuboxic:labelling24h	0.00136	0.00045	190	3.04558	0.00265
fixed	fixed	zonetransition:labelling24h	0.00048	0.00048	190	1.00723	0.31511

The baseline for this model is the 6h labelling period from the oxic zone. Releveling of the different combinations of redox zones and labelling periods was done to assess if the modeled differences between biologically relevant combinations (e.g. 6h oxic vs. 6h suboxic) are significant.

Table 15: outcome of model D0 with the transition zone 6h labelling as a reference

effect	group	term	estimate	std.error	df	statistic	p.value
fixed	fixed	(Intercept)	0.00175	0.00010	906	17.18655	0.00000
fixed	fixed	zoneoxic	-0.00114	0.00021	190	-5.50336	0.00000
fixed	fixed	zonesuboxic	-0.00045	0.00028	190	-1.58763	0.11403
fixed	fixed	labelling24h	0.00145	0.00037	190	3.91990	0.00012
fixed	fixed	zoneoxic:labelling24h	-0.00048	0.00048	190	-1.00723	0.31511
fixed	fixed	zonesuboxic:labelling24h	0.00088	0.00050	190	1.77679	0.07720

Table 16: outcome of model D0 with the suboxic zone 6h labelling as a reference

effect	group	term	estimate	std.error	df	statistic	p.value
fixed	fixed	(Intercept)	0.00130	0.00026	906	4.89829	0.00000
fixed	fixed	zonetransition	0.00045	0.00028	190	1.58763	0.11403
fixed	fixed	zoneoxic	-0.00069	0.00032	190	-2.15331	0.03255
fixed	fixed	labelling24h	0.00233	0.00033	190	7.06946	0.00000
fixed	fixed	zonetransition:labelling24h	-0.00088	0.00050	190	-1.77679	0.07720
fixed	fixed	zoneoxic:labelling24h	-0.00136	0.00045	190	-3.04558	0.00265

Table 17: outcome of model D0 with the oxic zone 24h labelling as a reference

effect	group	term	estimate	std.error	df	statistic	p.value
fixed	fixed	(Intercept)	0.00158	0.00024	906	6.51146	0.00000
fixed	fixed	zonesuboxic	0.00205	0.00031	190	6.57808	0.00000
fixed	fixed	zonetransition	0.00162	0.00043	190	3.76690	0.00022
fixed	fixed	labelling6h	-0.00097	0.00030	190	-3.21078	0.00155
fixed	fixed	zonesuboxic:labelling6h	-0.00136	0.00045	190	-3.04558	0.00265
fixed	fixed	zonetransition:labelling6h	-0.00048	0.00048	190	-1.00723	0.31511

Table 18: outcome of model D0 with the transition zone 24h labelling as a reference

effect	group	term	estimate	std.error	df	statistic	p.value
fixed	fixed	(Intercept)	0.00320	0.00036	906	8.98552	0.00000
fixed	fixed	zoneoxic	-0.00162	0.00043	190	-3.76690	0.00022
fixed	fixed	zonesuboxic	0.00043	0.00041	190	1.05899	0.29095
fixed	fixed	labelling6h	-0.00145	0.00037	190	-3.91990	0.00012
fixed	fixed	zoneoxic:labelling6h	0.00048	0.00048	190	1.00723	0.31511
fixed	fixed	zonesuboxic:labelling6h	-0.00088	0.00050	190	-1.77679	0.07720

2.3 The final model

The optimal model includes the fixed parameters redox zonation, labelling period and their interaction. Thus, the final model includes these three fixed terms, two random components where poly-P are nested into cells which are nested into filaments and a variable variance structure:

$$excess\left(\frac{^{18}O}{^{16}O+^{18}O}\right)_{ijk} = \alpha+(\beta_1\times zone_{ijk}+\beta_2\times labelling\ period_{ijk}+\beta_3\times zone_{ijk}\times labelling\ period_{ijk})+a_j+a_{j|k}+\epsilon_{ijk}$$

This is a random intercept model where the average intercept is denoted by the term α. How the intercept varies as a function of the filament and cell is denoted in the term a_j and $a_{j|k}$, respectively, where the index j denotes the filament and the index k denotes the cell. Both a_j and $a_{j|k}$ are normally distributed with $N(0, \sigma_j^2)$ and $N(0, \sigma_{j|k}^2)$, respectively. The term ϵ_{ijk} is the unexplained error and because a multiple variance structure was added this value is different for each combination of the explanatory variables zone and labelling period. Thus, $\epsilon_{ijk} \sim N(0, \sigma_m^2)$ where m denotes the different values for the variances for each

of the six combinations of redox zone and labelling period (e.g. oxic zone 6h labelling). The output from model 1 gives values of the modelled excess ^{18}O values for each combination of labelling period and zone ($\beta_1 \times zone_{ijk} + \beta_2 \times labelling\ period_{ijk} + \beta_3 \times zone_{ijk} \times labelling\ period_{ijk}$).

The model output shows that the random effect a_j, representing the between filament variation is $N(0, 0.0009713^2)$, the random effect $a_{j|k}$, representing the variation between cells of the same filament, is $N(0, 0.0002857^2)$. The random noise (ϵ_{ijk}) is variable and dependent on the combination of redox zonation and labelling period. These values can be used to calculate the intraclass correlation at both the filament level ($ICC_{filament}$) and at the cell level (ICC_{cell}). The formulae are as follows:

$$ICC_{filament} = \frac{\sigma^2_{filament}}{\sigma^2_{filament} + \sigma^2_{cell} + \sigma^2}$$

$$ICC_{cell} = \frac{\sigma^2_{filament} + \sigma^2_{cell}}{\sigma^2_{filament} + \sigma^2_{cell} + \sigma^2}$$

Because σ^2 varies per zone and labelling period, the ICC is different for each combination of zone and labelling period.

	zone	σ^2	ICC_{cell}	$ICC_{filament}$
6h	oxic	0.000207	0.960	0.884
6h	transition	0.000485	0.813	0.749
6h	suboxic	0.000429	0.848	0.780
24h	oxic	0.000462	0.827	0.762
24h	transition	0.000624	0.725	0.667
24h	suboxic	0.000899	0.559	0.515

3 Model 2 - Excess ^{13}C atom fraction as a function of redox zonation and labelling

To assess the significance of the labelling period and the the redox zonation on the excess ^{13}C atom fraction within the cell, a second linear mixed model was built using the R package nlme. The step-by-step selection process is similar to the selection process described for model 1. A description of the model and a discussion of the biological relevance is described in the main text.

Before data exploration, each cell from which the excess ^{13}C atom fraction was measured (704) was assigned to a specific filament (164), as well as a specific core (4 levels), zone (3 levels) and labelling period (2 levels). Because all the possible explanatory variables are nominal they were defined as factors.

3.1 Data exploration

3.1.1 Check for outliers

The first step is to check for outliers. To this end, another cleveland dotplot was produced. Because our data is nested (cells within filaments within cores), and there are 704 unique cells from 164 unique filaments measured with a variable number of measurements per filament, a Cleveland dotplot is easier to interpret than a boxplot. Two cleveland dotplots were made where each row of dots is a filament (figure 1A) and where each group of dots reflect all the measurements within a core (figure 1B).

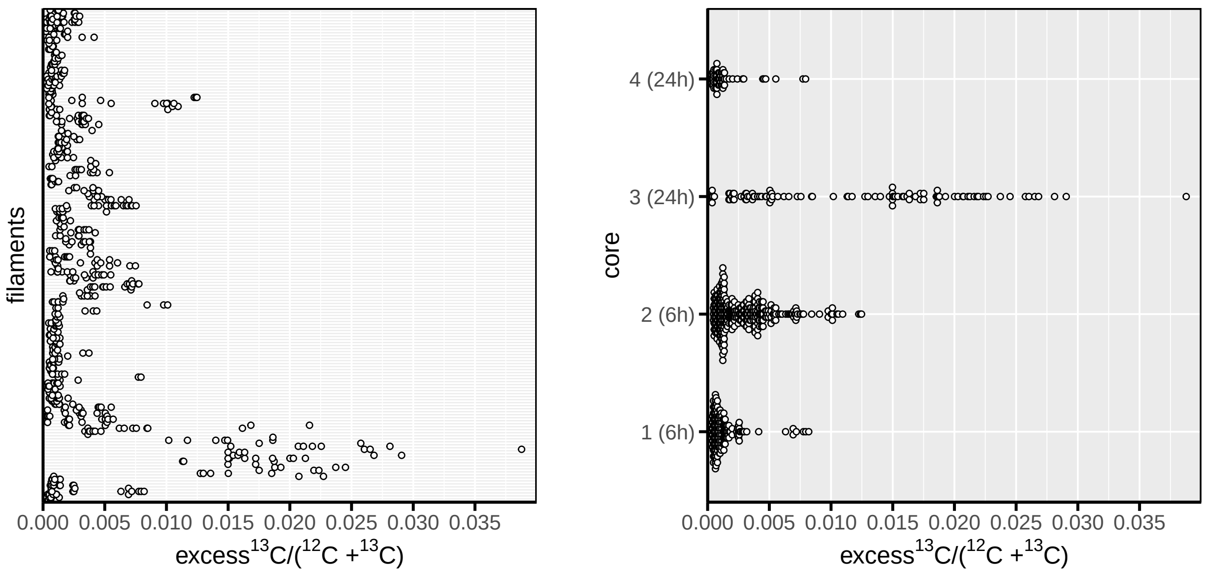

Figure 8: Cleveland dot plots grouped (A) per filament and (B) per core

No outliers were observed, so no data points were removed before the analysis. The dotplots show a different spread between the different cores which appears to correspond with the labelling period; the 24h incubations (core 3 & core 4) seem to show a larger spread and hint towards heteroscedacity (unequal variances).

3.1.2 Homogeneity of variance

The next step in the data exploration is to assess whether or not the variance is homogeneous. This is best assessed with the use of boxplots. Boxplots were made were the excess ^{13}C atom fraction was grouped per redox zone within each core. Since two of the cores are biological replicates, the cores were grouped according to their labelling period (figure 9).

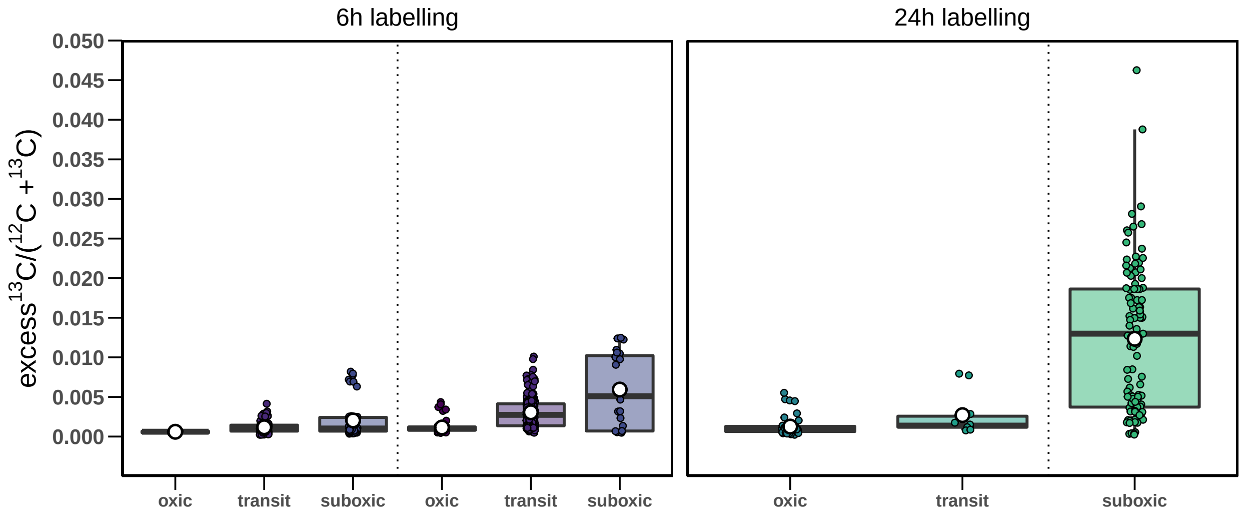

Figure 9: Boxplots of the excess ^{13}C atom fraction in (A) the 6h labelling period and (B) the 24h labelling period. Each data point represents the value calculated within a cell. White circles and horizontal lines show the mean and median excess ^{13}C atom fractions, respectively. Note that there is no grouping into filaments, this is a depiction of the raw data.

The boxplots (Fig. 9) indicate that the variation is different and much more pronounced in the suboxic zone. Boxplots of the fitted values versus the residuals (Fig. 10) values confirm this observation.

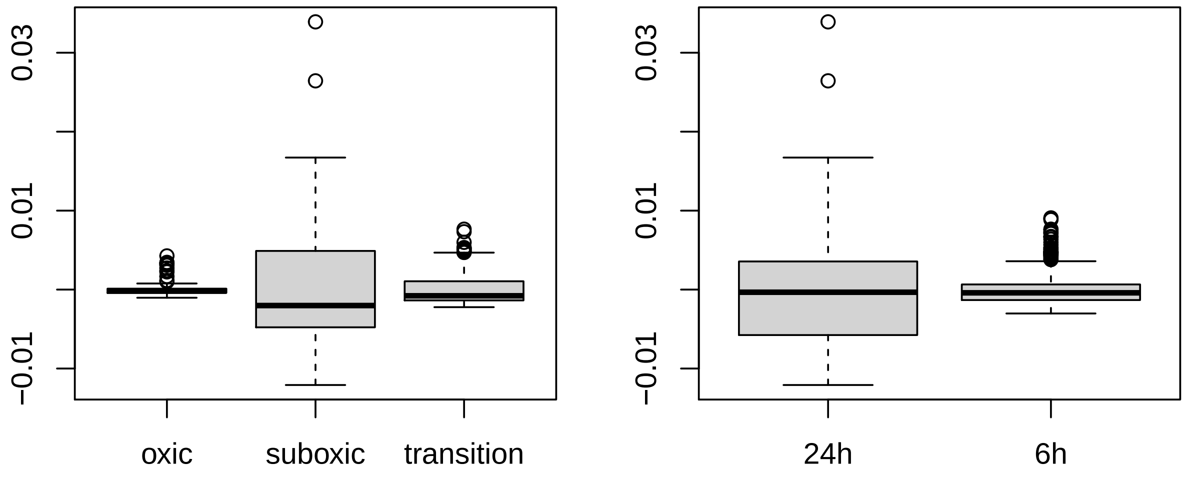

Figure 10: Boxplots of the fitted values versus the residuals grouped per (A) redox zone and (B) labelling period

Thus, for the excess ^{13}C atom fraction, variances are unequal which appears on the level of redox zonation (oxic, suboxic and transition) and labelling period (6h and 24h).

3.2 The beyond optimal model

A generalized least squares model is used as a basis. Both the redox zonation, the labelling period as well as the interactions between the two are included.

```
E0 <- gls(mean ~ 1 + zone*labelling, data = dfC2, method = "REML")
```

Table 19: outcome of model E0

	degrees of freedom	F-value	p-value
(Intercept)	1	589	0
zone	2	188	0
labelling	1	106	0
zone:labelling	2	51	0

3.2.1 The ideal random structure

The next step in the model selection is to assess which random structure is best. We compare different model with a random structure with the model without a random structure to assess the best random structure for our data. Because the measurement within a cell belong to a specific filament fragment that was retrieved from a specific core, two random structures were defined:

1. Cells (704) are grouped into filaments (164 levels)
2. Cells are grouped into filaments, which are then grouped into cores.

```r
F1 <- lme(mean ~ 1 + zone*labelling, data = dfC2, method = "REML",
          random = ~ 1 | label)
F2 <- lme(mean ~ 1 + zone*labelling, data = dfC2, method = "REML",
          random = ~ 1 | core/label)
```

Table 20: Akaike Information Criteria values and degrees of freedom from models E0, F1, and F2

	degrees of freedom	AIC
E0	7	-5693
F1	8	-6721
F2	9	-6721

The AIC is lowest value for model F1 and F2 (both -6721) when compared to model E0 (-5693). To assess if nesting into filaments (F1) or nesting into filaments and cores (F2) significantly improves the model, these model were compared to the model without random components and to one another.

Table 21: comparison between model E0 and F1

Model	df	AIC	BIC	logLik	Test	L.Ratio	p-value
1	7	-5693	-5661	2853			
2	8	-6721	-6684	3368	1 vs 2	1030.2	0

Table 22: comparison between model F1 and F2

Model	df	AIC	BIC	logLik	Test	L.Ratio	p-value
1	8	-6721	-6684	3368			
2	9	-6721	-6680	3369	1 vs 2	2	0.156

Three-way level nesting of cells into filaments and filaments into cores (F2) does not significantly improve the model when compared to a model where cells are nested into filaments (F1). Two-way level nesting of cells into filaments (F1) significantly improves the original model (E0, L.ratio=1030, df=1, $p<0.0001$). Thus, the model with the random component is used for further analysis.

3.2.2 The ideal variance structure

Because variances are unequal and appear to differ between the different zones and labelling periods. Therefore, three competing models were built where the variance was allowed to vary as a function of the fixed variables:

1. Variance is variable between the three redox zones
2. Variance is variable between the two labelleling periods
3. Variance is variable between each combination of redox zonation and labelling periods, which would allowed for six different variances.

```r
vs1 <- varIdent(form= ~ 1|zone)
vs2 <- varIdent(form = ~1|labelling)
vs3 <- varIdent(form = ~1|zone*labelling)

G1 <- lme(mean ~ 1 + zone*labelling, data = dfC2, weights = vs1,
          random = ~ 1 | label, method = "REML")
G2 <- lme(mean ~ 1 + zone*labelling, data = dfC2, weights = vs2,
          random = ~ 1 | label, method = "REML")
G3 <- lme(mean ~ 1 + zone*labelling, data = dfC2, weights = vs3,
          random = ~ 1 | label,method = "REML")
```

Table 23: Akaike Information Criteria values and degrees of freedom from models E0, F1, and F2

	degrees of freedom	AIC
F1	8	-6721
G1	10	-7387
G2	9	-7277
G3	13	-7549

The AIC is lowest for the model that uses one variance term per combination of redox zone and labelling period (-7549). To check if the model is significantly improved, the model without a variance structure is compared with this multiple variance model (G3). The log likelihood value and the p-value are used to assess if the model is significantly improved.

Table 24: comparison between model F1 and G3

Model	df	AIC	BIC	logLik	Test	L.Ratio	p-value
1	8	-6721	-6684	3368			
2	13	-7549	-7490	3788	1 vs 2	838.4	0

The mixed model without a variance structure (F1) is significantly improved when a multiple variance structure is introduced (L.ratio = 838, df = 5, $p < 0.0001$). The p-value needs to be corrected because we are "testing on the boundary," but even then the difference between the two models is still significant.

Now that the best variance structure and the best random structure is found, we continue with model G3 to find the ideal fixed effects structure, i.e. find the optimal model in terms of the explanatory variables redox zonation and labelling period. Because we would like to compare likelihood ratio tests, we choose the "ML" method.

```r
H0 <- lme(mean ~ 1 + zone*labelling, data = dfC2, weights = vs3, method = "ML",
          random = ~ 1 | label)
```

Table 25: outcome of model H0

	numDF	denDF	F-value	p-value
(Intercept)	1	540	212.6338	0
zone	2	158	42.4453	0
labelling	1	158	31.9180	0
zone:labelling	2	158	24.7114	0

3.2.3 The ideal fixed effect structure

To assess which fixed structure is best, first the least significant term is removed. Then each term is subsequently dropped and all models are compared to the model with all fixed effects.

```
H1 <- lme(
  mean ~ 1 + zone + labelling, data = dfC2, weights = vs3, method = "ML",
  random = ~ 1 | label)
H2 <- lme(
  mean ~ 1 + zone, data = dfC2, weights = vs3, method = "ML",
  random = ~ 1 | label)
H3 <- lme(
  mean ~ 1 + labelling, data = dfC2, weights = vs3, method = "ML",
  random = ~ 1 | label)
```

Table 26: Akaike Information Criteria values and degrees of freedom from models H0, H1, H2 and H3

	degrees of freedom	AIC
H0	13	-7627
H1	11	-7586
H2	10	-7567
H3	9	-7535

The AIC is lowest for the model that includes all fixed effects and their interactions (H0). To assess if this model is significantly better, it is compared to the model that exclude the interaction term (H1), the model that does not include the labelling period as a fixed term (H2) and the model that excludes the redox zonation as a fixed term (H3).

Table 27: comparison between model H0 and H1

Model	df	AIC	BIC	logLik	Test	L.Ratio	p-value
1	13	-7627	-7568	3827			
2	11	-7586	-7536	3804	1 vs 2	45.2	0

Table 28: comparison between model H0 and H2

Model	df	AIC	BIC	logLik	Test	L.Ratio	p-value
1	13	-7627	-7568	3827			
2	10	-7567	-7521	3794	1 vs 2	66	0

Table 29: comparison between model H0 and H3

Model	df	AIC	BIC	logLik	Test	L.Ratio	p-value
1	13	-7627	-7568	3827			
2	9	-7535	-7494	3776	1 vs 2	100.5	0

Testing showed that each of the terms is significant and thus no terms can be removed from the model. Therefore the final model is refitted with REML to check if the variances are equal and thus if the model is valid.

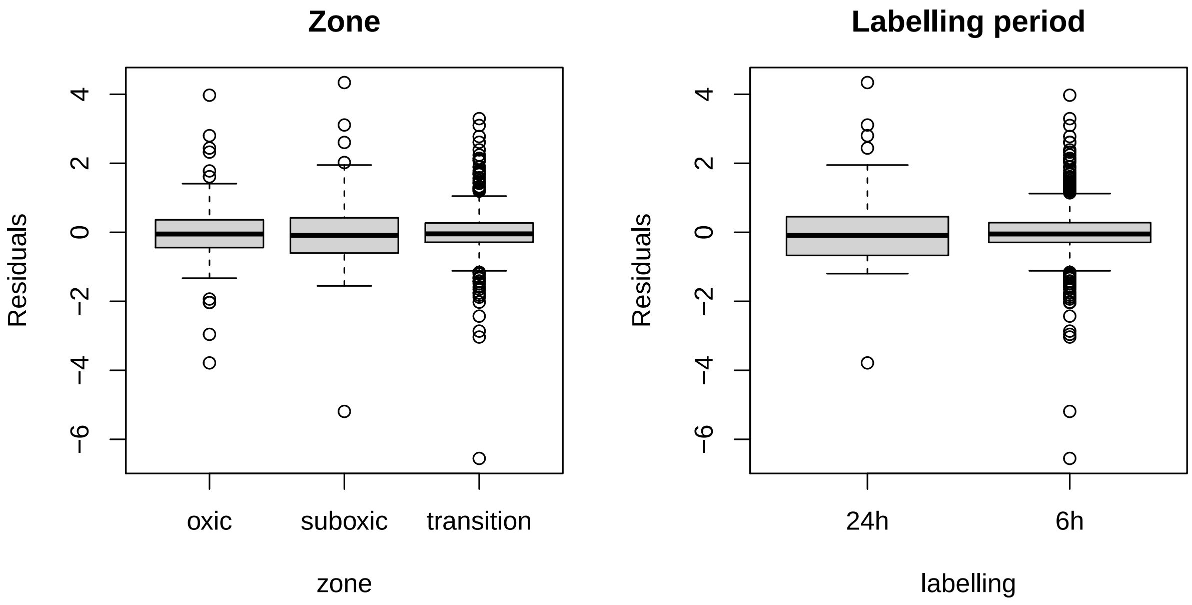

Figure 11: Residuals of the final model as a function of redox zonation and labelling period

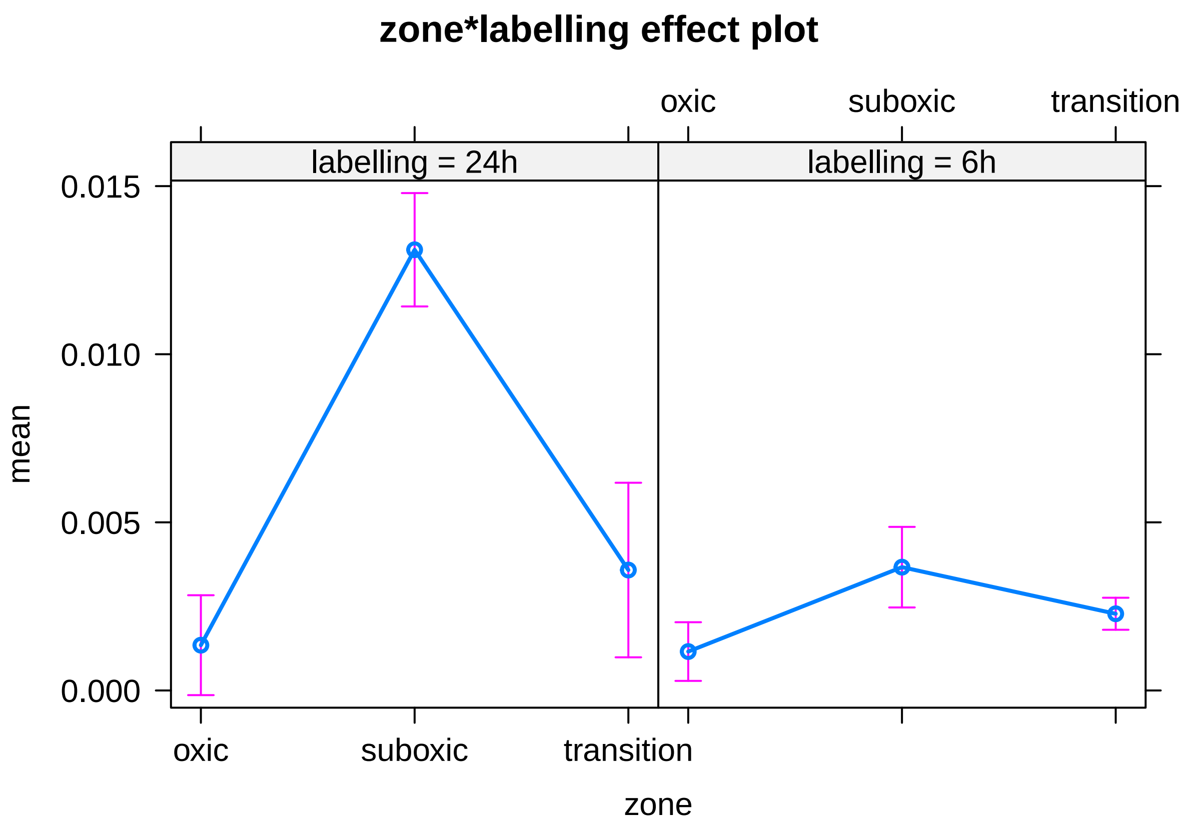

Figure 12: Effect plot showing the modelled values for 13C of each combination of redox zonation and labelling period

Table 30: outcome of model D0 with the oxic zone 6h labelling as a reference

effect	group	term	estimate	std.error	df	statistic	p.value
fixed	fixed	(Intercept)	0.00116	0.00044	540	2.60419	0.00946
fixed	fixed	zonesuboxic	0.00251	0.00075	158	3.32516	0.00110
fixed	fixed	zonetransition	0.00112	0.00051	158	2.22090	0.02778
fixed	fixed	labelling24h	0.00019	0.00088	158	0.21561	0.82957
fixed	fixed	zonesuboxic:labelling24h	0.00925	0.00137	158	6.74772	0.00000
fixed	fixed	zonetransition:labelling24h	0.00111	0.00161	158	0.69224	0.48981
ran_pars	label	sd_(Intercept)	0.00226	NA	NA	NA	NA
ran_pars	Residual	sd_Observation	0.00065	NA	NA	NA	NA

Table 31: outcome of model D0 with the transition zone 6h labelling as a reference

effect	group	term	estimate	std.error	df	statistic	p.value
fixed	fixed	(Intercept)	0.00228	0.00024	540	9.40856	0.00000
fixed	fixed	zoneoxic	-0.00112	0.00051	158	-2.22090	0.02778
fixed	fixed	zonesuboxic	0.00139	0.00066	158	2.11057	0.03638
fixed	fixed	labelling24h	0.00130	0.00134	158	0.96735	0.33485
fixed	fixed	zoneoxic:labelling24h	-0.00111	0.00161	158	-0.69224	0.48981
fixed	fixed	zonesuboxic:labelling24h	0.00814	0.00171	158	4.76584	0.00000
ran_pars	label	sd_(Intercept)	0.00226	NA	NA	NA	NA
ran_pars	Residual	sd_Observation	0.00065	NA	NA	NA	NA

Table 32: outcome of model D0 with the suboxic zone 6h labelling as a reference

effect	group	term	estimate	std.error	df	statistic	p.value
fixed	fixed	(Intercept)	0.00367	0.00061	540	6.00912	0.00000
fixed	fixed	zonetransition	-0.00139	0.00066	158	-2.11057	0.03638
fixed	fixed	zoneoxic	-0.00251	0.00075	158	-3.32516	0.00110
fixed	fixed	labelling24h	0.00944	0.00105	158	8.96131	0.00000
fixed	fixed	zonetransition:labelling24h	-0.00814	0.00171	158	-4.76584	0.00000
fixed	fixed	zoneoxic:labelling24h	-0.00925	0.00137	158	-6.74772	0.00000
ran_pars	label	sd_(Intercept)	0.00226	NA	NA	NA	NA
ran_pars	Residual	sd_Observation	0.00065	NA	NA	NA	NA

Table 33: outcome of model D0 with the suboxic zone 6h labelling as a reference

effect	group	term	estimate	std.error	df	statistic	p.value
fixed	fixed	(Intercept)	0.00135	0.00076	540	1.77931	0.07575
fixed	fixed	zonesuboxic	0.01176	0.00114	158	10.27657	0.00000
fixed	fixed	zonetransition	0.00224	0.00152	158	1.46731	0.14428
fixed	fixed	labelling6h	-0.00019	0.00088	158	-0.21561	0.82957
fixed	fixed	zonesuboxic:labelling6h	-0.00925	0.00137	158	-6.74772	0.00000
fixed	fixed	zonetransition:labelling6h	-0.00111	0.00161	158	-0.69224	0.48981
ran_pars	label	sd_(Intercept)	0.00226	NA	NA	NA	NA
ran_pars	Residual	sd_Observation	0.00065	NA	NA	NA	NA

Table 34: outcome of model D0 with the suboxic zone 6h labelling as a reference

effect	group	term	estimate	std.error	df	statistic	p.value
fixed	fixed	(Intercept)	0.01311	0.00086	540	15.26457	0
fixed	fixed	zoneoxic	-0.01176	0.00114	158	-10.27657	0
fixed	fixed	zonetransition	-0.00953	0.00158	158	-6.04169	0
fixed	fixed	labelling6h	-0.00944	0.00105	158	-8.96131	0
fixed	fixed	zoneoxic:labelling6h	0.00925	0.00137	158	6.74772	0
fixed	fixed	zonetransition:labelling6h	0.00814	0.00171	158	4.76584	0
ran_pars	label	sd_(Intercept)	0.00226	NA	NA	NA	NA
ran_pars	Residual	sd_Observation	0.00065	NA	NA	NA	NA

3.3 The final model

The optimal model includes the fixed parameters redox zonation, labelling period and their interaction. Thus, the final model includes these three fixed terms, one random components where cells are nested into filaments and a variable variance structure:

$$excess\left(\frac{^{13}C}{^{12}C+^{13}C}\right)_{ij} = \alpha+(\beta_1\times zone_{ij}+\beta_2\times labelling\ period_{ij}+\beta_3\times zone_{ij}\times labelling\ period_{ij})+a_j+\epsilon_{ij}$$

This is a random intercept model where the average intercept is denoted by the term α. How the intercept varies as a function of the filament is denoted in the term a_j, where the index j denotes the filament. The term a_j is normally distributed with $N(0,\sigma_j^2)$. The term ϵ_{ij} is the unexplained error and because a multiple variance structure was added this value is different for each combination of the explanatory variables zone and labelling period. Thus, $\epsilon_{ij} \sim N(0,\sigma_m^2)$ where m denotes the different values for the variances for each of the six combinations of redox zone and labelling period (e.g. oxic zone 6h labelling). The output from model 2 gives values of the modelled excess ^{13}C values for each combination of labelling period and zone $(\beta_1 \times zone_{ij} + \beta_2 \times labelling\ period_{ij} + \beta_3 \times zone_{ij} \times labelling\ period_{ij})$.

The model output shows that the random effect a_j, representing the between filament variation is $N(0, 0.002263248^2)$.The random noise (ϵ_{ijk}) is variable and dependent on the combination of redox zonation and labelling period. These values can be used to calculate the intraclass correlation ($ICC_{filament}$). The formula is as follows:

$$ICC_{filament} = \frac{\sigma^2_{filament}}{\sigma^2_{filament}+\sigma^2}$$

Because σ^2 varies per zone and labelling period, the ICC is different for each combination of zone and labelling period.

	zone	σ^2	$ICC_{filament}$
6h	oxic	0.00018	0.994
6h	transition	0.00057	0.940
6h	suboxic	0.00065	0.923
24h	oxic	0.00039	0.970
24h	transition	0.00060	0.935
24h	suboxic	0.00689	0.097

References

Pinheiro, José, Douglas Bates, and R-core. 2022. *Nlme: Linear and Nonlinear Mixed Effects Models.* https://svn.r-project.org/R-packages/trunk/nlme/.

Zuur, Alain F., Elena N. Ieno, Neil Walker, Anatoly A. Saveliev, and Graham M. Smith. 2009. *Mixed effects models and extensions in ecology with R.* Statistics for Biology and Health. New York, NY: Springer New York. https://doi.org/10.1007/978-0-387-87458-6.
